# Cortical-like mini-columns of neuronal cells on zinc oxide nanowire surfaces

**DOI:** 10.1038/s41598-019-40548-z

**Published:** 2019-03-11

**Authors:** V. Onesto, M. Villani, R. Narducci, N. Malara, A. Imbrogno, M. Allione, N. Costa, N. Coppedè, A. Zappettini, C. V. Cannistraci, L. Cancedda, F. Amato, Enzo DI Fabrizio, F. Gentile

**Affiliations:** 1 Center for Advanced Biomaterials for HealthCare, Istituto Italiano di Tecnologia, 80125 Naples, Italy; 2 Department of Experimental and Clinical Medicine, University of Magna Graecia, 88100 Catanzaro, Italy; 3 IMEM-CNR Parco Area delle Scienze 37/A, 43124 Parma, Italy; 4 Istituto Italiano di Tecnologia, Via Morego 30, 16163 Genova, Italy; 5 Tyndall National Institute, Cork, T12 R5CP Ireland; 6 PSE division, King Abdullah University of Science and Technology, Thuwal, 23955−6900 Saudi Arabia; 7 Health Department, University of Magna Graecia, 88100 Catanzaro, Italy; 8 Biomedical Cybernetics Group, Biotechnology Center (BIOTEC), Center for Molecular and Cellular Bioengineering (CMCB), Center for Systems Biology Dresden (CSBD), Department of Physics, Technische Universität Dresden, Tatzberg 47/49, 01307 Dresden, Germany; 9 Brain Bio-Inspired Computing (BBC) Lab, IRCCS Centro Neurolesi “Bonino Pulejo”, Messina, 98124 Italy; 10 Dulbecco Telethon Institute, Rome, Italy; 11 Department of Electrical Engineering and Information Technology, University Federico II, Naples, Italy

## Abstract

A long-standing goal of neuroscience is a theory that explains the formation of the minicolumns in the cerebral cortex. Minicolumns are the elementary computational units of the mature neocortex. Here, we use zinc oxide nanowires with controlled topography as substrates for neural-cell growth. We observe that neuronal cells form networks where the networks characteristics exhibit a high sensitivity to the topography of the nanowires. For certain values of nanowires density and fractal dimension, neuronal networks express small world attributes, with enhanced information flows. We observe that neurons in these networks congregate in superclusters of approximately 200 neurons. We demonstrate that this number is not coincidental: the maximum number of cells in a supercluster is limited by the competition between the binding energy between cells, adhesion to the substrate, and the kinetic energy of the system. Since cortical minicolumns have similar size, similar anatomical and topological characteristics of neuronal superclusters on nanowires surfaces, we conjecture that the formation of cortical minicolumns is likewise guided by the interplay between energy minimization, information optimization and topology. For the first time, we provide a clear account of the mechanisms of formation of the minicolumns in the brain.

## Introduction

The propensity of simple elements to aggregate into clusters is observed at diverse length scales ranging from the sub-atomic level, where combinations of three quarks form baryons^1,2^, to the super-molecular level, where large macro-molecules are composed by smaller repeated units as in DNA^3^, proteins^4^, or synthetic plastics^5–7^, to the scale of the Universe, where thousands of stars congregate in galaxies^8,9^. In complex systems of a great multitude of components, cooperation among components and the emergence of collective phenomena yield unique properties that are not generally expressed by components of those systems taken in isolation^10–13^. Ferromagnetism^14,15^, phonon modes of an atomic lattice^16,17^, surface enhanced Raman spectroscopy^18,19^, superconductivity^20,21^, memory and cognitive functions of the brain^22,23^, depend less on the specialization of individual elements and more on the fact that these elements interact in long-range structures. These functions are thus *scale* dependent and generally break down at small cluster sizes^24–27^. Natural and artificial systems of many interacting elements can be represented as networks^28–30^, where nodes are the system’s components and links describe the actions between elements. Network science is rapidly developing and is at the basis of new data-based mathematical models that are redefining the classical reductionism paradigm^31^.

Sparse systems of pre-existing components may form compact, organized structures via spontaneous self-assembly^32–35^, i.e. without external direction, or triggered by external forces or fields^36,37^. Nanoscale architectures can direct, control and, in some cases, improve the organization of simple elements into clusters^38–43^. Nano-topography is especially important in guiding cell fate at the bio-interface, and is therefore of interest in neural tissue engineering, bio computing, biosensors operations and neural cell based sensors, the diagnosis and analysis of neurodegenerative disorders, neural development^44–46^. In previously reported studies^39,47,48^, we examined patterns of neuroblastoma N2A cells on meso-porous silicon. We observed that N2A cells on a surface with nano-scale motifs display an increased ability to create patterns in which the nodes of the patterns form highly clustered groups and the elements of the groups are connected by a finite, and generally low, number of steps. Networks with similar characteristics are named *small world* networks^49,50^. In ref.^46^ we demonstrated that neural networks with a small world topology on rough silicon substrates feature enhanced signal propagation speed and computational capabilities compared to regular grids of the same size on flat surfaces.

Here, we present zinc oxide nanowire surfaces with variable nanowire density that achieve a tight interface with hippocampal neurons to direct their assembly into clusters. Resulting neuronal networks show a high sensitivity to the geometrical characteristics of the nanowires. For certain combinations of fractal dimension and nanowires density, neurons accumulate into clusters with ~200 neurons per cluster and a small world topology, very similar to the structure of the minicolumns in the cerebral cortex. Cortical minicolumns are the elementary computational units of the mature neocortex. Since superclusters of neural cells emerge from the interplay between mechanical equilibrium, information optimization and topology - that are activated in turn by the nano-scale characteristics of the nanowires surface - we conjecture that the formation of cortical mini-columns is guided by the same mechanisms, and that the geometrical form, structure and size of mini-columns are essential to their functioning. By assembling into mini-columns, i.e. complex circuits with some internal correlation, neurons show properties that are absent in the single neurons per se. The supercluster density that we have observed in our experiments might represent a physical limit below which correlation between neurons is degraded, with reduced convenience to assemble into clusters. This new concept could explain why neurons in the brain evolved into structures with a finite size.

The outline of the paper is the following:

1. We use advanced ZnO nanowires surfaces for cell culturing.
2. We observe the emergence of superclusters of neural cells and analyze the maximum number of cells in a cluster in relation to the topography of the substrate. Using mathematical modelling and numerical simulations, we demonstrate that the maximum allowable cluster size is linked to the characteristics of the system.
3. We provide a complete description of neuronal networks characteristics, using topological measures of networks including clustering coefficient, characteristic path length, small-world-ness, structural consistency. Consistency among measures assure correctness of the analysis and support the notion that neuronal networks on nano-scale surfaces adopt a small world configuration.
4. Using simulations, we show that neurons in small world networks achieve computational performance.
5. In light of these results, for the first time, we provide a clear account of the mechanisms of formation of the mini-columns in the brain.

## Results

### Generating ZnO nanowire surfaces

ZnO nanowires display potential in a number of emerging areas such as low-voltage and short-wavelength optoelectronics, photonics, piezoelectric energy harvesting^51–53^. ZnO nanowires can be assembled on a surface with tight control over the size, shape and density of the wires, differently from randomly generated rough surface as those presented in our previous works^46^. Thus, they guarantee maximum control over the topography of the system. In the analysis of the interaction of neural cells with surfaces, where nano-cues of the surface direct cell behavior, precise control over the characteristics of the surface at the nano-scale level assure repeatability, reproducibility and robustness of the experiments. Moreover, differently from other materials, ZnO nanowires surfaces possess technology significance and potentials. Due to their piezoelectric characteristics, ZnO nanowires may respond to externally applied mechanical stress and can be used as constituents of a force-sensor. In a more sophisticated evolution of the system that will be developed over time, arrays of ZnO nanowires will interact with clusters of neural cells to register forces exchanged with the substrates and the evolution of the energy at the solid-cell interface in real time with elevated spatial resolution, elevated force resolution, and fast response times.

**Figure 1:**
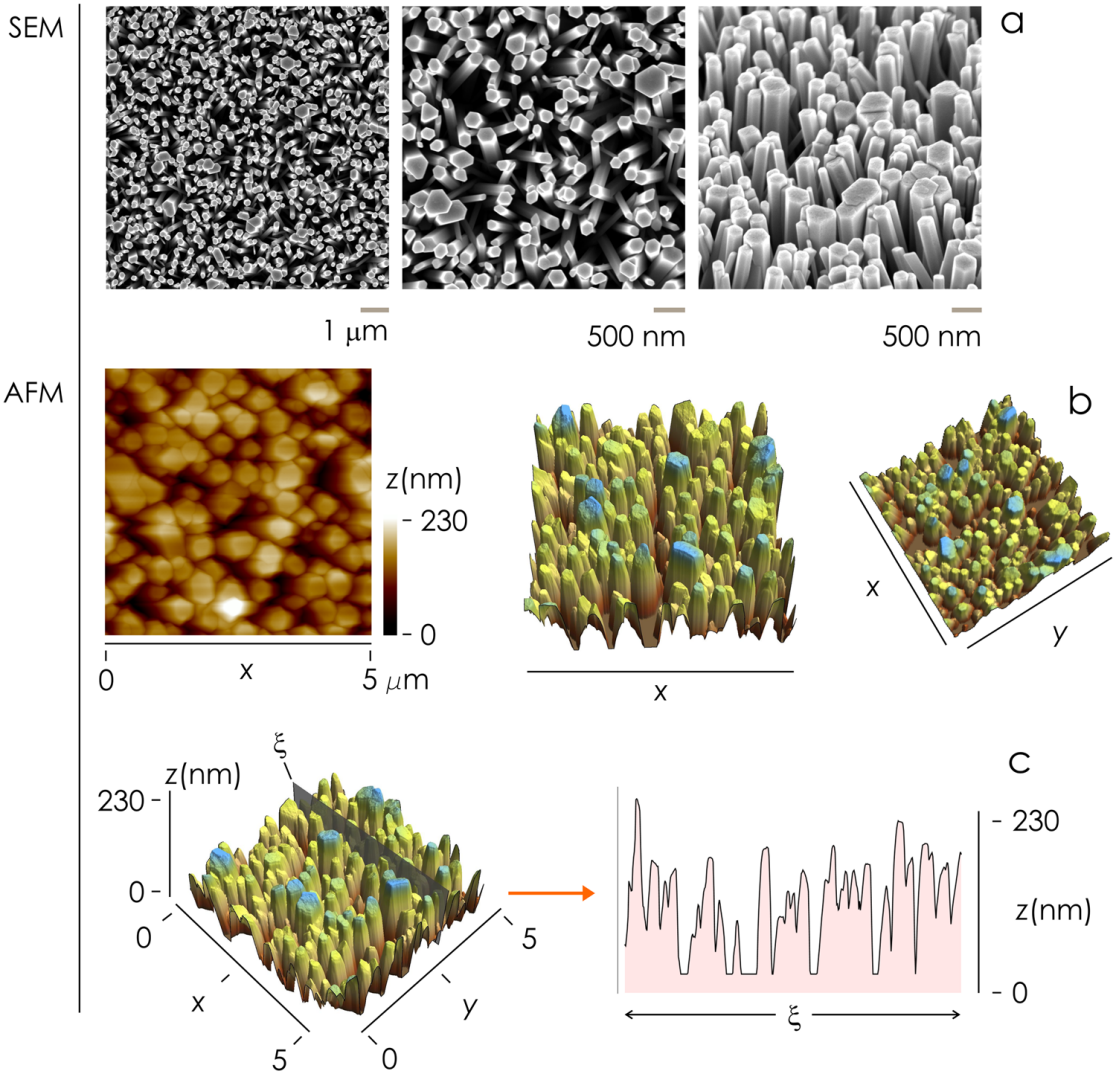
SEM (**a**) and AFM (**b**) images of zinc oxide nanowires surfaces. Nanowires are quasi-vertically aligned with a measured total height that can surpass 200 *nm*. Spacing, density and morphology of the nanowires can be tuned changing the conditions of chemical synthesis.

**Figure 2:**
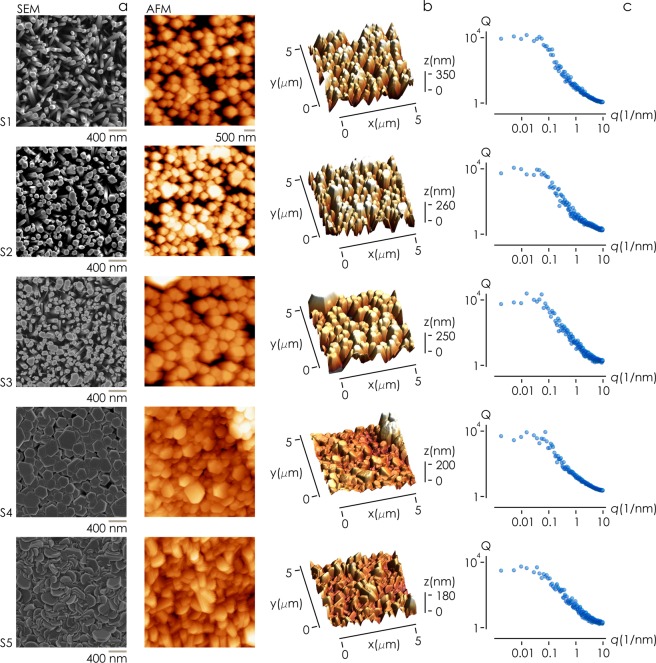
Nanowire surfaces were synthetized using hydrothermal reaction with zinc nitrate hexa-hydrate, hexa-methyl-entetramine and sodium chloride; we produced surfaces with variable density changing the proportions of reagents in the solution. Surfaces were imaged using SEM (**a**) and AFM microscopy (**b**). AFM images were elaborated to derive the power spectrum density function associated to each surface (**c**). A power spectrum delivers the information content of a surface for change of scale.

Here, upon seeding an initial ZnO layer on a glass substrate, nanowires were grown by Chemical Bath Deposition (CBD) using zinc nitrate as Z*n* source, hexamethylentetramine (HMTA) as shape directing agent, and sodium chloride as a source of chloride anions to further tune the aspect ratio^54,55^ (methods and Supporting Information 1). The method enables to produce arrays of vertically aligned nanowires with tight control over the shape and size of the wires (Fig. 1). Varying the reaction conditions of growth (Supporting Information 1) we produced five different nanowire sample types, S_1_–S_5_, with variable nanowires density and surface characteristics. We sorted sample list in order of nanowires density, thus the density of nanowires on sample surface smoothly transitions from low values of density for S_1_ to high values of density for S_5_, as explained in the following of the section (Fig. 2).

We used Scanning Electron Microscopy (SEM) (Fig. 2a) and Atomic Force Microscopy (AFM) (Fig. 2b) for imaging the morphology of the samples at the nanoscale. AFM achieves ultra-high resolution and can reproduce sample profile with sub-nanometer resolution in z and nanometer resolution in x, y. Since AFM encodes quantitatively the information on the topography of a surface, we used AFM to extract mathematical variables of samples including roughness and fractal dimension. We used top-view SEM images to derive the density of the nanowires and cross sectional SEM image to derive the internal structure of the samples. We fabricated at least 5 different samples per each nanowires density and acquired more than 25 SEM images and more than 5 AFM images per sample. Additional SEM and AFM images of samples are reported in a separate Supporting Information 2.

AFM images of sample surfaces were used to derive the characteristic power spectrum density function for each sample (Fig. 1c). A power spectrum is a function *Q* of the spatial frequencies *q* of a sample: it reports the amount of detail of a surface contained at a specific *q* (Methods). Since it delivers the information content of a surface per change of scale, it shall be used in the following to extract the *fractal dimension* of nanowires surfaces^56^.

**Figure 3:**
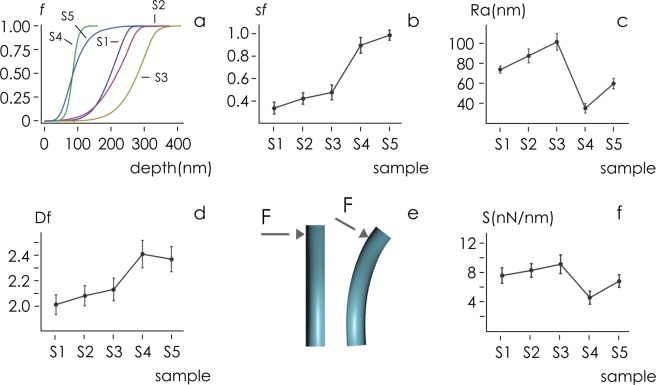
AFM images of nanowire surface were analyzed to extract topography data of samples. The fraction of AFM data points above a given depth indicates that sample height distribution and nanowire density smoothly increases moving from samples S_1_ to sample S_3_; for samples S_4_ and S_5_, nanowire height distribution falls to lower values (**a**). This is reflected by nanowire density on a surfarce that sharply increases to ~1 for samples S_3_, S_4_, S_5_. (**b**). Roughness of analyzed samples steadily increases for the first three samples to reach a maximum Ra ~ 100 nm for sample S_3_, followed by a severe reduction for samples S_4_, S_5_ (**c**). In contrast, Fourier analysis of samples shows that maximum surface fractal dimension is expressed from sample S_4_ (D_f_ ~ 2.4) and S_5_ (D_f_ ~ 2.3) (**d**). AFM mechanical characterization of samples indicates that sample stiffness is relevant (>4nN/nm) for all considered samples whereby nanowire deformation can be neglected (**e**,**f**).

We processed AFM data to derive per each sample the corresponding Abbott-Firestone curve (Fig. 3a). The Abbott-Firestone curve describes the surface texture of a sample. It is the cumulative probability density function *f* of the surface profile’s height *z* - it describes the percentage of points *f* of a surface that is above the height *z*. The form of *f*(*z*) is indicative of the topographic characteristics of a sample. The diagram in Fig. 3a indicates that the maximum nanowire height is ~350 nm. For samples S_1_ and S_2_, approximately 50% of sample’s profile has a measured height greater or equal than ~200 nm and ~210 nm respectively, in contrast to a measured height greater than or equal to ~300 nm for sample S_3_. This indicates that sample’s height distribution and thus nanowire density on a surface smoothly increases moving from samples S_1_ to sample S_3_. For samples S_4_ and S_5_, nanowire density approaches unity, nanowire gap becomes vanishingly small and measured AFM profile reproduces surface asperities rather than individual nanowire contours.

We used image analysis algorithms and the methods described in the Supporting Information 3 to extract from the top-view SEM images of samples the solid fraction of the nanowires substrates (*sf*). We define the solid fraction as the fraction of solid in a nanowires structure occupied by constituent nanowires computed at the upper boundary of the structure. It is comprised between 0 and 1. In the hypothesis that the cross-sectional profile of the nanowires does not vary along the nanowires height (z), the solid fraction is an estimate of the bulk *density* of the nanowires structure. (Image analysis methods applied to cross sectional SEM images of nanowires structures confirm that values of *solid fraction* match with those of *density* for all considered samples with a good accuracy - Supporting Information 3 - therefore in the following we will use interchangeably the terms *solid fraction* and *density*). Solid fraction of samples is reported in Fig. 3b for all sample types. We observe that *sf* varies steadily from *sf* = 0.34 ± 0.053 for S_1_ to *sf* = 0.426 ± 0.051 for S_2_ to *sf* = 0.48 ± 0.066 for S_3_. Then, *sf* sharply increases to *sf* = 0.90 ± 0.072 for S_4_ and *sf* = 0.99 ± 0.046 for S_5_. Data indicate that nanowires are loosely dense for samples S_1_, S_2_ and S_3_, and highly densely packed for samples S_4_ and S_5_.

We calculate a *r*-squared statistic *r*^2^ to test whether the data in the first band (S_1_–S_3_) are consistent with a linear regression. Values of *r*^2^ near unity, of estimated variance *σ* near zero, and of P value below 0.05, indicate that *sf* is linear for the first three samples (*r*^2^ = 0.98, *σ* = 0.00017, *p* = 0.043).

We then determined the average roughness of nanowires surfaces (*Ra*) to provide additional details on the topography of samples. The average roughness is a measure of the deviations of a profile from its mean value calculated over the entire sample surface. Diagram in Fig. 3c indicates that *Ra* increases *linearly* from *Ra* = 74 ± 3.4 nm for S_1_ to *Ra* = 88 ± 7 nm for S_2_ to *Ra* = 102 ± 8.2 nm for S_3_ (*r*^2^ = 1, *σ* = 10^−23^, *p* = 2.48 × 10^−15^). This is ascribed to the fact that in an array of pillars, a variation in roughness is associated to a change of the density of the pillars. *Ra* displays a discontinuity and a severe reduction at S_4_ (*Ra* = 35 ± 4.74 nm), followed by a partial recovery of roughness to *Ra* = 60 ± 5.4  nm for S_5_.

We used the power spectrum density function reported in Fig. 2c to derive the fractal dimension *D*_*f*_ of samples (Methods). The fractal dimension is an index that quantifies the degree of complexity of a surface. It is related to the change of detail to the change of scale of a surface. The larger the amount of information contained across different scales, the larger *D*_*f*_. Euclidian smooth surfaces have *D*_*f*_  = 2. Determining *D*_*f*_ is relevant because it has been demonstrated that adhesion of cells to a surface is correlated to sample fractal dimension^57^. Data in Fig. 3d show that *D*_*f*_ varies linearly from *D*_*f*_ = 2.04 ± 0.078 for S_1_ to *D*_*f*_ = 2.08 ± 0.08 for S_2_ to *D*_*f*_ = 2.13 ± 0.09 for S_3_ (*r*^2^ = 0.99, *σ* = 0.00067, *p* = 0.048). Samples S_4_ and S_5_ exhibit the maximum values of fractal dimension, i.e. *D*_*f*_ = 2.41 ± 0.109 for S_4_ and *D*_*f*_ = 2.37 ± 0.11 for S_5_. Notice that in the limit of small nanowires density (*sf* < 0.5, samples S_1_–S_3_) the topographic measures of the surfaces *sf*, *Ra* and *D*_*f*_, show internal consistency. To an increase of *sf* is associated an increase of *Ra* and *D*_*f*_. In the high nanowires density regime (*sf* ~ 1(, samples S_4_ and S_5_) values of *Ra* are small, while high values of fractal dimension *D*_*f*_ may be more effective in describing the complex nature of samples surface.

Mechanical stiffness of nanowires was verified using AFM (Fig. 3e and Methods). We found that the stiffness S of nanowires ranges between a minimum S = 4.45 ± 0.9 *nN/nm* measured for sample S_4_ and a maximum S = 9.03 ± 1.2 *nN/nm* measured for sample S_3_ (Fig. 3f). Considering that traction forces exerted by cells sitting on a layer of microneedles is ~5*nN* per square micrometer of active focal adhesion sites on that layer^58–60^, for the present configuration cultured neural cells would displace laterally the nanowires of fractions of nanometers solely. Therefore, sample deformability has been disregarded in the remaining of the analysis.

### Culturing neurons on nanowire surfaces

**Figure 4:**
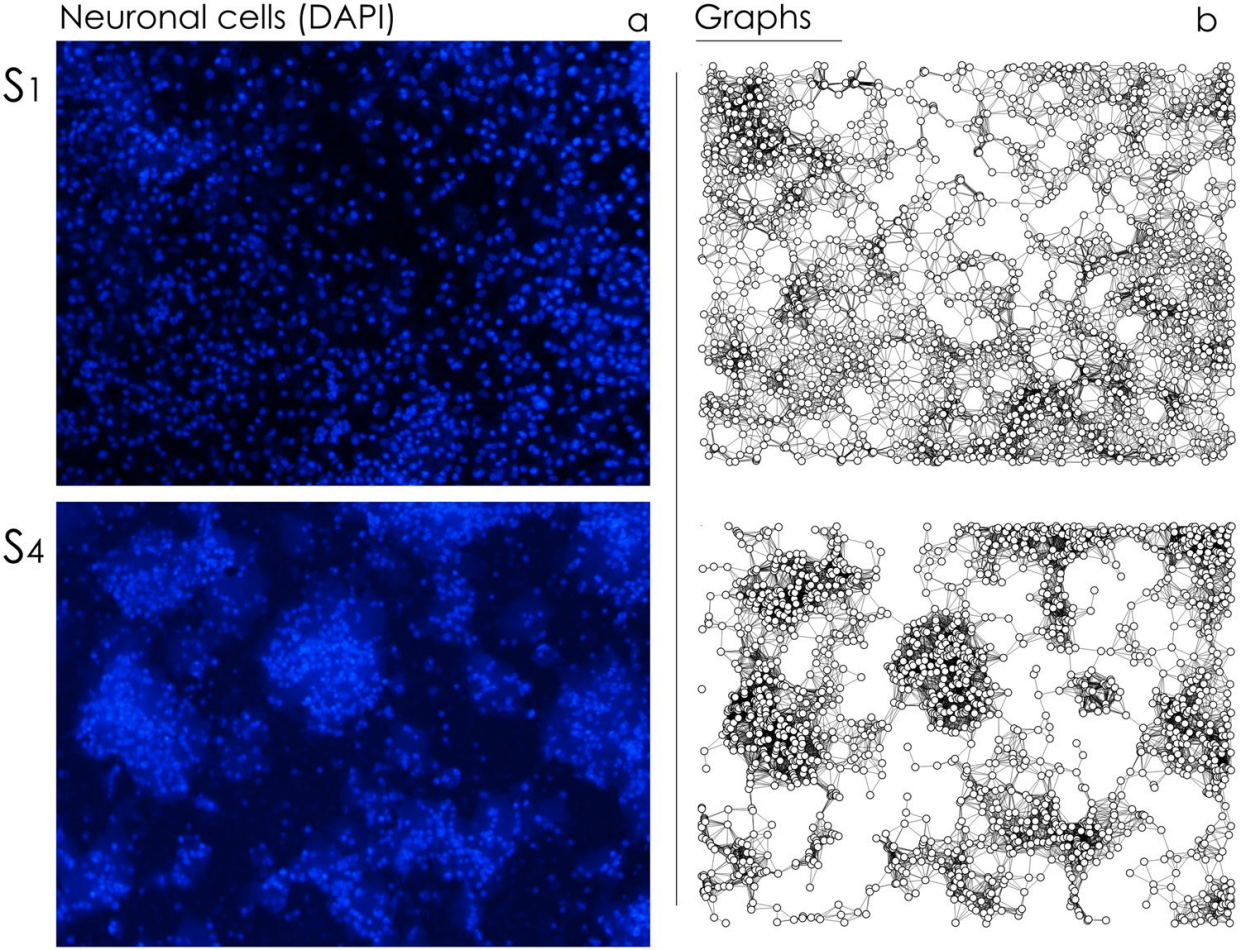
We cultured primary hippocampal neural networks on nanowires. Fluorescence images of cells (**a**) and corresponding wiring diagrams obtained by applying a Waxman algorithm to the cells (**b**) show the topology of the networks is influenced by the density and geometrical characteristics of the nanowires.

We cultured neuronal cells on samples S_1_–S_5_ with an initial cell density of 10^5^ cells per cm^2^ (Methods). 7 days after seeding (DIV 7) we halted neural growth, fixed the cells and examined network topology using DAPI staining and fluorescence microscopy (Fig. 4a). We then determined the positions of the nuclei (nodes) using image analysis algorithms described in previous works^57^. Nodes were used to construct the *topological* graphs associated to neural networks cultured on different substrates (Fig. 4b).

In constructing the graphs, we used the Waxman algorithm. The selection of this model is not arbitrary, and is motivated by the fact that the Waxman model was specifically designed for routing of multipoint connections^61^ and it generates a network structure that (such as in the case of structural brain networks) allows efficient information transfer across the network nodes^62^. The research on models for reconstruction of neuronal microscale networks is at the beginning (we are studying also new models to accomplish this task), yet the Waxman model offers a reasonable network structure approximation to emulate propagation of information between neuronal nodes. Specifically, the Waxman model wires couples of nodes based on their geometrical distance. The probability of wiring of two points in this model depends on two arbitrary parameters: α, which is a *temperature* that for higher values allows geometrically larger random connection; and *β*, which controls the node degree. Hence, we computed a set of topological measurements of the networks^62,63^ where the variables of the model were varied over wide intervals (Supporting Information file 4). Then we chose the optimal parameters of the models as *α* = 1, *β* = 0.025, that are those variables that produce a similar, concurrent output of topological measures of networks across different landscapes.

**Figure 5:**
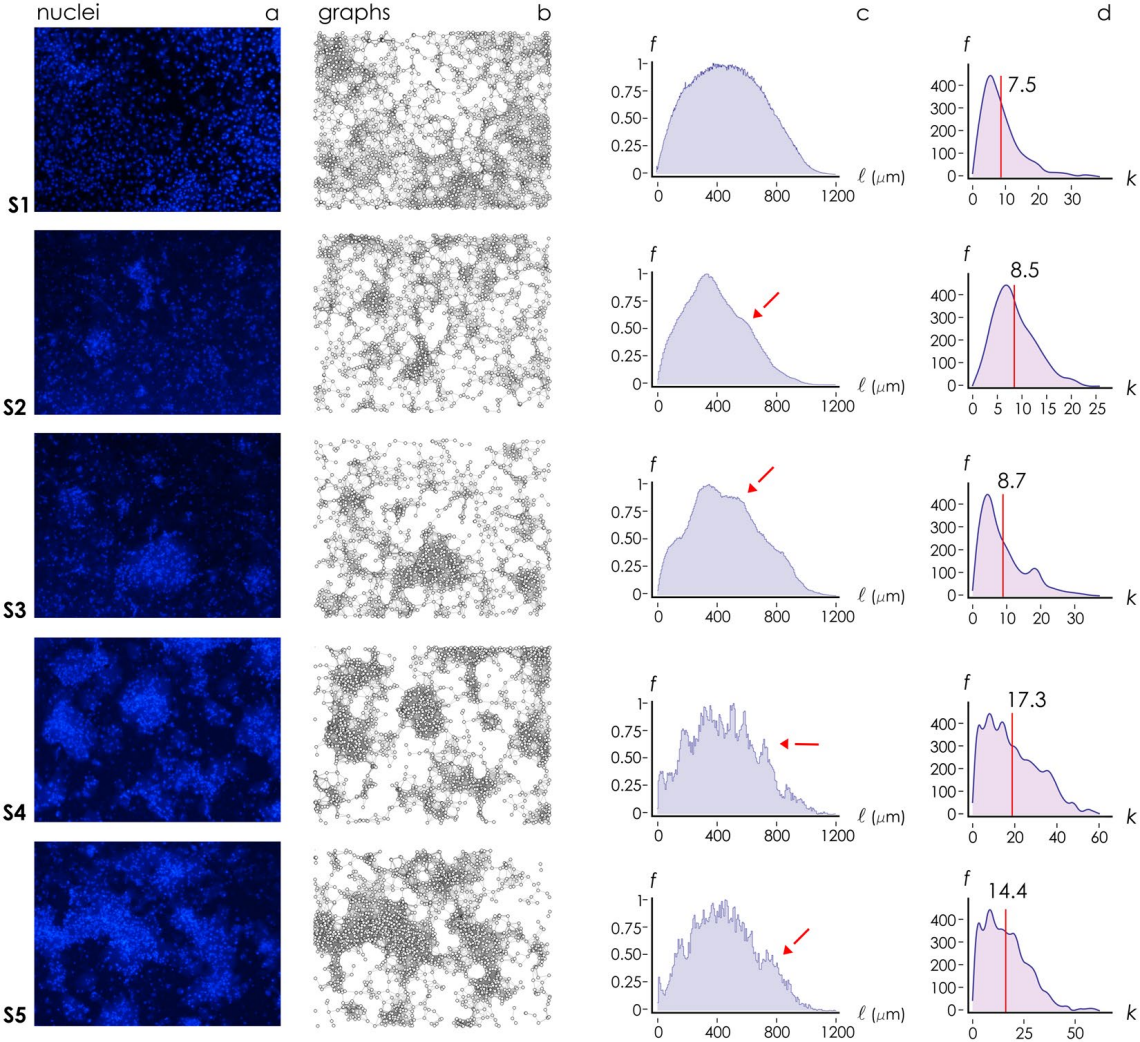
Primary hippocampal neural networks were cultured on nanowire surfaces. Using fluorescence microscopy, we imaged cell nuclei for each substrate preparation (**a**). Fluorescence images of cells were analyzed to extract cell positions and network topology for all samples (**b**). Cell-cell distance (**c**) and degree of a graph (**d**) distributions indicate that cells form highly clustered groups on sample surface for sufficiently high values of nanowire density (S_3_–S_5_).

In analyzing Fig. 4, where cells are reported for low (S_1_) and large (S_4_) values of nanowires density, we observe that cell clustering and the characteristics of the networks are influenced by substrate preparation. This behavior is maintained for each substrate. We report in Fig. 5 panels with fluorescence images of cells cultured on nanowires for all the considered range of samples, from S_1_ to S_5_. We report in a separate Supporting Information 5 individual, large format, high-resolution images of cells on substrates and corresponding wiring diagrams.

We observe that cells are uniformly distributed on the surface for S_1_ and S_2_. Moving from sample S_1_ to sample S_5_, cells gradually form fewer clusters with a larger numbers of elements per cluster (Fig. 5a). For samples S_3_–S_5_ with sufficiently high values of nanowire density and fractal dimension, neurons within groups of cells are preferentially interconnected (Fig. 5b). For substrates with the characteristics of high *sf* and high *D*_*f*_, anatomical connectivity among individual neurons is enhanced, with a small distance between two randomly chosen nodes of the grid (small path length), that in turn suggests a small world architecture of the networks. We then used elements of *graph theory* to examine whether graphs have small world attributes. We introduce here a preliminary cluster analysis of cultured neuronal networks, accompanied in the next section by a more rigorous exposition of topological measures of networks.

Distribution of nodal distances *f*(*l*), i.e. the probability *f* of finding in a network a couple of cells separated by a distance *l*, is reported in Fig. 5c for all the considered samples S_1_–S_5_. Shape of the distribution for sample S_1_ is a Gaussian, unimodal distribution, reflecting the uniform random distribution of cells on the surface, with the most frequent value of the distribution corresponding to the modal distance among elements (intra-cluster distance). For samples S_2_ to S_5_, one may observe the emergence of a second peak in the distribution, that corresponds to modal value of distance between clusters (inter-cluster distance). From the distributions of nodal distances, we extracted the distribution of the degree of a graph for each sample (Fig. 5d). The degree of a graph, *κ*, is the number of edges that is incident to a node, thus the larger *κ* the larger the connectivity of a node.

In the diagrams, the vertical line represents the mean value of *κ* averaged over the distribution. For each substrate, the distribution of *κ* presents a peak, i.e. the most probable number of connections per node in a graph. The networks surpass the small-worldness test (SW values are greater than one as described in the next section) and, analyzing their degree distribution, for large *κ* the probability of finding a highly connected vertex decreases exponentially with *κ* (power-lawness test was negative - Supporting Information 6), therefore these networks obey a small-world model^64^. For the networks shown in Fig. 5b, the average number of connections per node shifts from low (*κ* ~ 7) to high (*κ* ~ 14) values of *κ* moving from S_1_ to S_5_, with a maximum value *κ* ~ 17 observed for sample S_4_.

**Figure 6:**
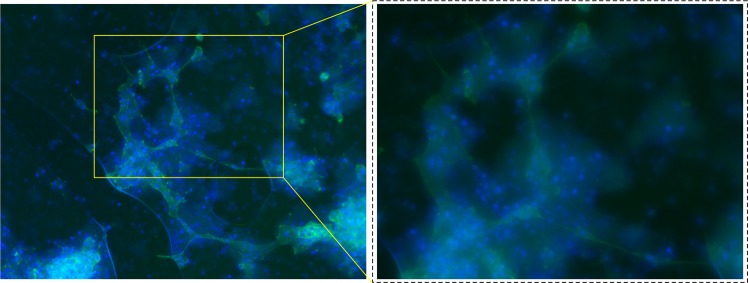
We stained cells with DAPI to determine cell positions on the substrate, and F-Actin microfilaments in the cytoskeleton with phalloidin for imagining neurite branching in neuronal networks. Merge of DAPI and phalloidin images shows colocalization of cell centers with cell protrusions in the cell colonies on substrate S_4_.

To verify the hypothesis that the characteristics of the substrate cause the emergence of small world networks, we stained cells for the cytoskeletal protein F-Actin. We used Phalloidin that selectively binds to and fluorescence microscopy (Methods) for imaging neurites in cultured neural networks. Images of neurite branching acquired for all substrate preparations (Supporting Information 7) and shown in Fig. 6 for sample S_4_, indicate that for substrates with large nanowires density and large fractal dimension (S_3_–S_5_), neuronal networks feature sparse connections inter-cluster and over-abundance of connections intra-cluster, that is indicative of small world architectures.

### Topological characteristics of cultured neuronal networks

We used networks analysis and the method reported in refs^46,48,57^ and in the (Supporting Information file 4) to extract quantitative information from fluorescence images of cells. We analyzed more than 40 images per sample type.

**Figure 7:**
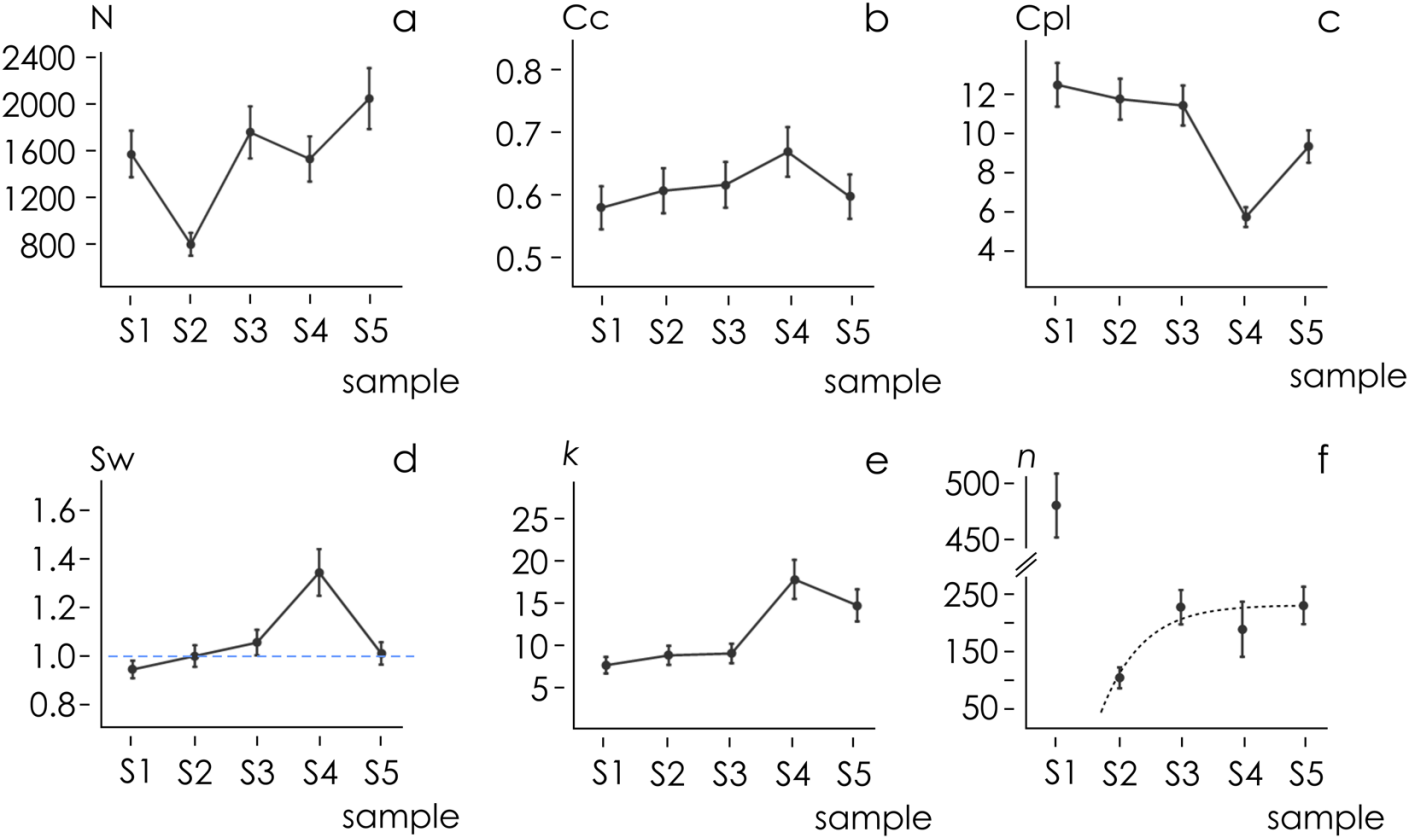
Using image analysis algorithms, we determined the number of adherent neural cells *N* on the nanowire surfaces 7 days after seeding (**a**). Network analysis allowed us to derive the clustering coefficient Cc (**b**), the characteristic path length Cpl (**c**), the small-world-ness coefficient Sw (**d**) and the degree of a graph *k* (**e**) for cultured neural networks as a function of substrate preparation. Cell on sample surfaces S_3_–S_5_ exhibit small world attributes. Moreover, using cluster analysis, we derived the number of clusters of cells as a function of substrate preparation and found that the number of cells per cluster is approximately 200 (**f**).

The number *N* of adhering cells imaged at DIV7 in a region of interest (ROI) of 1 mm per side is reported in Fig. 7a as a function of substrate preparation. We observe that *N* oscillates between *N* = 780 ± 101 neurons per square millimeter for sample S_2_ and *N* = 2066 ± 268 for sample S_5_. *N* is lower for small nanowire density (S_1_–S_2_) and increases with nanowire density (S_3_–S_5_). In all considered cases, cell density and neuron population is high, that guarantees statistical significance of the analysis. Analysis of variance (ANOVA) was used to examine differences among sample means. ANOVA results indicated that the difference among the numbers of nodes across different samples is statistically significant (*p* = 0). Bonferrroni post hoc test conducted on the whole data set the further indicated that there are differences between sample S_1_ and samples S_3_, S_5_ and sample S_2_ and samples S_3_, S_4_, S_5_, with a significance level *p* = 0.01. Same test indicated that there are differences between samples S_3_ and samples S_4_, S_5_, and differences between sample S_4_ and S_5_, with a significance level *p* = 0.05. (${N}^{{{\rm{S}}}_{1}}\ne ({N}^{{{\rm{S}}}_{3}},{N}^{{{\rm{S}}}_{5}})$ and ${N}^{{{\rm{S}}}_{2}}\ne ({N}^{{{\rm{S}}}_{3}},{N}^{{{\rm{S}}}_{4}},{N}^{{{\rm{S}}}_{5}})$ with p = 0.01, ${N}^{{{\rm{S}}}_{3}}\ne ({N}^{{{\rm{S}}}_{4}},{N}^{{{\rm{S}}}_{5}})$ and ${N}^{{{\rm{S}}}_{4}}\ne {N}^{{{\rm{S}}}_{5}}$ with p = 0.05).

The mean clustering coefficient *cc* and characteristic path length *cpl* of graphs are reported in Fig. 7b,c for all considered samples. The clustering coefficient of a graph is the ratio of active links connecting the neighborhood of a node to the total number of its possible connections, averaged over all the nodes of that graph. The characteristic path length is the mean shortest path length between two nodes of a graph. *cc* varies between *cc* = 0.57 ± 0.03 for sample S_1_ and *cc* = 0.667 ± 0.04 for sample S_4_. *Cpl* varies between *Cpl* = 5.667 ± 0.51, measured on sample S_4_, and *Cpl* = 12.51 ± 1.12 measured on sample S_1_. Diagrams indicate the degree of clustering of neuronal cells networks *increases* with nanowires density and fractal dimension and reaches a *maximum* for sample S_4_. Similarly, the mean distance between nodes *decreases* with nanowires density and fractal dimension and reaches a *minimum* for sample S_4_.

Analysis of variance of *cc* data indicate that there is a significant difference among sample means (*p* = 1.4 × 10^−9^). Bonferroni post hoc test indicates that *cc* measured on sample S_4_ is different from values of *cc* measured on remaining substrates, with a significance level *p* = 0.01. ($c{c}^{{{\rm{S}}}_{4}}\ne (c{c}^{{{\rm{S}}}_{1}},c{c}^{{{\rm{S}}}_{2}},c{c}^{{{\rm{S}}}_{3}},c{c}^{{{\rm{S}}}_{5}})$ with *p* = 0.01).

Analogously, analysis of variance applied to *Cpl* data indicates that there is no significant relationship between variables (*p* ~ 0). Bonferroni post hoc test indicates that *Cpl* measured on sample S_4_ and sample S_5_ are different from values of *Cpl* measured on the remaining substrates, with a significance level *p* = 0.01. ($Cp{l}^{{{\rm{S}}}_{4}}$ ≠ $(Cp{l}^{{{\rm{S}}}_{1}},Cp{l}^{{{\rm{S}}}_{2}},Cp{l}^{{{\rm{S}}}_{3}},Cp{l}^{{{\rm{S}}}_{5}})$ and $Cp{l}^{{{\rm{S}}}_{5}}$ ≠ $(Cp{l}^{{{\rm{S}}}_{1}},Cp{l}^{{{\rm{S}}}_{2}},Cp{l}^{{{\rm{S}}}_{3}},Cp{l}^{{{\rm{S}}}_{4}})$ with *p* = 0.01).

Data suggest that both *cc* and *Cpl* of networks correlate to the fractal dimension of substrates. We calculated an r-squared statistic *r*^2^ to test whether the variability of *cc* and *Cpl* can be explained by the independent variable *D*_*f*_. Values of *r*^2^ and estimated variance *σ* indicate that data are consistent with the matching template and the fractal dimension of a surface modulates network assembly, in line with previous reports^46,48^ (${r}_{cc}^{2}=0.972$, *σ*_*cc*_ = 0.00053, *p*_*cc*_ = 0.012, ${r}_{Cpl}^{2}=0.980$, *σ*_*Cpl*_ = 0.031, *p*_*Cpl*_ = 0.010).

Since high clustering and short paths are typical of *small world* networks^49,50^, we used the topological measure *small-world-ness* Sw, defined in ref.^65^ and Supporting Information 4, to examine whether cultured neural networks exhibit *small world* attributes. We found that samples with high values of fractal dimension and nanowires density (S_2_–S_5_) exhibit values of Sw above unity. Specifically, ${{\rm{Sw}}}^{{{\rm{S}}}_{1}}=0.96\pm 0.028$, ${{\rm{Sw}}}^{{{\rm{S}}}_{2}}=1.03\pm 0.034$, ${{\rm{Sw}}}^{{{\rm{S}}}_{3}}=1.08\pm 0.041$, ${{\rm{Sw}}}^{{{\rm{S}}}_{4}}=1.32\pm 0.075$, ${{\rm{Sw}}}^{{{\rm{S}}}_{5}}=1.05\pm 0.036$. Maximum value of Sw is found in correspondence of sample S_4_, in agreement with the trend of *cc* and *Cpl*. ANOVA analysis and Bonferroni post hoc test show that networks on sample S_4_ have values of Sw that are significantly *greater* than values of Sw measured on all other substrates (*p* = 0.01). Same statistical test shows that that networks on sample S_1_ have values of Sw significantly *less* than values of Sw of networks on other substrates (*p* = 0.01). Intermediate values roughness and high values of fractal dimension may be responsible for the propensity of cells to assemble into clustered groups. In ref.^46^, we have demonstrated that roughness breaks unstable equilibrium of cells on a substrate and makes them collapse into more favorable clustered energy states.

Degrees of the distributions *κ* are reported in Fig. 7e for all considered samples. For each network, *κ* indicates the mean number of edges per node. In line with the performance of *cc*, *Cpl* and Sw, *κ* increases with the fractal dimension and the density of the nanowires. Values of *κ* range from *κ* = 7.61 ± 0.98 for sample S_1_, to *κ* = 8.78 ± 1.14 for sample S_2_, to *κ* = 9.01 ± 1.17 for sample S_3_. *κ* reaches a maximum *κ* = 17.86 ± 2.32 for sample S_4_, then it settles to the value *κ* = 14.75 ± 1.91 on sample S_5_. Values of *κ* measured on substrates S_4_ and S_5_ are statistically greater than those measured on substrates S_1_–S_3_ (Bonferroni post hoc test, *p* = 0.01).

### Imaging neurite branching in cultured neuronal networks

Results presented above are based on the position of the nuclei of the cells that are then inter-connected using the Waxman algorithm. It is a conjecture on the form of the real neural network. We performed additional experiments to image the axons and the synapses of the neuronal cells in the networks, resolve their structure, and examine their architecture. We cultured neural cells on the nanowires surfaces S_1_ to S_5_. Then, we used functional calcium imaging (fMCI) techniques to image the topology of neuronal networks. The technique registers the local transients of calcium ions within the networks, which are associated to neuronal activity^66,67^. Thus fMCI analysis may reveal the active synapses of neuronal circuits and the single synaptic connections. Results, reported in the Supporting Information Figure 8.1, indicate that moving from sample S_1_ to sample S_5_, neurons are increasingly more connected to each other, and the number of interconnected neurons in a group increases. This is consistent with the main findings of the work, that certain topographies with high fractal dimension induce neuronal cells to form super-clusters of cells – with small world topology – resembling the architecture of the mini-columns in the cerebral cortex. This suggests that the topological architecture of cells is relevant to their function. Moreover, the same cultures were immune-labeled with primary antibodies recognizing synapsin I, a synaptic vesicle protein localized in presynaptic specializations^68^. Confocal microscopy was utilized to quantify the number of synapsin puncta. Then, the number of synapses per neuron was calculated by dividing the density of synapsin puncta with the density of cells. The trend of this experimental ratio as a function of sample type, reported in Supporting Information Figure 8.1, reflects the form of the *theoretical* degree of the graphs, *κ*, reported in Fig. 7, calculated starting from the cell nuclei distributions.

### Superclusters of neuronal cells on zinc oxide nanowire surfaces

**Figure 8:**
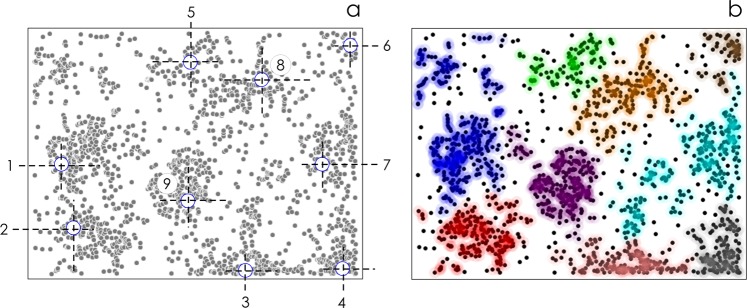
We used density based clustering algorithms to find in each neuronal network cluster centers (**a**). Then, cells were assigned to individual clusters on the basis to their distance to cluster centers. Points are colored according to the group to which they are assigned (**b**).

We determined the number of separate clusters emerging in neuronal networks cultured on each sample using a density-based clustering algorithm, where clusters are recognized regardless of their shape. The clustering procedure, developed recently by Rodriguez and Laio^69^, finds cluster centers as those points with higher density than their neighbors and by a relatively large distance from points with higher densities (Supporting Information 9). Here, we readapted the algorithm to search for the number of groups of adjacent cells in each image (Fig. 8).

We then determine the cell density *n* as the total number of cells to the number of clusters in each image. *n* is the average number of cells per cluster in a network. Values of *n* are reported in Fig. 7e as a function of sample type, S_1_–S_5_. With the exception of the first substrate, S_1_, for which due to elevated cells uniformity the algorithm identifies very few clusters in the images and the estimate of *n* is unreliable, *n* smoothly transitions from nearly 100 cells/cluster for S_2_ to about 200 cells/cluster for S_4_.

Specifically, we found that ${n}^{{{\rm{s}}}_{1}}=478\pm 29$, ${n}^{{{\rm{s}}}_{2}}=105\pm 18$, ${n}^{{{\rm{s}}}_{3}}=226\pm 29$, ${n}^{{{\rm{s}}}_{4}}=189\pm 38$, ${n}^{{{\rm{s}}}_{5}}=229\pm 32$. We used a bounded exponential function of the type *n*(*x*) = *C* + *A*(1 − *Be*^−(*x*−*o*)^) to fit the whole data set excluding sampe S_1_. Best fit of data yields *C* = 50, *A* = 180, *B* = 2, *o* = 1.4 (*r*^2^ = 0.88). Individual raw data, fitted curve, and the 95% confidence intervals for the predicted response are reported in a separate Supporting Information 10. In the latter three substrates, *n* oscillates in the narrow interval *n* = 212 ± 22 cells per cluster. Bonferroni post hoc test indicates that there is no significant relationship between *n* across samples S_3_–S_5_ (*p* = 0.05).

We define neural clusters, with more than 10^2^ elements per cluster, superclusters. Regularity of supercluster size for high-density nanowires samples may be suggestive of universal laws of neural network assembly. We used computer simulations (Supporting Information 11) to explore the effect of cluster size on the stability of neural networks. We generated bi-dimensional clusters of cells where the size of the clusters is varied over a significant range (1–1000 *cells*/*cluster*) and cell density (2000 *cells*/*mm*^2^) is maintained constant. Then, we introduced a ghost cell at an arbitrary position in the domain to measure the overall potential energy *e* produced by the entire system on that position. Moving the probing cell from the center to the periphery of the cluster, we derive the system’s free energy landscape as a function of distance *ρ* from cluster center and associated energy barrier, i.e. difference of potential energy between center and border of the cluster (Supporting Information Figure 11.1). Height of the barrier Δ*e* is proportional to the adhesion strength of individual cells to the cluster and *F*_*e*_ = ∂*e*/∂*ρ* is the force that at any moment a cell has to exert to escape the cluster (Supporting Information Figure 11.2). We found that Δ*e* is positive for low numbers of cells in a cluster and decreases for increasing cluster size (Supporting Information Figure 11.1). For *n* ~ 600 cells in a cluster, *e* becomes prevalently flat with Δ*e* ~ 0 and *F*_*e*_ ~ 0 everywhere in the domain. Results indicate that *n* ~ 600 is an upper limit beyond which binding of additional cells to a cluster is unstable (unfavorable). However, since cells during migration produce forces (*F*_*m*_) in the direction of motion^70–72^, *F*_*m*_ may break the energy barrier and cause cell-cluster ties disruption well below the theoretical limit of 600 cells per cluster. Maximum cluster size is limited by a balance between *F*_*m*_ and *F*_*e*_. Reversing this logic, measure of maximum cluster size on a substrate may indicate the intensity of cell motility forces induced by specific substrate morphologies (Supporting Information Figure 11.3). Here, combining theory and experiments, we determined an estimated value of force during adhesion dependent migration of neurons on nanowire geometries of *F*_*m*_ ~ 2000 pN, in agreement with reported measured values of propelling forces (1–10000 pN) and stresses (~100 Pa) of adherent cells on solid surfaces^57,70–72^. In a separate Supporting Information 12, we use a mono-dimensional model^73,74^ to describe how the complex interplay between the cell-cell and the cell-substrate interaction forces can guide cell assembly into networks. Using mathematical models and computer simulations, we describe the role of the fractal dimension of a substrate in this process.

### Simulating information flow in neuronal networks

We then assessed the ability of neural superclusters to segregate, integrate and direct information. We used computer simulations and mathematical modelling to verify the performance of clustered networks on nanowire surfaces. Starting from fluorescence images of cells on substrates, we realized artificial graphs that reproduce real neural networks. Using generalized leak integrate and fire models^46,74,75^, we simulated the propagation of an initial disturbance throughout those networks. Resulting signal at each node is a train of spikes with characteristic intensity and timing. Temporal distribution of spike patterns encodes the information that the neuron carries about the stimulus. Information theory methods can be used to decode such information^76–78^. For each sequence of spikes, we calculated the associated Shannon entropy $H(\varsigma )=-\,\sum _{S}P(s)lo{g}_{2}P(s)$, where *P*(*s*) denotes the probability with which a stimulus *s* is presented in the response of the node to the disturbance *ς*. *H* quantifies the average amount of information gained with each stimulus presentation and is represented in bits if the logarithm is taken in base 2. Information is the difference of entropies associated to a deterministic (i.e. the signal) and random (i.e. the noise) stimulus, *I* = *S* − *N*.

**Figure 9:**
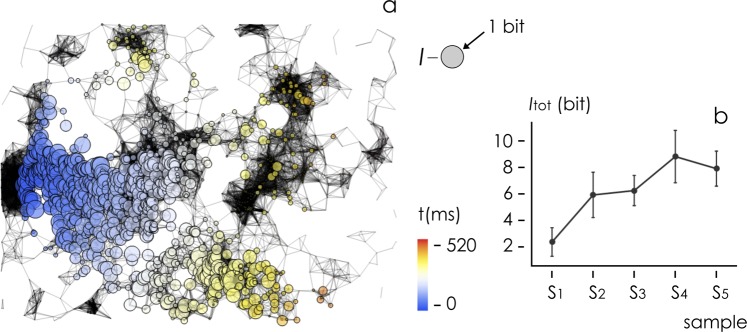
Using generalized leaky and integrate fire models and information theory variables, we determined patterns of information in networks of nerve cells for all considered samples, and herein reported for substrate S_5_ (**a**) as an example. Overall information transmitted in a grid is reported as a function of substrate preparation (**b**).

Figure 9a reports patterns of information simulated in a network cultured on sample S_5_. Because of the characteristics of the network, information propagates far away from the point of application of the initial disturbance, and the intensity of information transported at each node of the network degrades moderately with the distance travelled by the signal. We calculated the total information *I* transmitted in neuronal networks as a function of sample type (Fig. 9b). *I* is determined as the information delivered at a node of the grid integrated over the entire grid and over the whole duration of the process of propagation. We launched more than 100 simulations per substrate. We found that *I* shows a very high sensitivity to the characteristics of the nanowires. *I* is moderately low for substrates with low values of fractal dimension and nanowires density (${I}^{{{\rm{S}}}_{1}} \sim 1.8$ bits), and increases to ${I}^{{S}_{4}} \sim 9.1$ bits and ${I}^{{S}_{5}} \sim 7.9$ bits, in networks cultured on substrates with large fractal dimension and nanowires density. The maximum enhancement factor of information is ${Q}_{E}={I}^{{{\rm{S}}}_{4}}/{I}^{{{\rm{S}}}_{1}}\sim 5.2$. Bonferroni post hot test indicates that information transmitted through networks associated to substrate S_4_ is statistically greater than information transmitted through networks associated to substrates S_1_, S_2_, S_3_, and that information transmitted through networks associated to substrate S_5_ is statistically greater than information transmitted through networks associated to substrates *S*_1_ and *S*_2_ (*p* = 0.05). Remarkably, values of information correlate with the fractal dimension of samples (*r*^2^ = 0.91, *p* = 0.03).

### Measuring spontaneous activity of neuronal networks

**Figure 10:**
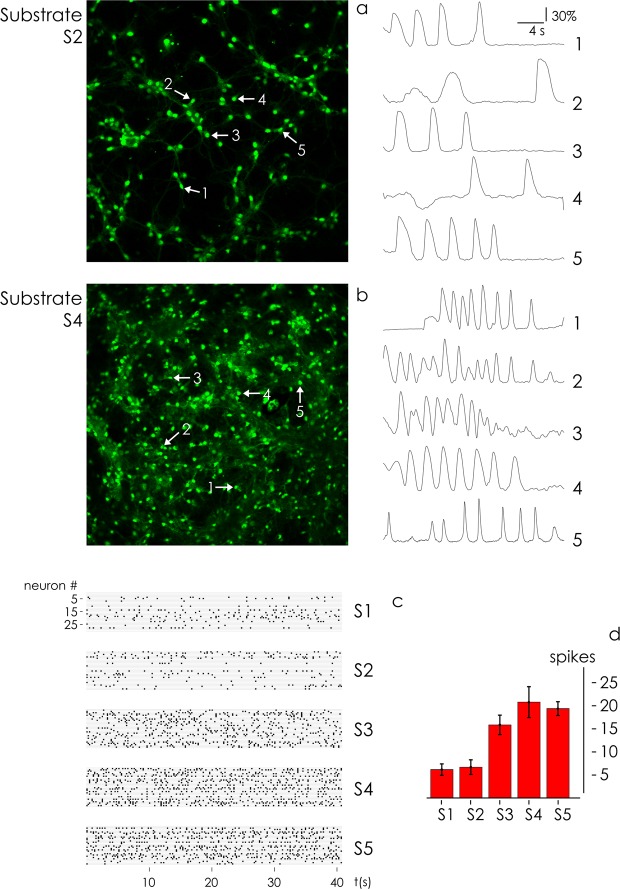
We used fMCI to measure the spontaneous activity of cultured neuronal networks. The transients of calcium in the networks were determined as the fluorescence change ΔF/F in each cell body. Spikes are less dense on substrates with lower nanowires density and fractal dimension (**a**) compared to substrates with larger values of nanowires density and fractal dimension (**b**). The raster plot of neuron activity determined for all substrate preparations (**c**) and the histogram reporting the total number action potentials measured on the same substrates (**d**) indicate that, for higher values of fractal dimension, neuronal networks are increasingly more efficient in the elaboration and transmission of information.

We carried on high speed fMCI (functional multi calcium imaging) experiments to determine the spontaneous activity of neuronal cell networks on different substrate preparations. After sample preparation and incubation with reagents as reported in the methods, we imaged cultures with an upright confocal microscope. We acquired the variation over time of intensity of fluorescence associated to the transients of calcium in the networks, which in turn are indicative of neuronal activity. In each cell body, the fluorescence change ΔF/F was calculated as (F_t_ − F_0_)/F_0_, where F_t_ is the fluorescence intensity at frame time *t*, and F_0_ is a baseline. Spike timings were determined as the onsets of individual Ca^2+^ transients^46,66,67^. Spikes were registered for 40 s and reported in Fig. 10a,b as variation respect to the baseline. For small nanowires density (S_2_) spikes are loosely densely packed in the considered time interval, in contrast to networks cultured on high nanowires density surfaces (S_4_), that generate tightly densely packed trains of signals. This suggests that small world neuronal networks on nanowires surfaces with high values of density and fractal dimension have an increased ability to storage, process, and transmit information – confirming the predictions of the model and the results of the simulations reported in Fig. 9 of the article. Post processing of data enabled to derive the spatio-temporal pattern of spontaneous network activity of 30 neurons randomly selected for fMCI recordings. Spiking events are reported in a matrix plot as a function of time for all considered neurons in a network for each substrate preparation (Fig. 10c). Matrix plot representation of spiking events (action potentials) reveals that neural activity increases with nanowires density. To quantify the level of activity of the networks, we averaged the number of spikes (action potentials) measured over the entire neuron populations for each substrate. The average number of events registered in the networks transitions from $spike{s}^{{S}_{1}}\sim 7\pm 1.4$ spikes/neuron for S1 to $spike{s}^{{S}_{4}}\sim 24\pm 3$ spikes/neuron for S4, with a ~3.5 fold increase, slightly smaller than the maximum enhancement factor of information *Q*_*E*_ ~ 5.2 predicted by computer simulations. Bonferroni post hot test indicates that the number of action potentials generated by the networks on substrate S_4_ and is statistically greater than the number of spikes measured on substrates S_1_ and S_2_ (*p* = 0.05).

For sufficiently high values of fractal dimension and nanowires density, surface topography trigger the formation of small world neuronal networks. Where the high clustering and low path lengths of these types of graphs reinforce communication between neurons and enhance the quantity, quality and density of information. Statistical evidence of the correlation between the small world characteristics of the networks and the information transported through them, may accelerate research in tissue engineering, regenerative medicine, the treatment and diagnosis of neurodegenerative disorders. The aim of the work was not to reproduce an analogue of the brain *in-vitro*, but to explore the mechanisms that lead to the formation of complex neuronal structures, with high levels of correlation within the structures (inter-cluster) and between the structures (intra-cluster), and because of which neurons achieve formidable performance. Zinc oxide nanowires surface represent a system where tight control over the elements of the system allows to analyze simplified variants of the cerebral cortex, where it is possible to determine causal links between variables with precision and reliability.

### Discussion: hypothesis for the formation of the minicolumns in the cerebral cortex

A fundamental feature of the brain of pluricellular organisms is its organization in diverse brain areas and subareas characterized by anatomical and functional subunits. These arise and are shaped during development through a very precise temporal and spatial coordination of cell proliferation, migration and differentiation first, and then by activity-dependent mechanisms^79^. Even when cultured *in vitro*, neurons can self-assemble into complex circuits, showing emerging properties that were absent in the single neurons per se. More precisely, neuronal circuits will be formed by few neuronal clusters with highly interconnected units (i.e., neurons, high *Cc*) separated by short-length paths (small *cpl*). This is a condition typical of small-world networks, which show higher efficiency in mediating information compared to equivalent random networks^13,49,50^.

Previously, we have demonstrated that neurons grown *in vitro* on corrugated surfaces organize in clusters with small-world attributes, minimizing the free-energy of the system, and enhancing the information flow at the same time^46^. Here, we found that an increase in the density of ZnO nanowires is paralleled by an increase of their own fractal dimensions, and leads to the formation of neuronal cluster with an abundance of connections between neighboring neurons. These clusters express hallmarks of small-world networks, resulting in an improved communication among neurons. Intriguingly, from an intermediate density of ZnO nanowires (S_3_) onward, the mean neuronal density of all the observed clusters reached a plateau of ~200 neurons, an arrangement we have named ‘supercluster’. Our finding that clusters with more than ~600 neurons would become unstable and that migration force could further reduce the density limit to a lower value, suggested the possibility that ~200 neuron density value could be an upper limit regarding cluster assembly.

Recently, it has been observed that the minimum number of neurons required to develop and maintain critical network characteristics such as synchronized activity and sufficient coding efficiency in a noisy environment is well below the dimension of a supercluster^80,81^. Thus, an increase in cluster density would result in a larger energy consumption without any functional benefit in terms of information coding. From a developmental standpoint, the initial assemble of neurons would be first limited by the balance between forces of cohesion and migration. Then neuronal cluster formation would be progressively shaped by more complex constraints and their mutual interaction. For example, energy consumption constrains would guide the system toward a reduction in the neuronal number. On the other hand, giving the cluster sufficient redundancy would confer resilience against adverse environmental conditions that may compromise circuit integrity.

In line with this reasoning and our findings, the dimension for a single cluster within different areas of the central nervous system would not exceed a dimension of few hundreds of neurons. The extreme complexity of the mammalian brain and the difficulty to clearly identify elementary functional units by morphological and functional means, makes a thorough experimental evaluation of cluster-density highly challenging. However, some indications might come from the analysis of the so-called cortical minicolums^82^. These structures originate from repeated divisions of a polyclonal set of progenitor cells residing in the ventricular zone^82^. They are constituted by a narrow (20–80 μm diameter) modular arrangement of vertically connected neurons, perpendicular to the pial surface and spanning from layer II to VI^82^. Cortical minicolumns are considered as the elementary unit of the mature neocortex^82^. This concept is reinforced by their ability to clearly switch between ‘on’ and ‘off’ states, which makes them well-suited for memory and pattern processing^83^. Importantly, this switching ability is not always observed among all individual neurons of a minicolumn, so it might be considered as one of the possible properties emerging only upon neuronal clustering. Another important hallmark of minicolumns is the strong prevalence of short connections among their single elements compared to the long-range ones with other columns or brain areas^84^, a condition in line with a high Cc and a small cpl.

Notably, different works estimated the number of neurons forming a single minicolumn in the primate cortex, finding a constant range spanning from 80 to 110^82,85^ (but see the striate cortex with a value 2.5 times larger^82^). Interestingly, the number of neurons per column resulted constant across five diverse species from different mammalian orders^85^. Nevertheless, some research groups questioned the role of mini-columns as the common building block of different cortical areas and the constant cell number among them, even across nine highly related primate species^86–89^. Anyhow, neuronal density was always reported to be below 100 neurons per minicolumn^87^. In conclusion, the supercluster density value of 200 neurons that we have observed in our experiments might represent a physical and energetic limit for any given neuronal clique. However, how this applies in the brain to functional units other than mini-columns is yet to be determined experimentally.

## Methods

### Zinc oxide nanowire synthesis

51MZinc Oxide (ZnO) nanowires have been grown using a two-steps, seed-mediated method previously reported in ref.^55^. At a first stage, a ZnO nanoparticles seed film is deposited onto microscope slide glasses by means of SILAR technique^54^ (Successive Ionic Layer Absorption and Reaction). In the SILAR technique the cationic and anionic constituents of the thin film material are adsorbed on the substrate one at a time and adsorption is mediated by different precursors. Before the second adsorption, sample surface is rinsed with purified water, so that one can achieve layer-by-layer deposition with control at the nanoscale. Such pre-deposited nuclei act as favorable sites for further heterogeneous nucleation/growth of nanowires, accomplished by hydrothermal chemical bath deposition using zinc nitrate and hexa-methyl-entetramine, the latter promoting hydrolysis and anisotropic growth. Commercial available soda lime microscope glasses were used as a substrate for Zinc Oxide (ZnO) Nanowire growth. Glass substrates were cleaned with soap, rinsed in and sonicated in isopropyl-alcohol (IPA) for minutes. Then, they were dried under nitrogen steam. Cleaned microscope glass was immersed in stock aqueous solution of zinc acetate and 1M ammonium hydroxide, with intermediate rinsing by immersion in *d*H_2_O. The process was repeated 30 times, then samples were washed with IPA and dried in vacuum at 120 °C. For ZnO nanowire synthesis, we loaded an hydro-thermal reaction vessel with ml of *d*H_2_O, 20 to 80 mmoles of zinc nitrate hexa-hydrate (Zn(NO_3_) × 6H_2_O), 20 mmoles of hexa-methyl-entetramine (HMTA) and a suitable amount of NaCl (0–80 mmoles). Glass substrates functionalized with ZnO seed layer were placed vertically into the reactor, sealed and placed in a thermostatic oven at 110 °C for 16 h. Samples were then rinsed with *d*H_2_O, IPA and dried under nitrogen. Varying the proportion of reagents solution, we obtained nanowires with different density on the substrate. Conditions for sample synthesis and resulting sample characteristics are recapitulated in a separate Supporting Information 1. All reagents were purchased from VWR International and used without further purification: zinc nitrate hexa-hydrate (Zn(NO_3_) × 6H_2_O, 99%), hexa-methyl-entetramine (HMTA, (CH_2_)_6_N_4_, 99%), sodium chloride (NaCl, 99%).

### SEM imaging of nanowire surfaces

SEM images of the samples were captured using a Dual Beam (SEM-FIB) - FEI Nova 600 NanoLab system. During the acquisitions beam energies of and 15 keV, and corresponding electron currents of 0.98 pA and 0.14 nA, were used. In some cases the mode 2 configuration was set, through which images can be magnified over 2.5 × 10^6^ to achieve ultrahigh resolution.

### AFM Imaging and mechanical characterization of nanowire surfaces

We used for the analysis of samples an ICON AFM from Bruker, with 30 pm noise, drift rates less than 200 pm per minute and sub-nanometer resolution in *z*. Ultra-sharp probes were used for ultra-high resolution with a radius of curvature at the tip as low as *R*_*c*_ = 5 *nm*, assuring an estimated maximum lateral resolution ${R}_{l}=\sqrt{0.8\,{R}_{c}}\sim 2\,nm$. Images were acquired in tapping mode in air setting a frequency of 0.5 Hz and a resolution of 512 × 512 pixels. Several measurements were performed for each sample setting scanning areas of 2 μm × 2 μm, 5 μm × 5 μm, 10 μm × 10 μm. Bi- and three- dimensional image elaboration was conducted using the version 1.40 of Nanoscope software (from Bruker). Force measurements and mechanical characterization of samples were performed using a scan size of 5 μm, with a ramp size of 670.0 nm and a ramp rate of 1 Hz, using an antimony n-doped silicon cantilever with spring constant of 42 N/m, (from TESPA).

### Deriving the fractal dimension of nanowire surfaces

AFM profiles of the surfaces were processed using the algorithms developed and described in ref.^56^. For each surface, we derived the characteristic power spectrum (PS) density function (Fig. 1c). The PS represents the information content of a surface as a function of scale. In a log-log plot, the PS density function appears as a line with a slope β. Since the fractal dimension *D*_*f*_ is a measure of change of surface detail for change of scale, *D*_*f*_ of a surface can be determined from β as^56^ *D*_*f*_ = (8 − β)/2. The fractal dimension *D*_*f*_ of a surface ranges from 2 (Euclidean dimension of a flat surface) to 3 (representing an extremely rough surface).

### Animals and cell culture

All animal experiments were approved by the local committee OPBA (Organism Proposed for Animal Welfare). All methods were performed in accordance with the relevant guidelines and regulations. Hippocampal neural stem/progenitor cells, NSPCs, were isolated from isolated from the embryonic Wistar rat cerebral cortex, on day 14–15, as previously described (R.Y. Tsai, R.D. McKay Cell contact regulates fate choice by cortical stem cells J Neurosci, 20 (2000), p. 3725). Embryonic rat cerebral cortices were mechanically triturated in cold PBS. Resulting suspension was successively filtered through 100, 40, 25–*μm* mesh cell strainers. Dissociated cells were collected by centrifugation and resuspended in Primary Neuron Basal Medium (PNBM), SingleQuots (Lonza), an fully supplemented with 2 mM L-Glutamine, 50μg/ml Gentamicin, 37 ng/ml Amphotericin and fresh 2% NSF-1. Number of live cells was counted by Trypan blue exclusion assay in a hemocytometer. Cerebral cortical neuron cells were cultured in T25 culture flasks (Corning) at a density of 50000 cells/cm^2^ in the above culture medium and maintained at 37 °C in a humidified atmosphere of 95% air and 5% CO_2_. After 48 hours from culture, suspended cells underwent cell division. Cell division continued for an additional 2 days. Cells were then seeded on poly-D-lysine with Laminin coated substrates. We coated substrates with Poly–D–Lysine and Laminin following the protocols reported in www.lonza.com.

### Neuronal cells staining

For morphologic characterization, cultured cells were fixed in ice-cold 4% paraformaldehyde in PBS for 25 min and incubated with 0.3% Triton X–100 for 10 min. After fixing, cells were incubated with DAPI (Invitrogen) and Alexa Fluor 488 Phalloidin (Invitrogen) diluted in PBS containing 10% bovine serum albumin overnight at 4 °C. Fluorescent phalloidin was choice as a marker for F-actin and DAPI for the nuclei of the cells.

### Imaging cultured neuronal cells

An inverted Leica TCS-SP2 laser scanning confocal microscopy system was used to image cells adhering on the substrates. All measurements were performed using a ArUv laser. The pinhole (~82 μm) and laser power (80% power) were maintained throughout each experiment. Confocal images of blue (DAPI) fluorescence were collected using a 405 nm excitation line and a 10× dry objective, so that cells with a characteristic size of a few microns could be clearly observed. Confocal images of green (Phalloidin) fluorescence were collected using a a 488 nm excitation line. For each substrate, a large number of images was taken for statistical analysis. Each image was acquired over a region of interest of 882 × 882 μm^2^ and averaged over 4 lines and 10 frames to improve quality and reduce noise.

### Cytometry phenotype characterization

To confirm neuronal differentiation, cultured adherent cells were dissociated and fixed in ice-cold 0.3% glutaraldehyde and stained with primary antibodies. Primary mouse anti-Nestin monoclonal antibody (Alexa Fluor 647 BD), mouse anti-Glial Fibrillary Acidic Protein polyclonal antibody (PE anti GFAP, BD) and mouse anti-β-Tubulin, class III (AlexaFuor 488 BD) were used. Cells were labelled according to the protocols specified by the manufacturer of the antibodies. Cells were analyzed using a Sorter ARIA III BD flow cytometer and the data was analyzed with FACSDiva v. 6.1.3 BD Becton Dickinson. Flow cytometry analysis confirmed that the cells *in vitro* grow homogeneously expressing neural differentiation markers as Nestin and GFAP with a media percentage of 59% and 75% of cultivated cells respectively. Average expression of β-Tubulin class III was of 25%.

### Simulating information flows in neuronal networks

We used a generalized leaky integrate and fire model^90,91^ to simulate information flow in bi-dimensional neural networks as described in refs^46,74,75^. The temporal sequence of spikes that propagate along the grid encodes the information transmitted over that grid. Resulting patterns of multiple spike trains were interpreted using information theory approaches^76–78^. We represented the variability of individual neurons in response to a long random stimuli sample with the total entropy *H*. Similarly, the noise entropy *N* is the variability of the spike train in response to a sample of repeated stimuli. The information content provided by the different spike trains is the difference between entropies *I* = *H* − *N*. Connection or synaptic weights are unitary in the model - the quality of *strength* of a connection between nodes does not depend on the position of those nodes in the grid and their distance, there are no preferential routes of information spread in the grid nor low-noise channels. Given two nodes A and B in the grid, information can propagate from A to B in complex paths. The quality of information in a grid would depend on the *architecture* of the network and on the fact that some topological structures are more efficient than others. However, even if weights are unitary everywhere in a grid, in the model the stimulus on a neuron is written as $I(t)={\sum }_{i}^{\beta }\,\zeta ({d}_{ij})\,{J}_{i}\,{\sum }_{k}^{rel}\,\delta (t-{t}_{i}^{k})$, where *k*:1 − *rel* represents the temporal pattern of neurotransmitter release events, *δ* is the Dirac delta, *J* is the characteristic current of a single neuron, *β* are the mutually connected neurons in a cluster. *ζ*(*d*_*ij*_) is damping term, it accounts for the fact that the signal can *degrade* when it travels long distances *d*_*ij*_. Notice that, in this form, the model does not achieve reinforcement learning. We neglect synaptic plasticity, i.e. the change in strength of connections between neurons. In a more sophisticated evolution of the model that will be developed over time, we will consider adaptive networks – i.e. dynamic networks where the configuration of a network and its connection may change with time to maximize a cumulative reward.

### Functional multi calcium imaging (fMCI)

To measure the activity of neurons on the nanowires substrates we used the methods described in ref.^75^.

## Supplementary information

Supporting Information

## Data Availability

The data that support the findings of this study are available from the corresponding authors upon request.
