# Publisher Correction: Cortical-like mini-columns of neuronal cells on zinc oxide nanowire surfaces

**DOI:** 10.1038/s41598-020-67584-4

**Published:** 2020-07-03

**Authors:** V. Onesto, M. Villani, R. Narducci, N. Malara, A. Imbrogno, M. Allione, N. Costa, N. Coppedè, A. Zappettini, C. V. Cannistraci, L. Cancedda, F. Amato, Enzo Di Fabrizio, F. Gentile

**Affiliations:** 1 Center for Advanced Biomaterials for HealthCare, Istituto Italiano di Tecnologia, 80125 Naples, Italy; 2 Department of Experimental and Clinical Medicine, University of Magna Graecia, 88100 Catanzaro, Italy; 3 IMEM-CNR, Parco Area delle Scienze 37/A, 43124 Parma, Italy; 4 Istituto Italiano di Tecnologia, Via Morego 30, 16163 Genova, Italy; 5 Tyndall National Institute, Cork, T12 R5CP Ireland; 6 PSE Division, King Abdullah University of Science and Technology, Thuwal, 23955−6900 Saudi Arabia; 7 Health Department, University of Magna Graecia, 88100 Catanzaro, Italy; 8 Biomedical Cybernetics Group, Biotechnology Center (BIOTEC), Center for Molecular and Cellular Bioengineering (CMCB), Center for Systems Biology Dresden (CSBD), Department of Physics, Technische Universität Dresden, Tatzberg 47/49, 01307 Dresden, Germany; 9 Brain Bio-Inspired Computing (BBC) Lab, IRCCS Centro Neurolesi “Bonino Pulejo”, 98124 Messina, Italy; 10 Dulbecco Telethon Institute, Rome, Italy; 11 Department of Electrical Engineering and Information Technology, University Federico II, Naples, Italy

Correction to: *Scientific Reports* https://doi.org/10.1038/s41598-019-40548-z, published online 11 March 2019

The original version of this Article contained a typographical error in the spelling of the author Enzo Di Fabrizio, which was incorrectly given as Enzo DI Fabrizio. This has now been corrected in the PDF and HTML versions of the Article.

