# Supplementary material for: Cortical-like mini-columns of neuronal cells on zinc oxide nanowire surfaces
Source: Sci Rep. 2019 Mar 11;9:4021. doi: 10.1038/s41598-019-40548-z (PMC6411964; doi:10.1038/s41598-019-40548-z)
Supplement: Supplementary file 1 — Supporting Information [file 41598_2019_40548_MOESM1_ESM.docx]

**Cortical-like mini-columns of neuronal cells on zinc oxide nanowire surfaces**

V. Onesto^1,2^, M. Villani^3^, R. Narducci^4^, N. Malara^2^, A. Imbrogno^5^, M. Allione^6^, N. Costa^7^, N. Coppedè^3^, A. Zappettini^3^, C.V. Cannistraci^8,9^, L. Cancedda^4,10^, F. Amato^11^, E. Di Fabrizio^6^, F. Gentile^11,*^

^1^ Center for Advanced Biomaterials for HealthCare, Istituto Italiano di Tecnologia, 80125 , Naples, Italy

^2^ Department of Experimental and Clinical Medicine, University of Magna Graecia, 88100 Catanzaro, Italy

^3^ IMEM-CNR Parco Area delle Scienze 37/A - 43124 Parma, Italy

^4^ Istituto Italiano di Tecnologia, Via Morego 30, 16163 Genova, Italy

^5^ Tyndall National Institute, Cork, T12 R5CP, Ireland

^6^ PSE division, King Abdullah University of Science and Technology, Thuwal 23955 − 6900, Saudi Arabia

^7^ Health Department, University of Magna Graecia, 88100 Catanzaro, Italy

^8^ Biomedical Cybernetics Group, Biotechnology Center (BIOTEC), Center for Molecular and Cellular Bioengineering (CMCB), Center for Systems Biology Dresden (CSBD), Department of Physics, Technische Universität Dresden, Tatzberg 47/49, 01307 Dresden, Germany

^9^ Brain Bio-Inspired Computing (BBC) Lab, IRCCS Centro Neurolesi “Bonino Pulejo”, Messina, 98124, Italy

^10^ Dulbecco Telethon Institute, Italy

^11^ Department of Electrical Engineering and Information Technology, University Federico II, Naples, Italy

**Supporting information**

| **SI 1** | *Process parameters for Zinc Oxide Nanowires growth and resulting samples characteristics* |  |
| --- | --- | --- |
| **SI 2** | *Additional SEM and AFM images of Zinc Oxide Nanowires* |  |
| **SI 3** | *Determining the value of nanowire density from SEM images* |  |
| **SI 4** | *Extended topological measures of networks and optimal choice of Waxman variables* |  |
| **SI 5** | *Additional fluorescence images of neuronal networks on Zinc Oxide Nanowires* |  |
| **SI 6** | *Evaluating the level of power-law-ness of the distribution of the degree of the graphs* $k$ |  |
| **SI 7** | *Imaging neurites in cultured neural networks* |  |
| **SI 8** | *Imaging neurite branching in cultured neuronal networks* |  |
| **SI 9** | *Cluster analysis of neuronal networks on nanowire surfaces* |  |
| **SI 10** | *Non linear fit and confidence levels of neuronal cell densities* |  |
| **SI 11** | *Free energy landscape of neural clusters and maximum allowed cluster size* |  |
| **SI 12** | *The physical mechanisms of cluster formation* |  |

**Supporting Information 1***. Process parameters for Zinc Oxide Nanowires growth and resulting samples characteristics*.

Using the methods reported in the main article and references^1-4^, we produced $5$ different samples. Process parameters utilized for each sample are reported in the Supplementary Table 1.1.

| **Sample** | **Zn(NO_3_)_2_×6H_2_O** | **HMTA** | **NaCl** |
| --- | --- | --- | --- |
| S1 | 20 mmol | 20 mmol | - |
| S2 | 40 mmol | 20 mmol | - |
| S3 | 80 mmol | 20 mmol | - |
| S4 | 80 mmol | 20 mmol | 20 mmol |
| S5 | 80 mmol | 20 mmol | 80 mmol |

**Supplementary Table 1.1**

The growth mechanisms of Zinc Oxide ($\mathrm{ZnO}$) nanostructures have been widely investigated^4^. It is universally accepted that the fastest growth rate occurs along the $[0001]$ direction, possibly enhanced by the use of $\mathrm{HMTA}$ which coordinates and bridges two $Zn^{2+}$ onto $[01\underline{1}0]$ planes, acting as a bidentate Lewis base. By increasing the $\mathrm{HMTA}$ content (samples $S_{1}-S_{3}$) with respect to zinc cations ($1:1$ to $0.25:1$), the lateral growth of ($\mathrm{ZnO}$) nanowires is slowed down resulting in the higher aspect ratio of sample $S_{1}$ compared to $S_{2}$ and $S_{3}$. The overall reaction describing the growth stage of $\mathrm{ZnO}$ nanowires can be summarized as follows: (1) the zinc salt undergoes dissociation in water; (2-3) the $\mathrm{HMTA}$ dissociation promotes alkaline hydrolysis of zinc cations (4) which form nanostructures in form of insoluble hydroxide clusters, which finally dehydrate into $\mathrm{ZnO}$ (5).

| $\mathrm{Zn}{(NO_{3})}_{2}\to Zn^{2+}+2NO_{3}^{-}$ | (SI 1.1) |
| --- | --- |
| ${(CH_{2})}_{6}N_{4}\to6HCHO+4\mathrm{NH}_{3}$ | (SI 1.2) |
| $\mathrm{NH}_{3}+H_{2}O\to2NH_{4}^{+}+OH^{-}$ | (SI 1.3) |
| $\mathrm{Zn}^{2+}+2 OH^{-}\to\mathrm{Zn}{(OH)}_{2}$ | (SI 1.4) |
| $\mathrm{Zn}{(OH)}_{2}\to ZnO+H_{2}O$ | (SI 1.5) |

A remarkable change in $\mathrm{ZnO}$ nanowire morphology is observed by adding chloride anions to the solution; under such conditions, a polarity-controlled growth is achieved. Chloride anions are known to bound electrostatically along the $[0001]$ direction lowering the interfacial energy of the most reactive $(0002)$ plane at a point where it is comparable with that of the six equivalent $[01\underline{1}0]$ planes, resulting in the formation of nano-disks instead of nanowires ($S_{4}-S_{5}$).

**Supporting Information 2***. Additional SEM and AFM images of Zinc Oxide Nanowires*.

We present additional SEM and AFM images of samples in support to the images presented in the main article. Tilted SEM images of samples are reported in the **Supporting Information Figures 2.1** (bar in the insets indicate $1 \mu m$) for all substrate preparations $S_{1}-S_{5}$. Top-view and cross sectional images of sample are reported in the in the **Supporting Information Figures 2.2-3**. Large format, high-resolution AFM images of samples are reported in the **Supporting Information Figures 2.3-8** for all sample types.


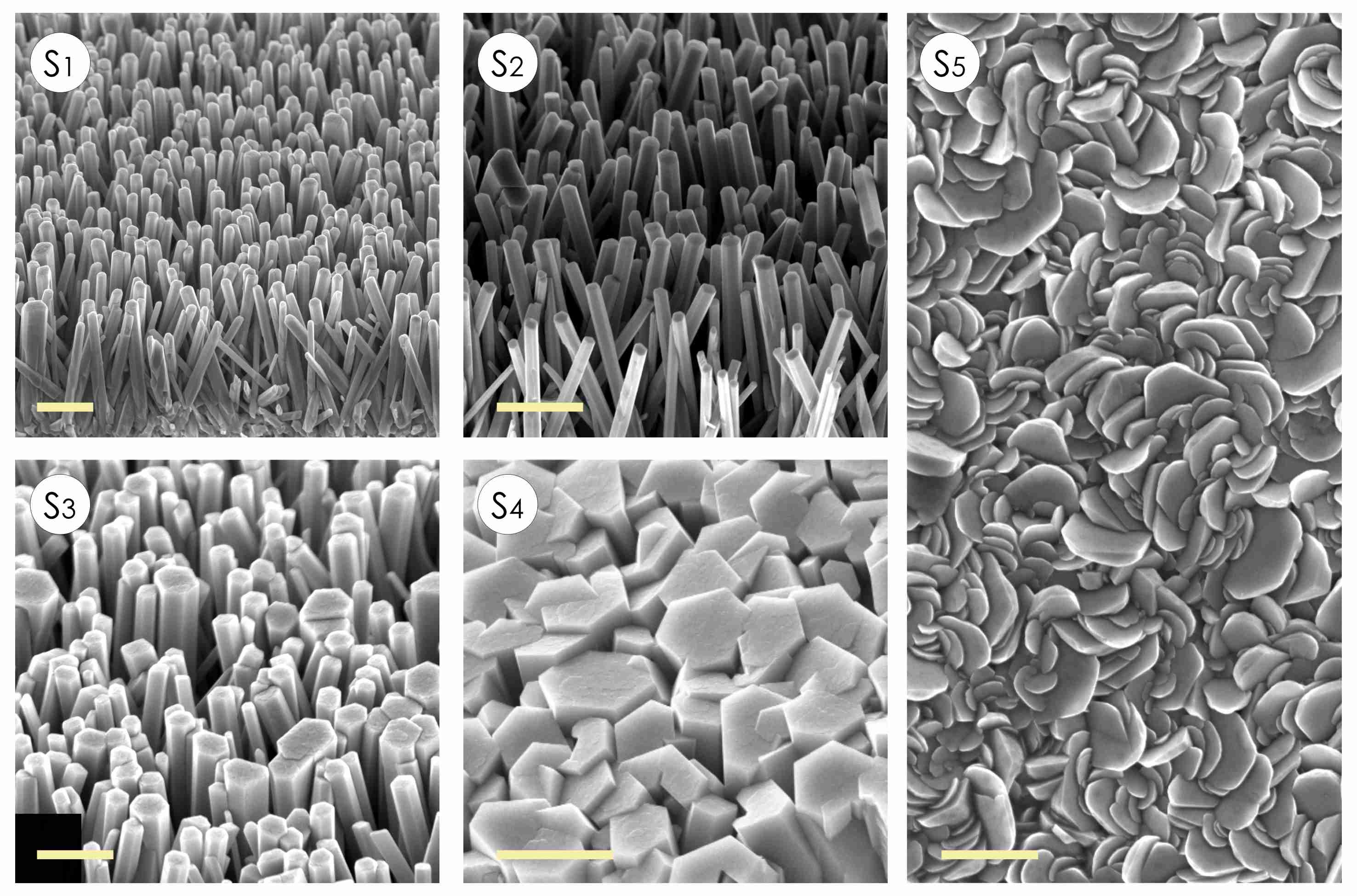


**Supporting Information Figure 2.1** Tilted images of samples for different substrate preparations ($S_{1}-S_{5}$).

**
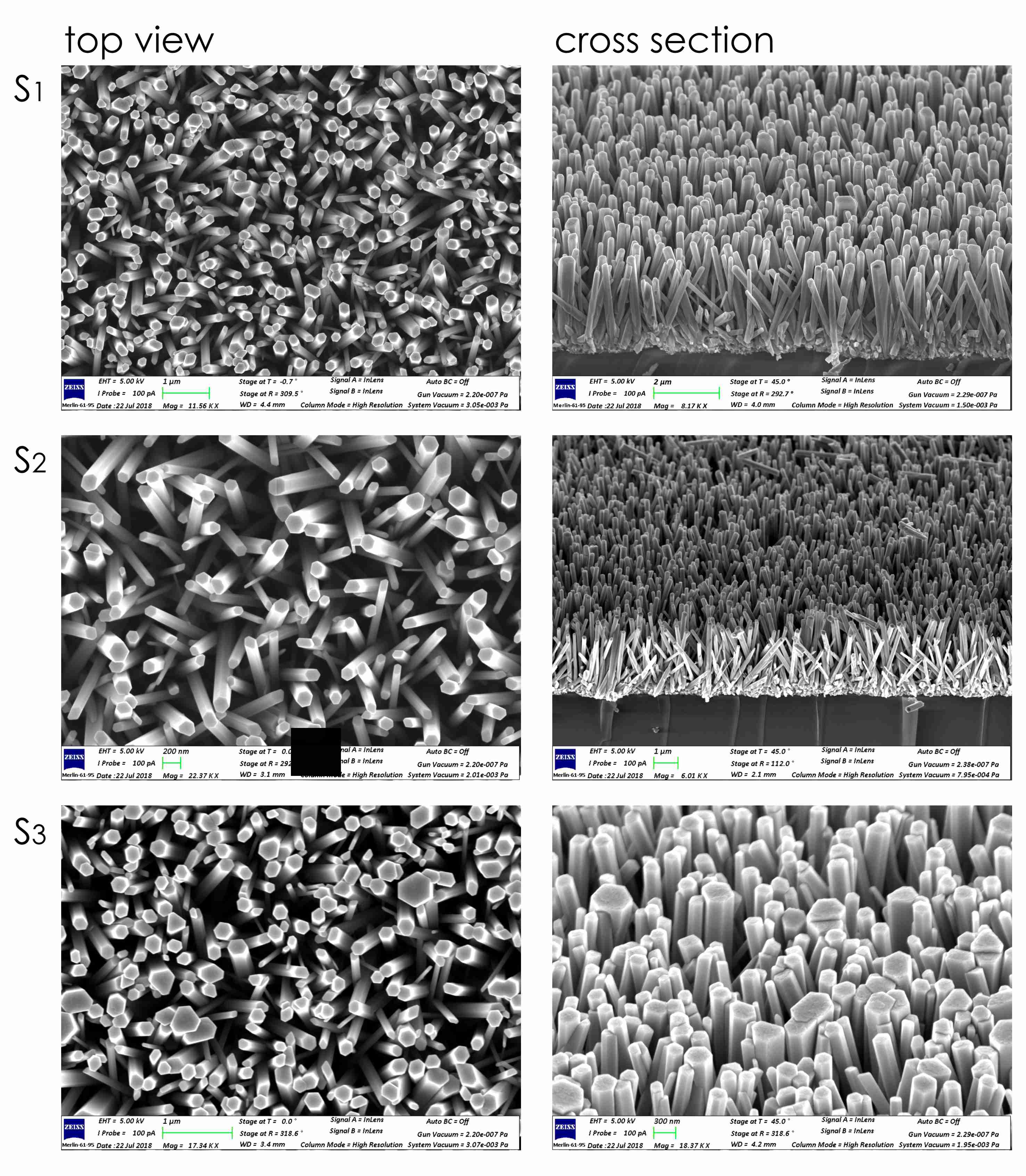
**

**Supporting Information Figure 2.2** Top-view and cross sectional images of ($S_{1}-S_{3}$).

**
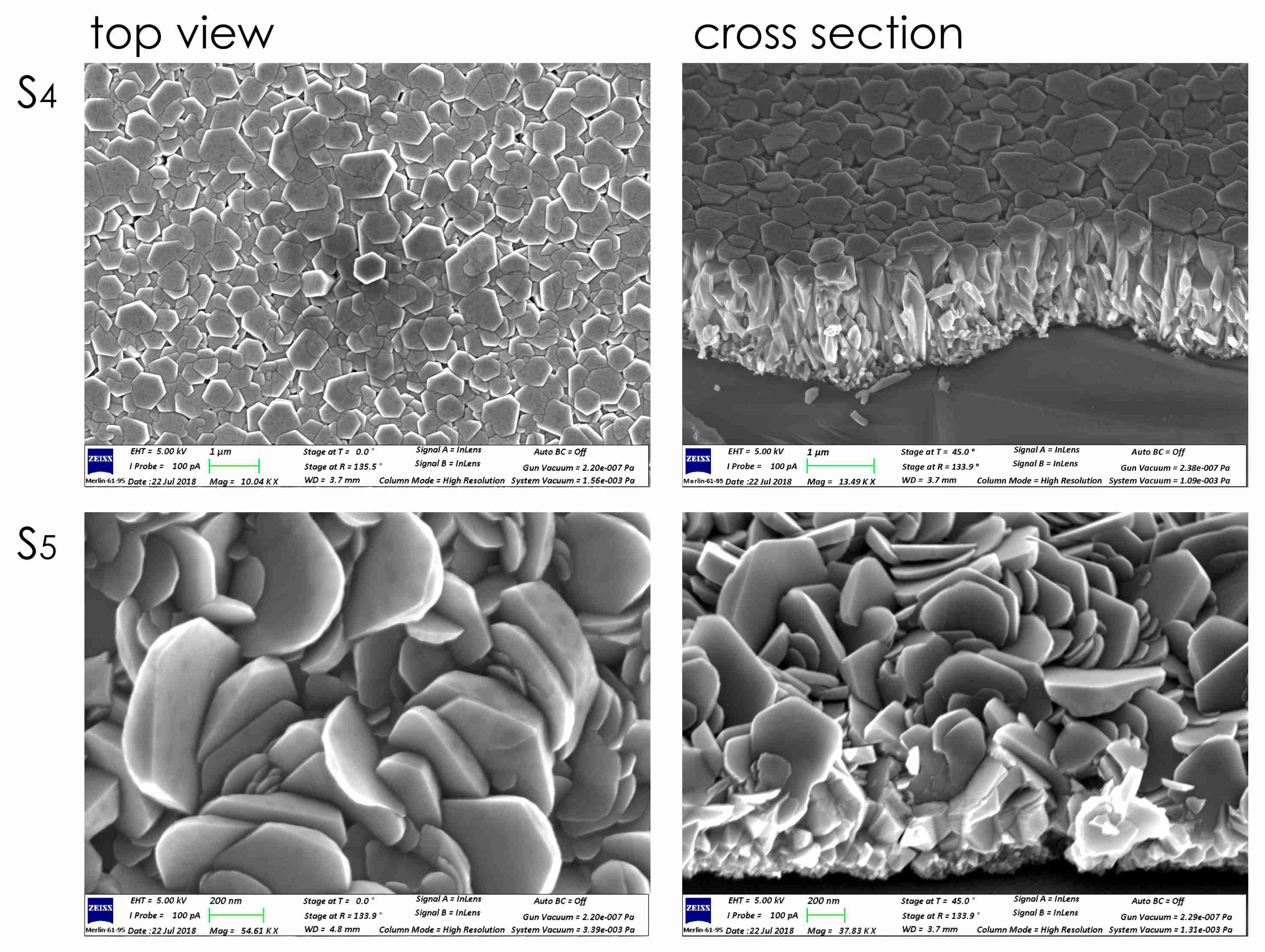
**

**Supporting Information Figure 2.3** Top-view and cross sectional images of ($S_{4}-S_{5}$).


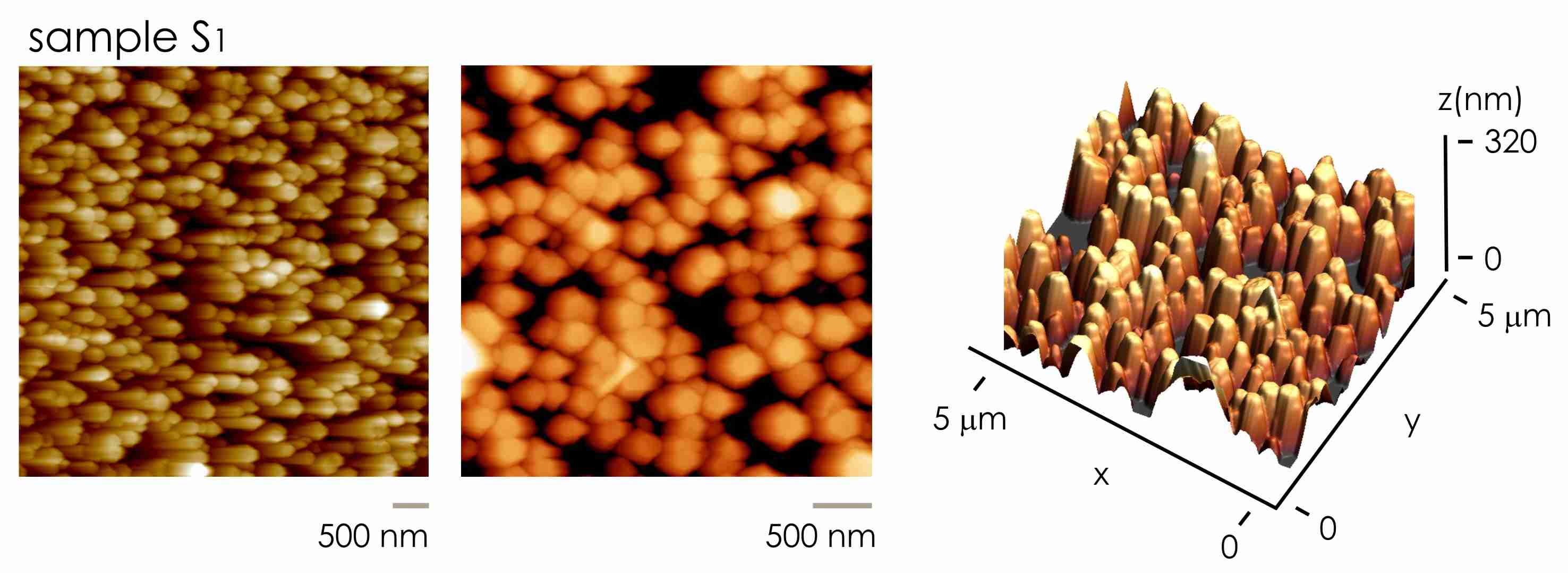


**Supporting Information Figure 2.4** AFM images of sample $S_{1}$.


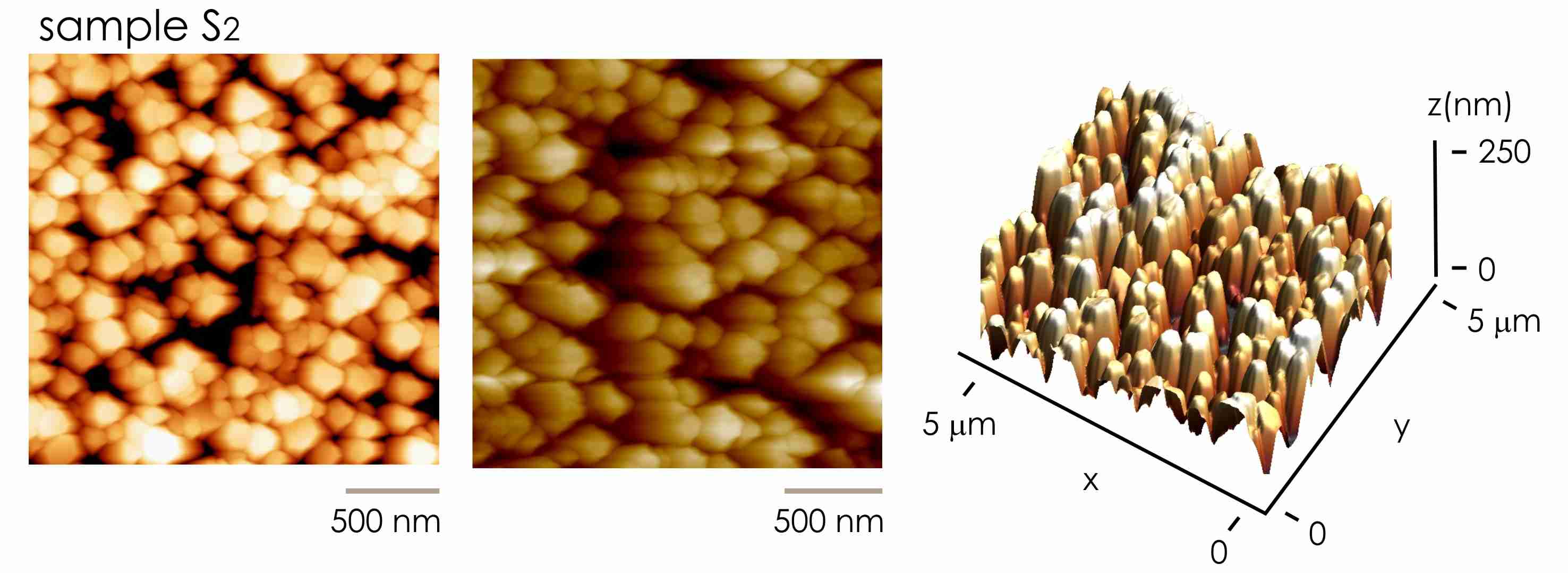


**Supporting Information Figure 2.5** AFM images of sample $S_{2}$.


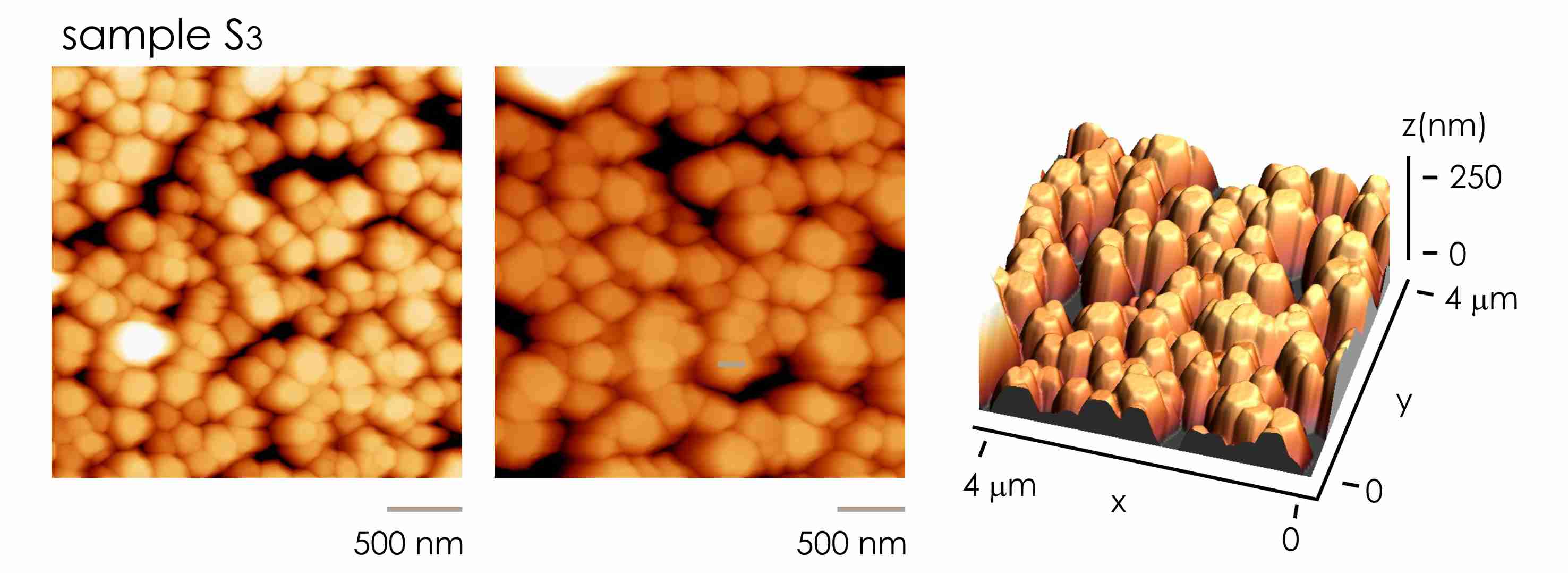


**Supporting Information Figure 2.6** AFM images of sample $S_{3}$.


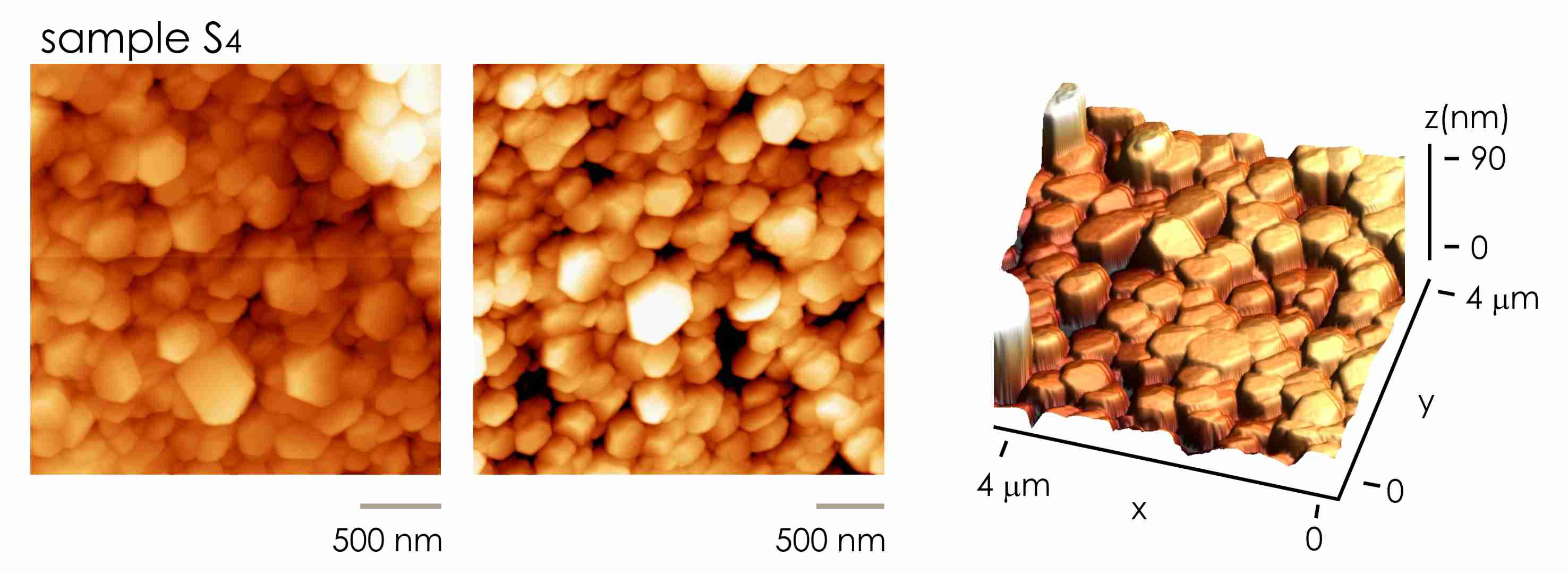


**Supporting Information Figure 2.7** AFM images of sample $S_{4}$.


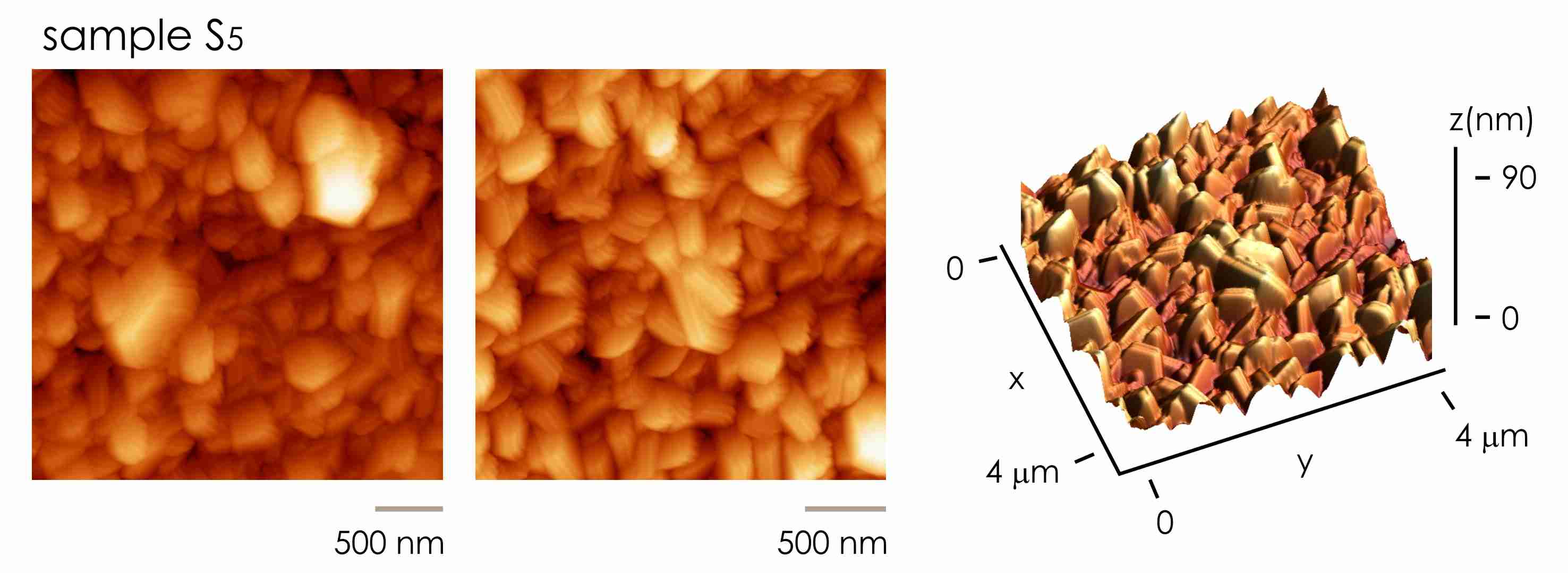


**Supporting Information Figure 2.8** AFM images of sample $S_{5}$.

**Supporting Information 3***. Determining the values of nanowire density from SEM images.*

Given sets of nanowire SEM images, we segmented them in Matlab^®^ to obtain their surface fraction and density. We have chosen a method consisting of some basic morphological operations, for its simplicity and ease of implementation, considering the variability in magnification and in rods pattern between one image and another. To extract the nanowires, it is firstly necessary to perform some local contrast adjustments. In particular, we enhanced the contrast of the starting SEM grayscale image (**Supporting Information Figure 3.1a**) by transforming the values using contrast-limited adaptive histogram equalization (*CLAHE*)^5^. *CLAHE* operates on small regions in the image, called tiles*,* rather than the entire image and enhances the contrast of these areas by adjusting their local histograms, so that the histogram of each output region approximately matches the specified histogram (uniform distribution in this case, which means that all the intensity ranges have equivalent probabilities) (**Supporting Information Figure 3.1d**)**.** The neighboring tiles are then combined using bilinear interpolation to eliminate artificially induced boundaries. Even though *CLAHE* transforms local histograms to the local probability, it does not modify the global histogram much. In addition, it does not cause some of the intensities to be "extra bright" like a global histogram equalization. While on a global level it does not improve the contrast of large objects much, it does indeed make the more subtle (smaller) features in the image more pronounced (**Supporting Information Figure 3.1b**). For example, notice the contours around the rods. This transformation makes them more suitable for foreground detection of subtle features. On the other hand, it also enhances noise, especially if the tiles of the adaptive histogram are very small.

**
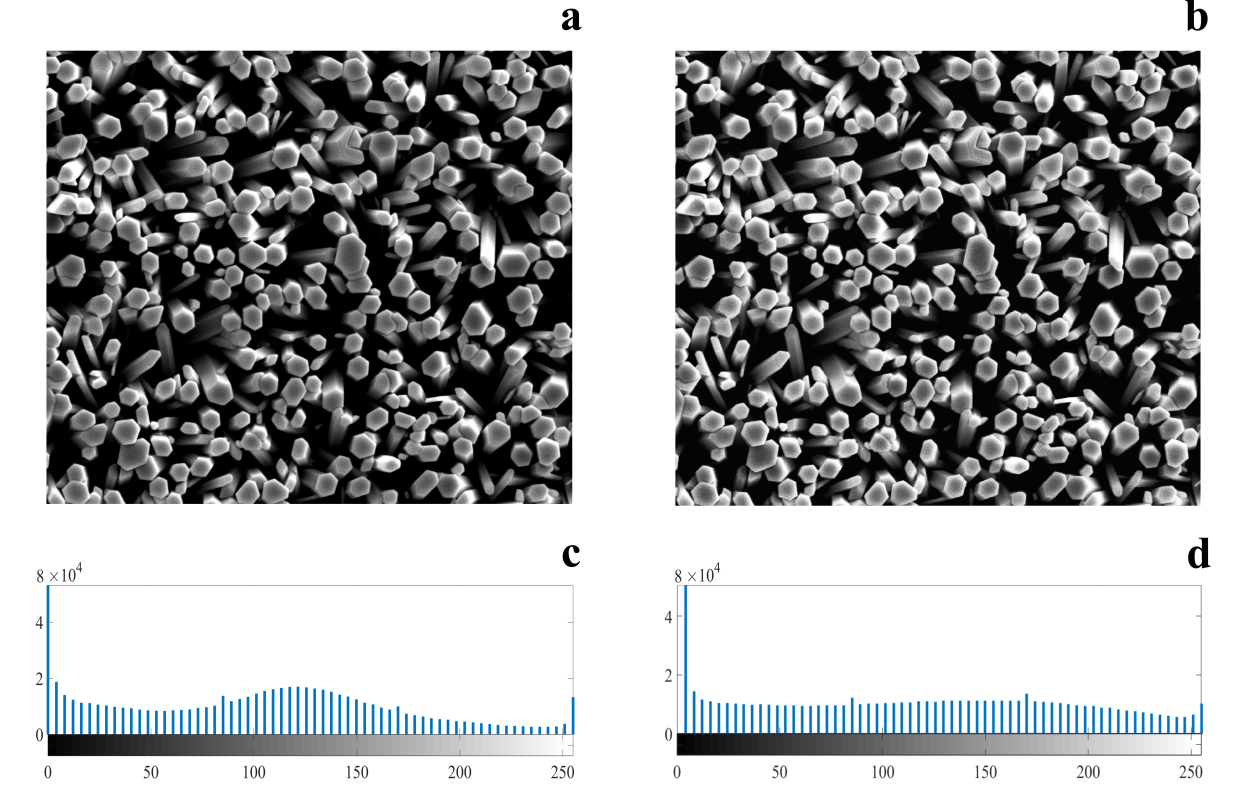
**

**Supporting Information Figure 3.1** (a) Original SEM image and its histogram (c). (b) Image after CLAHE contrast adjustment and relative histogram (d).

After the local contrast adjustments, we converted the image to binary (**Supporting Information Figure 3.2a**). The output image replaces all pixels in the input image with luminance greater than a threshold level (in the range [0,1]) with the value 1 (white) and replaces all other pixels with the value 0 (black). The threshold is found using Otsu's method^6^, which chooses the threshold to minimize the intraclass variance of the black and white pixels. Because of the great morphological and contrast variance in our sets of images, the use of an automatic global threshold for binarization (Otsu's method) caused the algorithm to perform poorly on some images. In these cases, manually adjusting the threshold resulted in much better detection of the bright spots. Because nanowires have varying contrast within themselves, a simple binarization leaves holes in the image. Therefore, we performed a flood-filling operation on background pixels by the *imfill* function (**Supporting Information Figure 3.2b**).


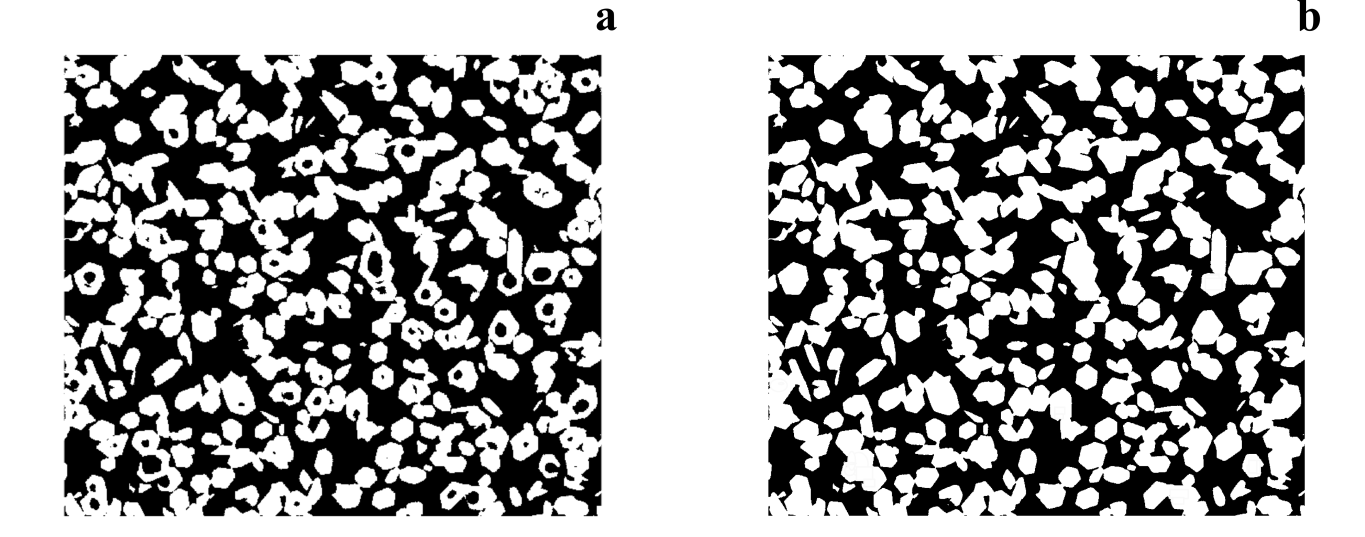


**Supporting Information Figure 3.2** (a) Binary image before and after (b) filling holes operation.

Once obtained the final binarized image, with white pixel representing the cross section (the foreground) of the rods and the black pixel the background, we estimated the Solid Fraction. It expresses the packing grade of the structures. The SF is simply calculated as the number of white pixels $N_{w}$compared to the total pixels $N_{w}+N_{b}$ of the image:

| $SF=\frac{N_{w}}{N_{w}+N_{b}}$ | (SI 3.1) |
| --- | --- |

The method was applied to (i) top-view and (ii) cross-sectional (**Supporting Information Figure 3.3-5**) SEM images of samples. From the top-view SEM images, we derived the Solid Fraction for each sample type, reported in **Figure 3b** of the main text. From the cross-sectional images, we derived the internal Density of the nanowires reported in the **Supporting Information Table 3.1**. Values of Solid Fraction match with values of internal Density with a good level of accuracy. Thus we assume that Solid Fraction obtained from top-view images of samples is a good estimate of sample density.

| *Sample type* | *Solid Fraction* | *Density* |
| --- | --- | --- |
| $S_{1}$ | $0.34\pm0.053$ | $0.37\pm0.083$ |
| $S_{2}$ | $0.426\pm0.051$ | $0.459\pm0.071$ |
| $S_{3}$ | $0.48\pm0.066$ | $0.51\pm0.095$ |
| $S_{4}$ | $0.90\pm0.070$ | $0.92\pm0.09$ |
| $S_{5}$ | $0.99\pm0.045$ | $0.98\pm0.055$ |

**Supporting Information Table 3.1** Values of solid fraction and density of nanowires surfaces extracted by (i) top-view and (ii) cross-sectional SEM images of samples.


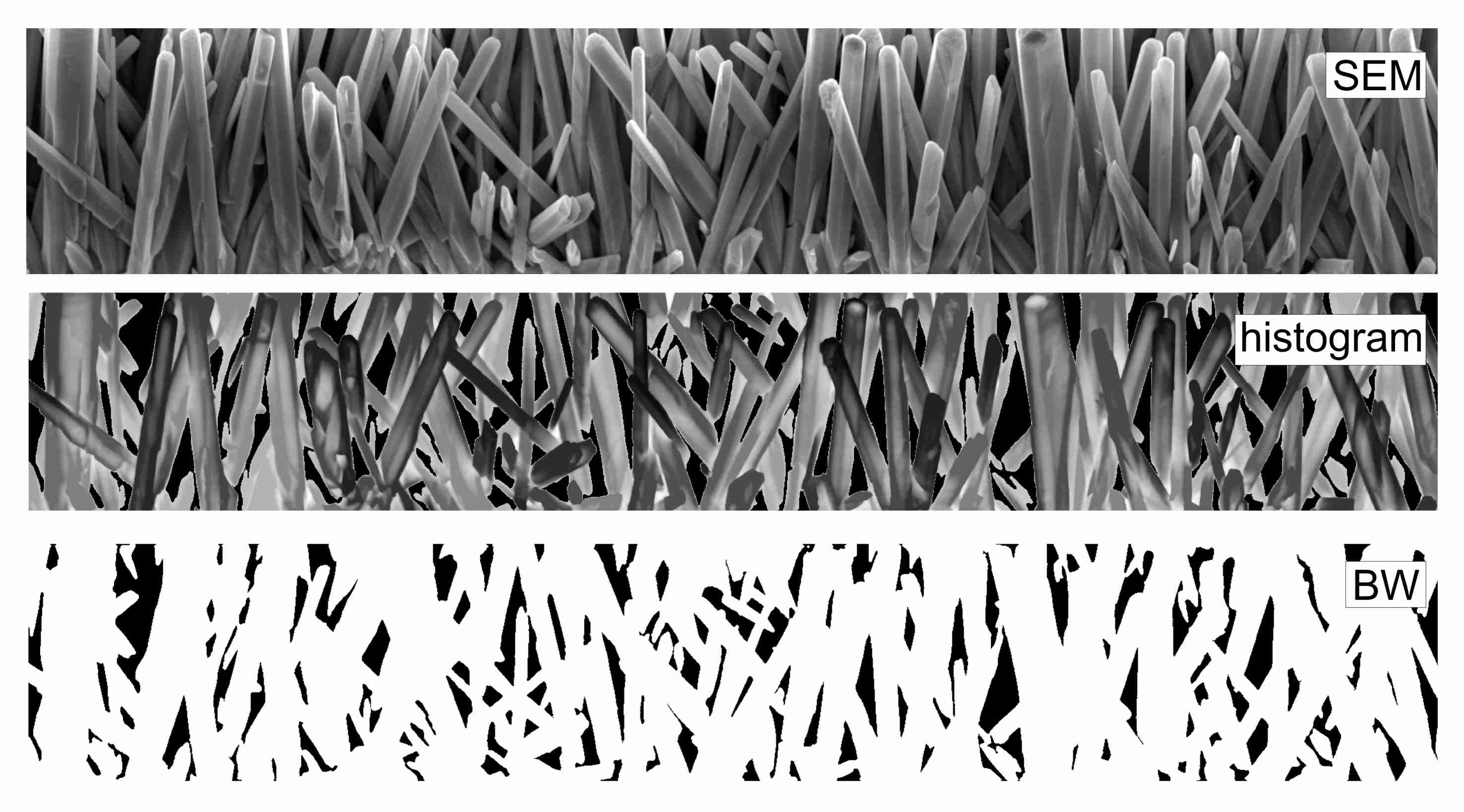


**Supporting Information Figure 3.3** Morphological operations on SEM micrographs. Original cross sectional image of sample, histogram representation of sample, and binary image of sample (sample $S_{1}$).


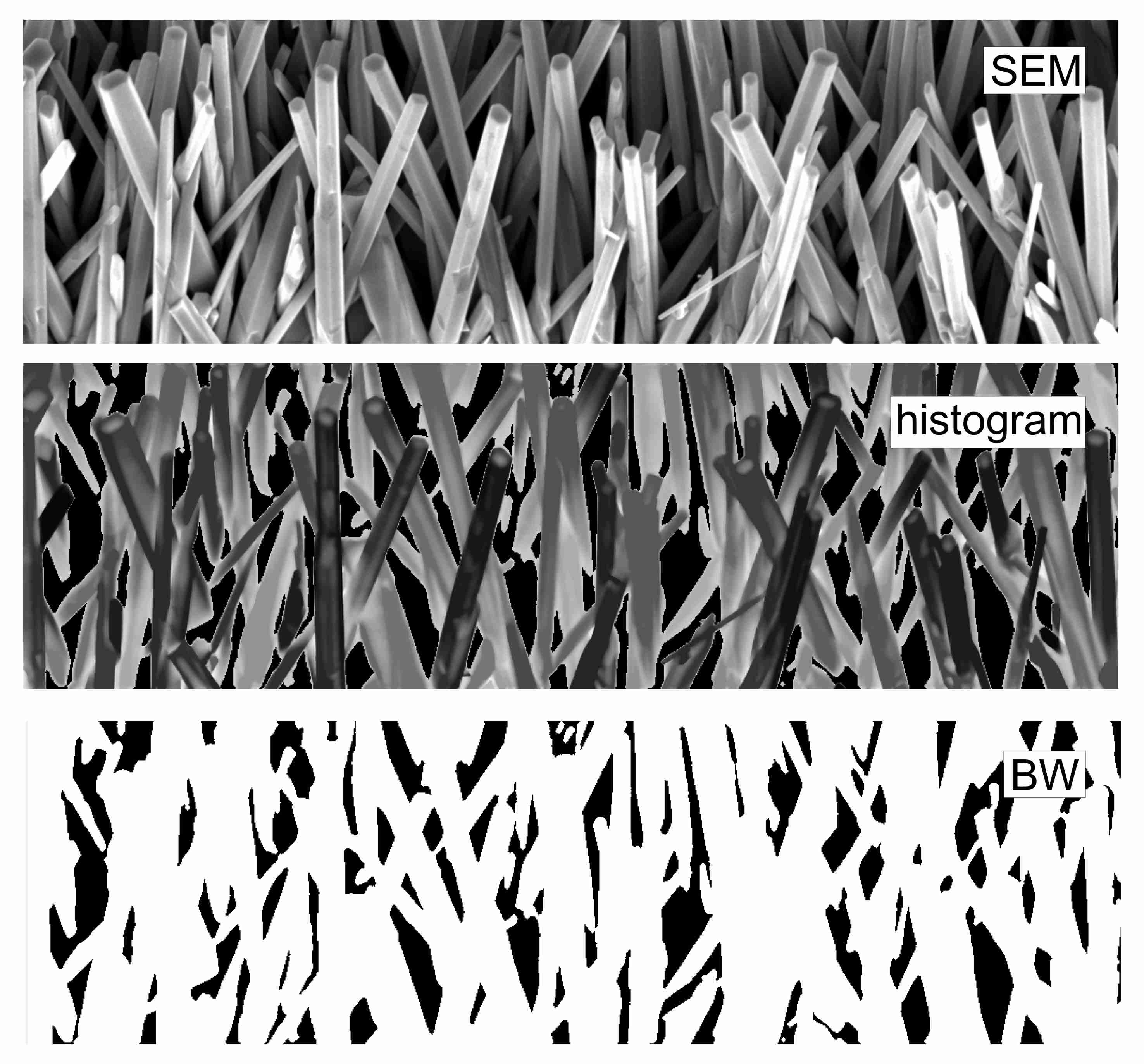


**Supporting Information Figure 3.4** Morphological operations on SEM micrographs. Original cross sectional image of sample, histogram representation of sample, and binary image of sample (sample $S_{2}$).


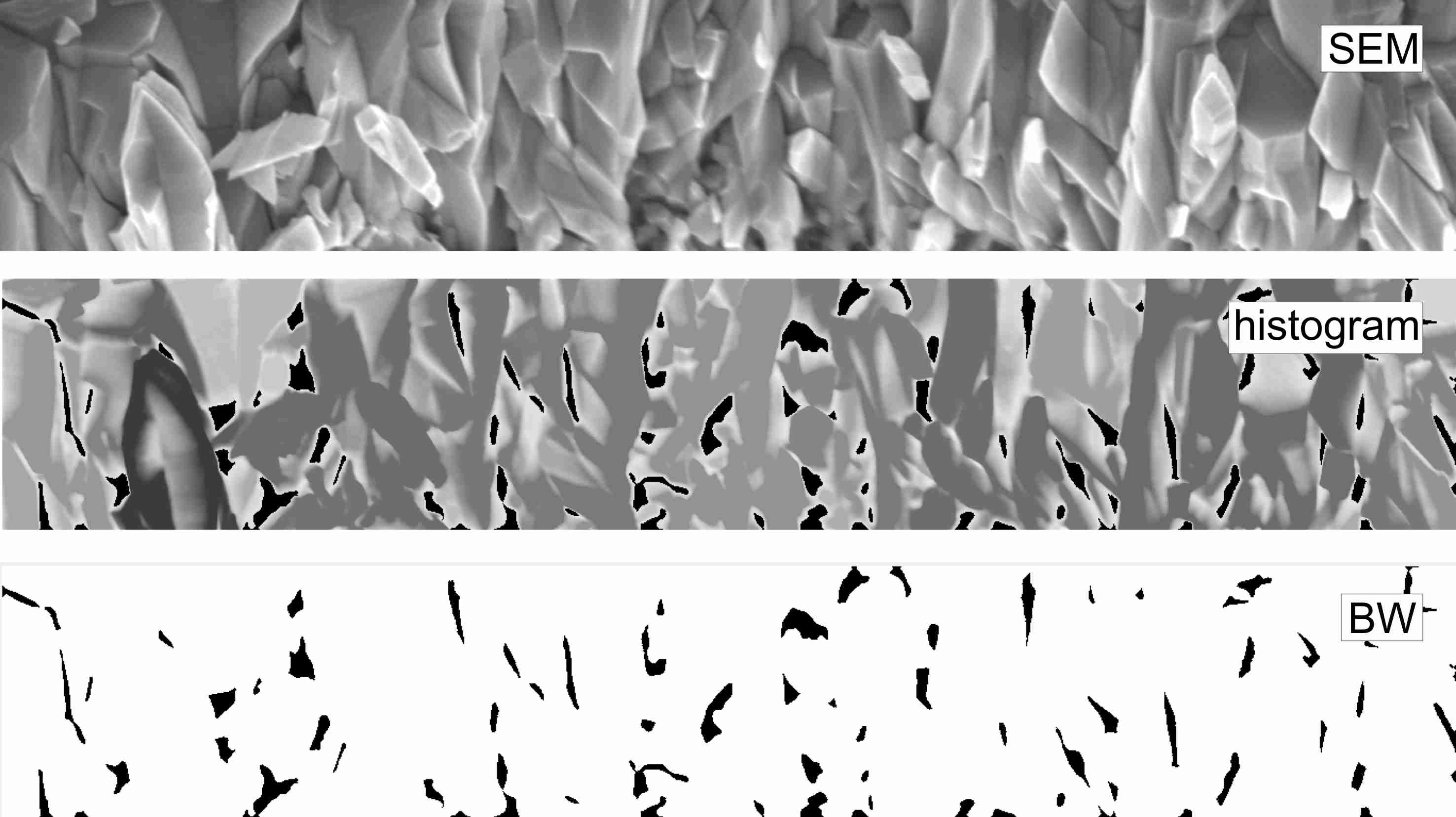


**Supporting Information Figure 3.5** Morphological operations on SEM micrographs. Original cross sectional image of sample, histogram representation of sample, and binary image of sample (sample $S_{4}$).

**Supporting Information 4***. Extended topological measures of networks and optimal choice of Waxman variables.*

Here, extensive network analysis of graphs was performed as a function of the variables of the Waxman model $\alpha$ and $\beta$ (**Supporting Information Figure 4.1**).


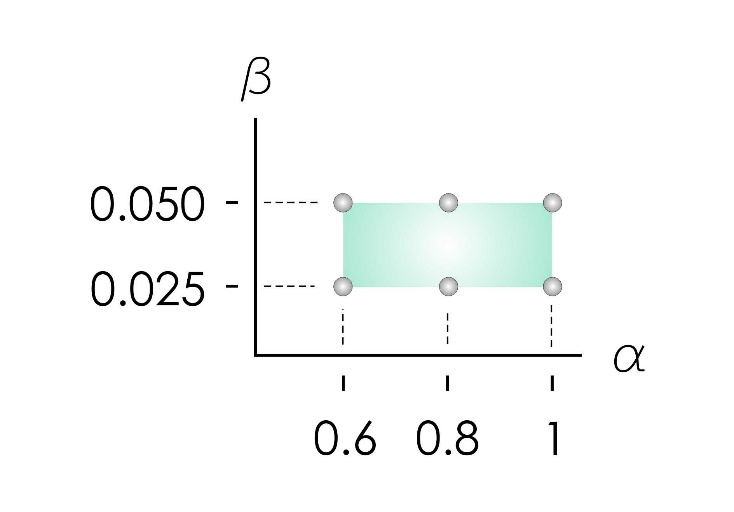


**Supporting Information Figure 4.1** we computed topological measures of networks as a function of the Waxman variables $\alpha$ and $\beta$.

$10$ topological parameters were measured using a publicly released code^7^ available at:

<https://github.com/biomedical-cybernetics/topological_measures_wide_analysis>

Network measures can be stochastic or deterministic. The stochastic measures included in the present study are three: *small-world-ness* (two different types) and *structural consistency*.

Deterministic measures are based on the direct quantification of a considered network topology feature. The deterministic measures included in the present study are seven: *characteristic path length*, *average clustering coefficient*, *efficiency* (two different types), *closeness centrality*, *node betweenness centrality*, *edge betweenness centrality*.

Topological measures of cultured neuronal networks are reported in the **Supporting Information Figures 4.2-5** for all sample numbers $S_{1}$ to $S_{5}$ and for $\alpha$ and $\beta$ varying in the intervals $\alpha\in[0.6, 1]$ and $\beta\in[0.025, 0.050]$. Network metrics show consistency and coherence for the considered variables of the model. Differences between substrates is maintained across different values of $\alpha$ and $\beta$. Therefore, results of the paper have the character of generality and the conclusions of the work, presented for the particular case $\alpha=1$, $\beta=0.025$ chosen as a reference, are reliable for all wiring conditions of neuronal cells in the considered range of $\alpha$ and $\beta$.


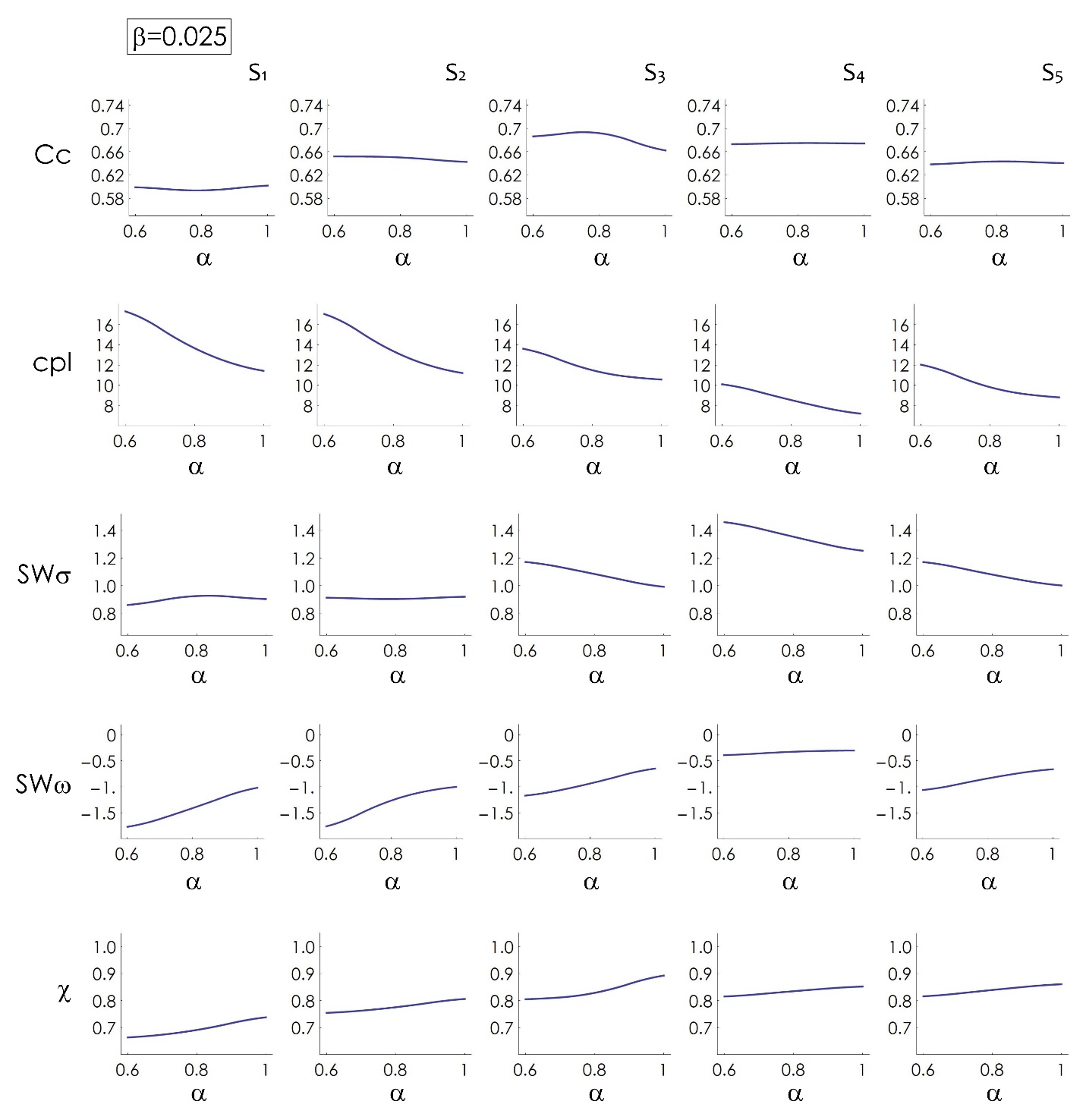


**Supporting Information Figure 4.2** topological measures of networks (clustering coefficient, characteristic path length, small worldness σ, small worldness ω, structural consistency) as a function of the Waxman variables $\alpha\in(0.6,1)$ for a fixed $\beta=0.025$.


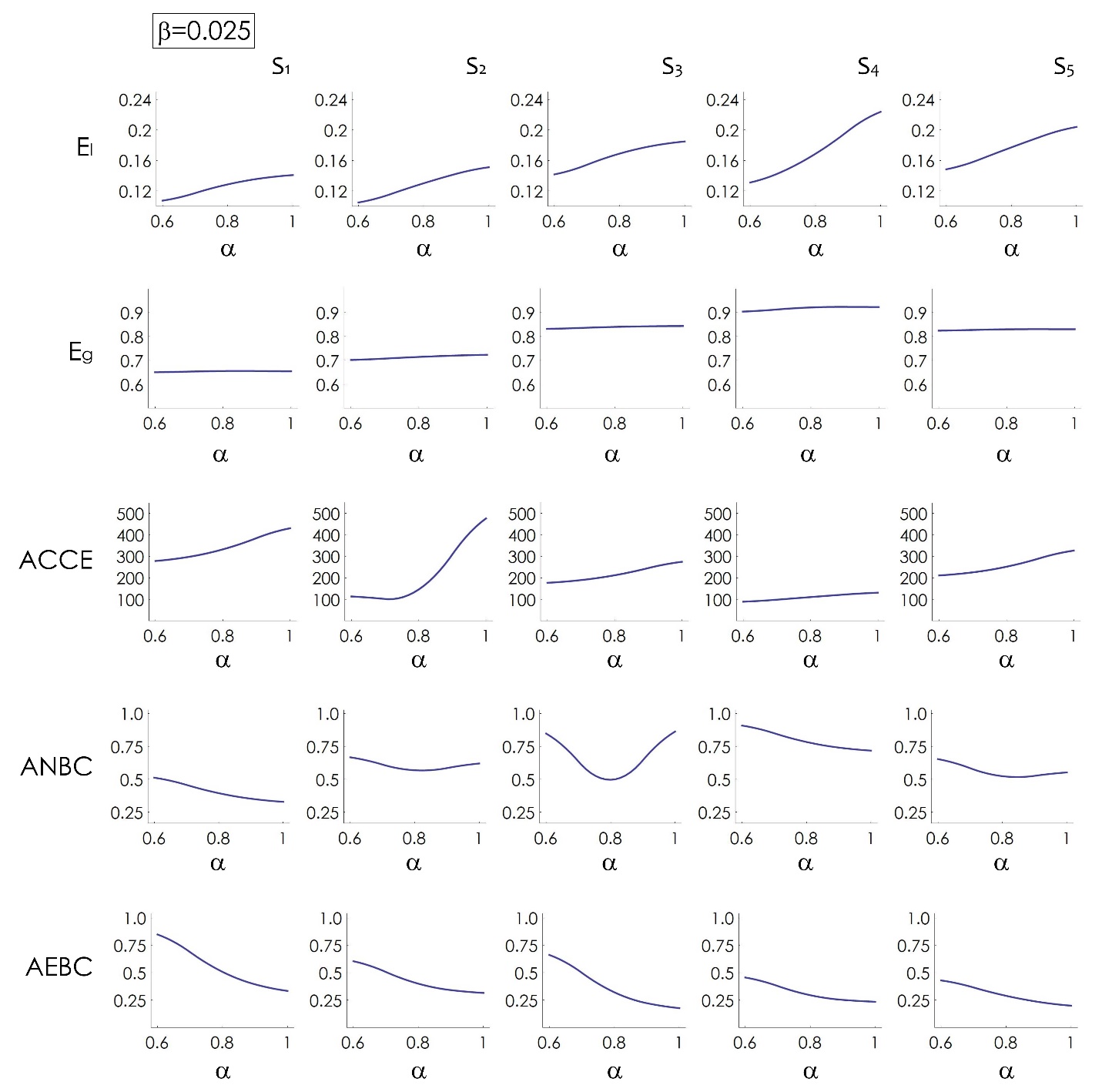


**Supporting Information Figure 4.3** topological measures of networks (local efficiency, global efficiency, average closeness centrality, average node betweeness centrality, average edge betweeness centrality) as a function of the Waxman variables $\alpha\in(0.6,1)$ for a fixed $\beta=0.025$.


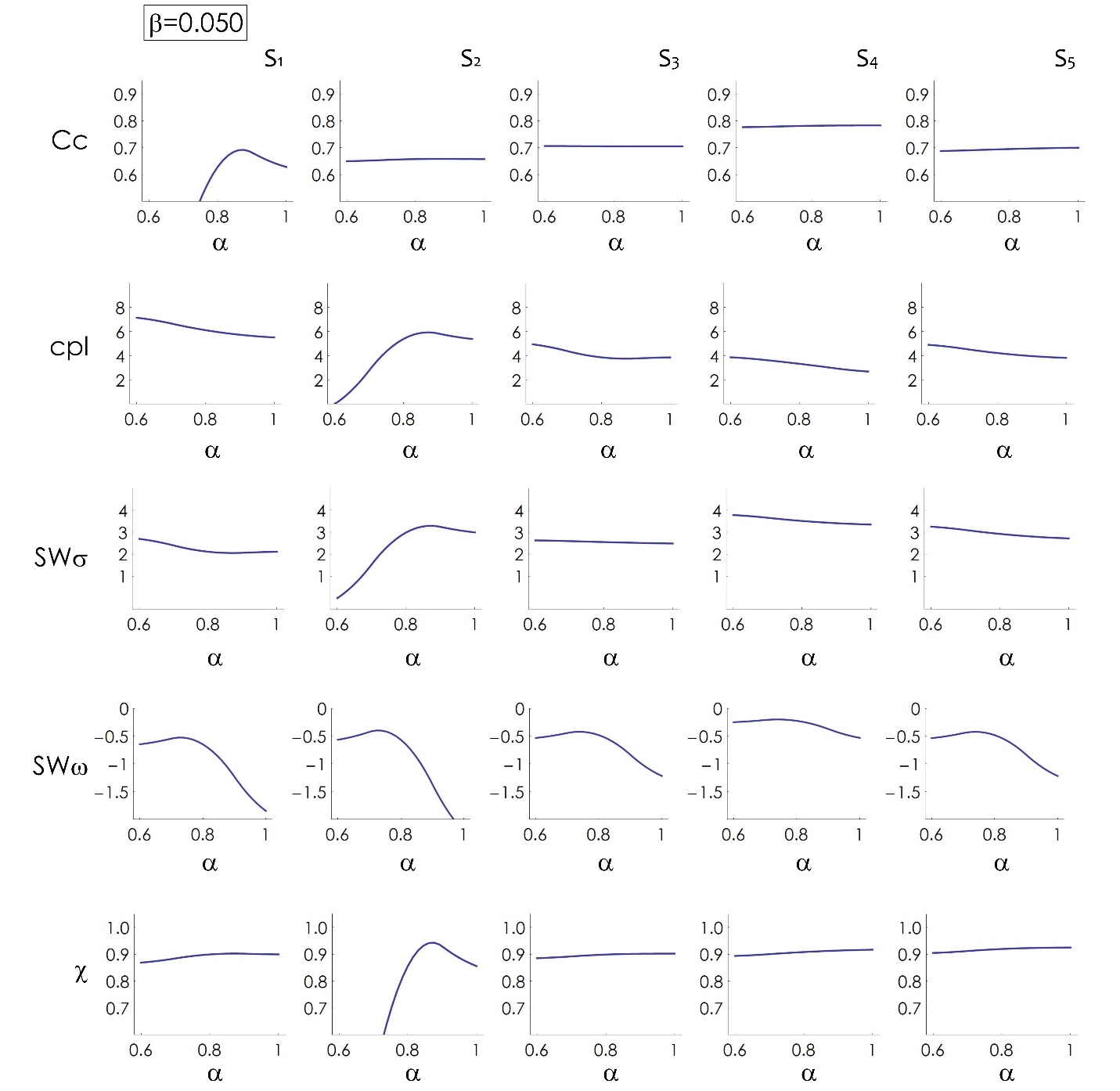


**Supporting Information Figure 4.4** topological measures of networks (clustering coefficient, characteristic path length, small worldness σ, small worldness ω, structural consistency) as a function of the Waxman variables $\alpha\in(0.6,1)$ for a fixed $\beta=0.050$.


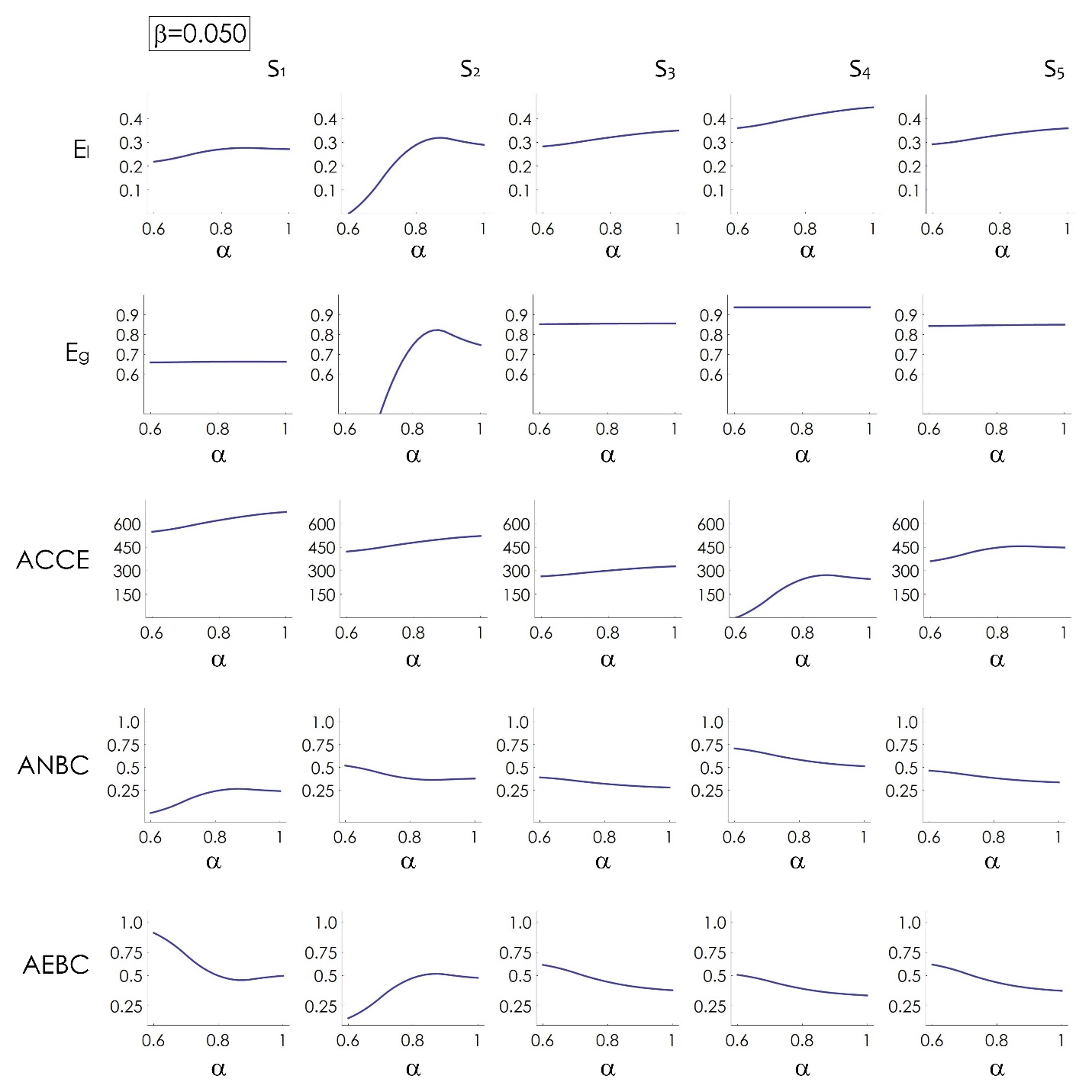


**Supporting Information Figure 4.5** topological measures of networks (local efficiency, global efficiency, average closeness centrality, average node betweeness centrality, average edge betweeness centrality) as a function of the Waxman variables $\alpha\in(0.6,1)$ for a fixed $\beta=0.050$.

**1** *Small-worldness omega* (SWσ). To calculate this measure, the average clustering coefficient C and characteristic path length L of the network are measured and compared with respect to C and L of an equivalent random network (with similar node degree distribution) and the ratio is determined as follows. γ = C/C_rand_ and λ = L/L_rand_. These ratios give the small world coefficient σ = γ/λ. A condition for a network to exhibit small world features is that the characteristic path length should be close to that of an equivalent random network, L ≈ L_rand_. And the average clustering coefficient should be close to that of an equivalent lattice network, which also implies that C should be much higher than that of equivalent random network, C ≫ C_rand_. These boundary conditions, if met, restrict the value of σ > 1 for small world networks.

**2** *Small-worldness sigma* (SWω). L of a small world network is near to L_rand_ of an equivalent random network, and C of a small world network is near to C_latt_ of an equivalent lattice network. In ‘ω’ the ratio of the average clustering coefficient is calculated with respect to an equivalent lattice network and the ratio of the characteristic path length is calculated with respect to an equivalent random network:

| $\omega=\frac{L_{rand}}{L}-\frac{C}{C_{latt}}$ | (SI 4.1) |
| --- | --- |

Hence this measure neglects fluctuations of C_rand_. Since for small world networks boundary condition is that L ≈ L_rand_ and C ≈ C_latt_, the values of ω come out to be near 0 for small world networks. The equation suggests that ω ∈ [−1, 1], with positive values suggesting the network having more randomness, L ≈ L_rand_∧ C ≪ C_latt_, and negative values suggesting network to be more latticed, L ≫ L_rand_ ∧ C ≈ C_latt_.

**3** *The structural consistency* (SC) is a global measure and a quantitative index for measuring the link predictability of a complex network. The link predictability quantifies the inherent facility to predict the missing or non-observed links of a complex network regardless of the specific algorithm used for the prediction. SC relies on the random perturbation (which is origin of stochasticity) and first-order approximation of the adjacency matrix. The hypothesis on which is based this measure suggests that a group of links is predictable if removing them has only a small effect on the network structural features. In fact, the topological regularity of a network is reflected in the consistency of structural features before and after a random removal of a small set of links. This measure exists in the interval $[0, 1]$, where $0$ indicates absence of link predictability and $1$ indicates full link predictability.

**4** The average shortest path distance a.k.a. *characteristic path length* (CPL) is a global measure and describes the average of all shortest path lengths between all the pairs of vertices. A small value of characteristic path length means that the information flow between the nodes across the network is facilitated, and that the nodes are able to send messages with each other easily. In other words the nodes across connectomes are functionally convergent.

**5** *The average efficiency* (AE) is a global measure and quantifies how efficiently the information is exchanged within the network. It is inversely proportional to the CPL. If the CPL is low then efficiency is high.

**6** *The average clustering coefficient* (ACC) is a local measure and offers an average evaluation of the cross-interaction density between the first neighbours of each node in the network. Large values of this measure indicate that the nodes in the network tend to have highly connected neighbours.

**7** *The average closeness centrality* (ACCE) is a global measure, represents an indicator of node centrality, and calculates the average closeness of the nodes from all the others in the network. The closer a node is to the others, the faster it can spread information to the others sequentially. It is calculated by averaging all the reciprocals of the mean shortest distance of a particular node to all other nodes.

| $ACCE=\frac{1}{n}\sum_{i} \frac{n-1}{\sum_{j\neq i} {sp}_{ij}}$ | (SI 4.4) |
| --- | --- |

If the average closeness centrality of the network is low then the activity of each node would be functionally more relevant to the other nodes.

**8** *The average node betweeness centrality* (ANBC) is a global measure and also a node centrality indicator. The single node betweeness centrality measures how crucial is a particular node in maintaining an optimum information flow path between any other node pair. In other words this average measure calculates the average stress of information burden on the network nodes.

**9** *The average edge betweeness centrality* (AEBC) is a global measure and an edge centrality indicator. The single edge betweenness centrality measures the betweenness or information stress on an edge. The average measure calculates the average stress of information burden on the network edges.

**Supporting Information 5***.* *Additional fluorescence images of neuronal networks on Zinc Oxide Nanowires.*

We report additional, large format, high-definition fluorescence images of neuronal cell networks cultured on zinc oxide nanowires surfaces, in support to those presented in the main article. Images are reported for all considered substrates: $S_{1}-S_{5}$. Some of them are accompanied by the corresponding wiring diagrams obtained by applying the Waxman algorithm to the positions of the cells. First three figures are a larger format of the images of the neuronal cell networks cultured on substrates $S_{2}$, $S_{3}$, $S_{5}$ as in **Figure 5** of the main text, and not contained in **Figure 4** of the main text.


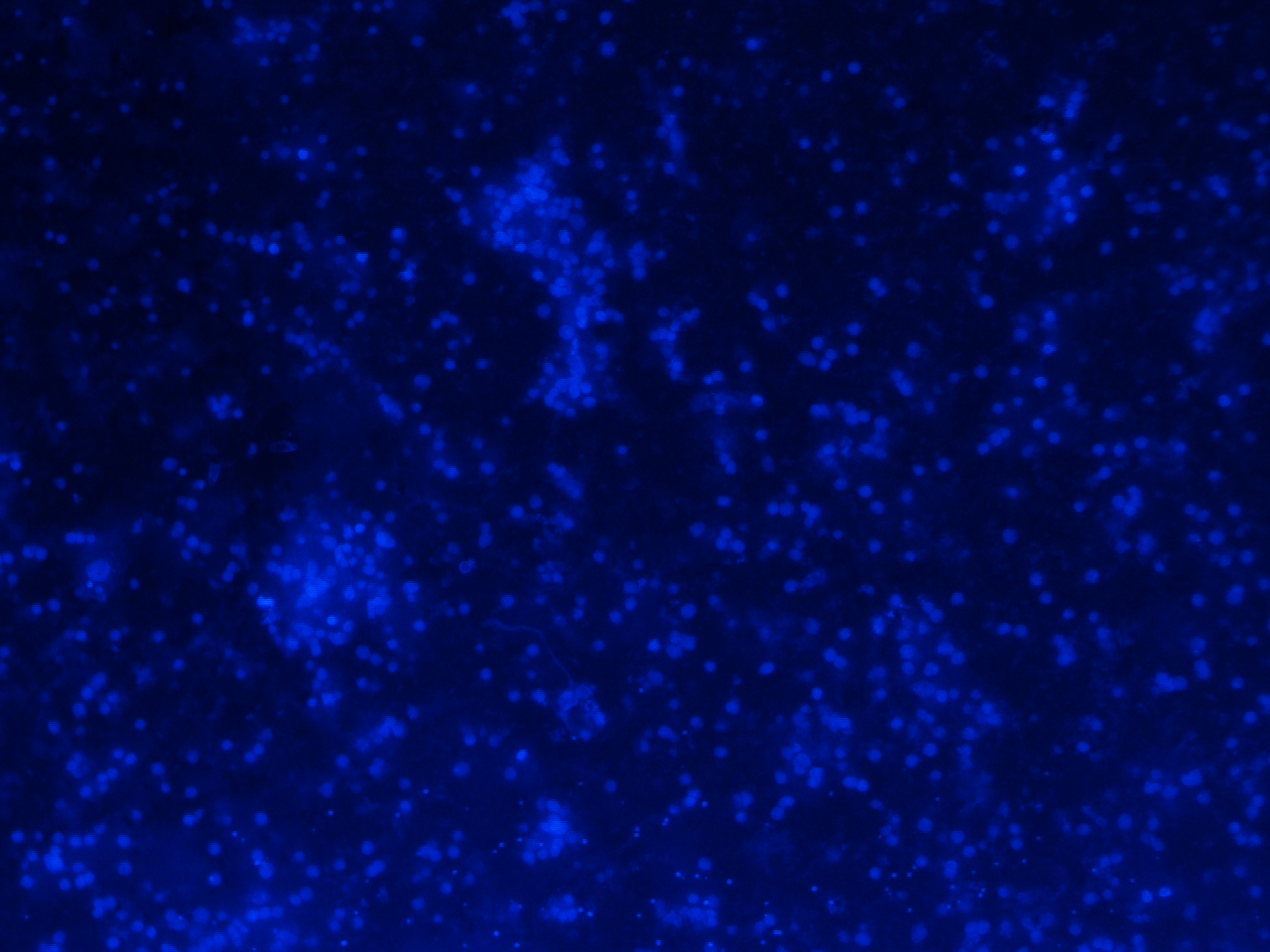


**Supporting Information Figure 5.1** Fluorescence image of neuronal cell on substrate $S_{2}$.


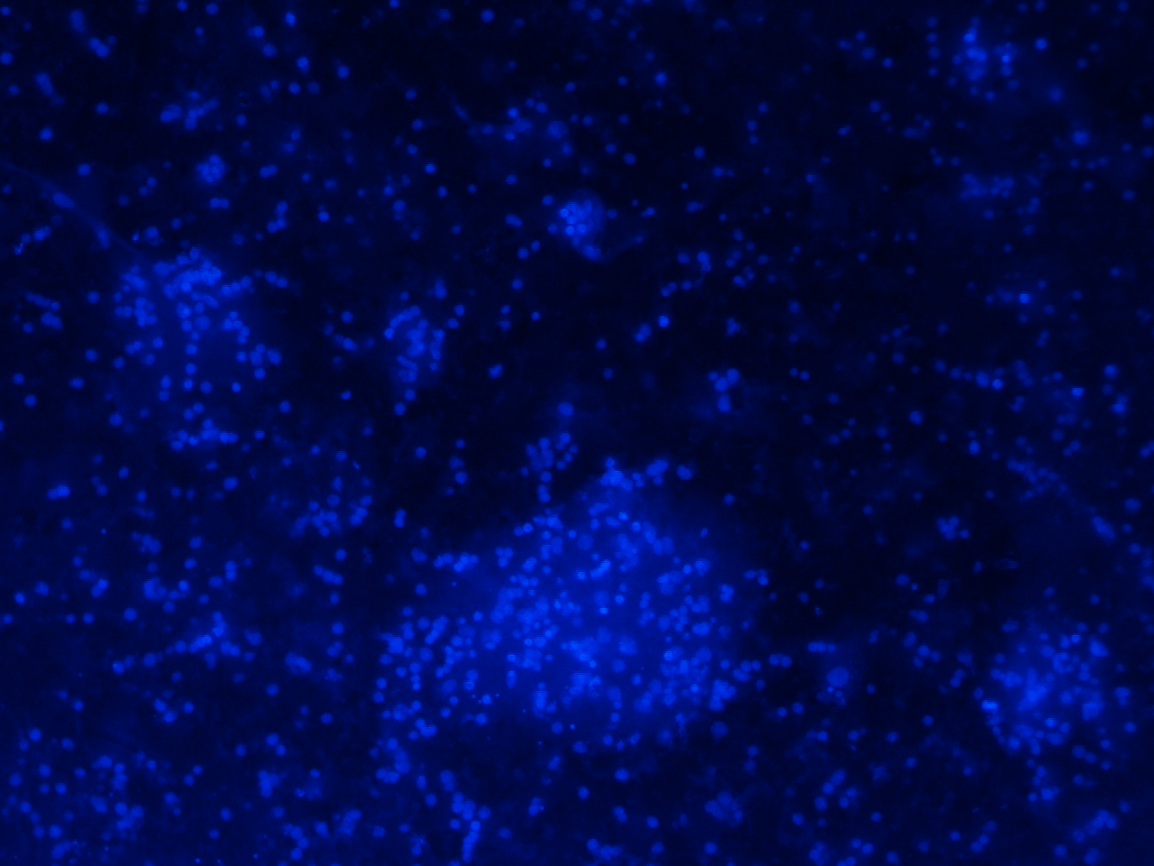


**Supporting Information Figure 5.2** Fluorescence image of neuronal cell on substrate $S_{3}$.


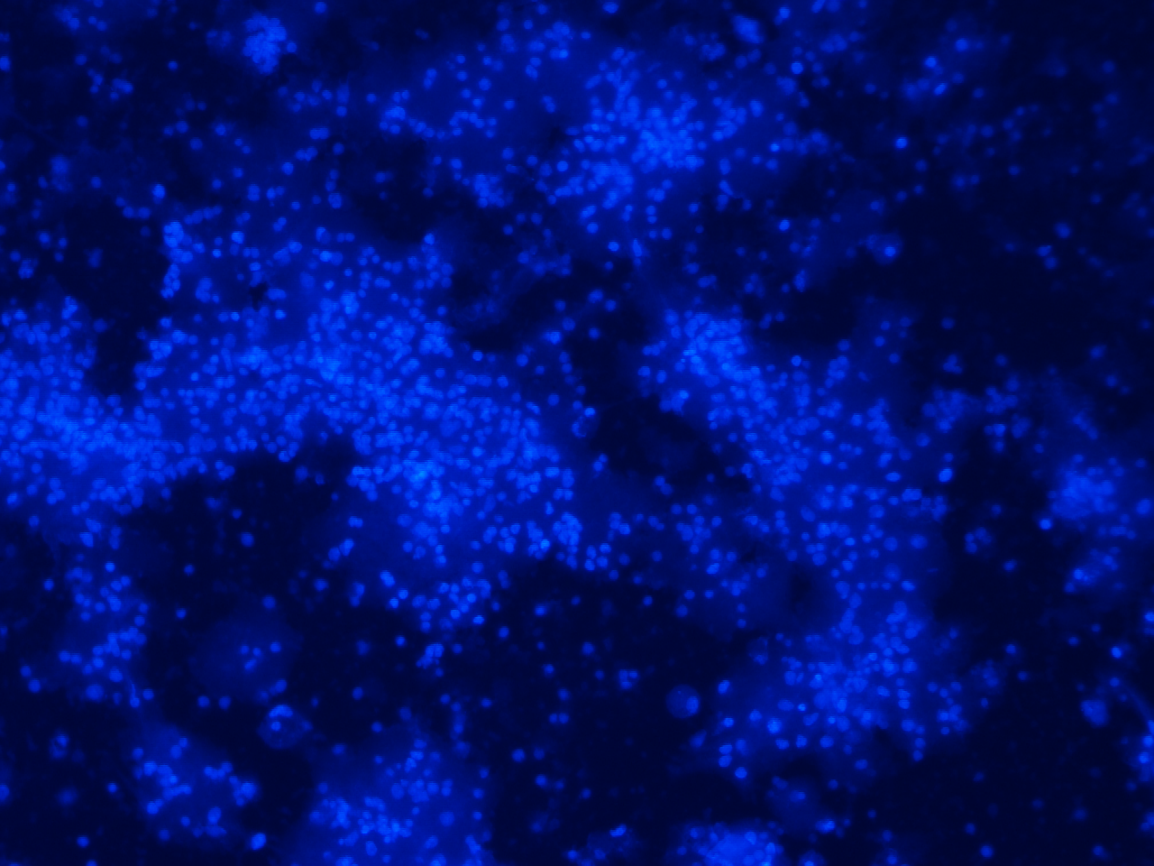


**Supporting Information Figure 5.3** Fluorescence image of neuronal cell on substrate $S_{5}$.


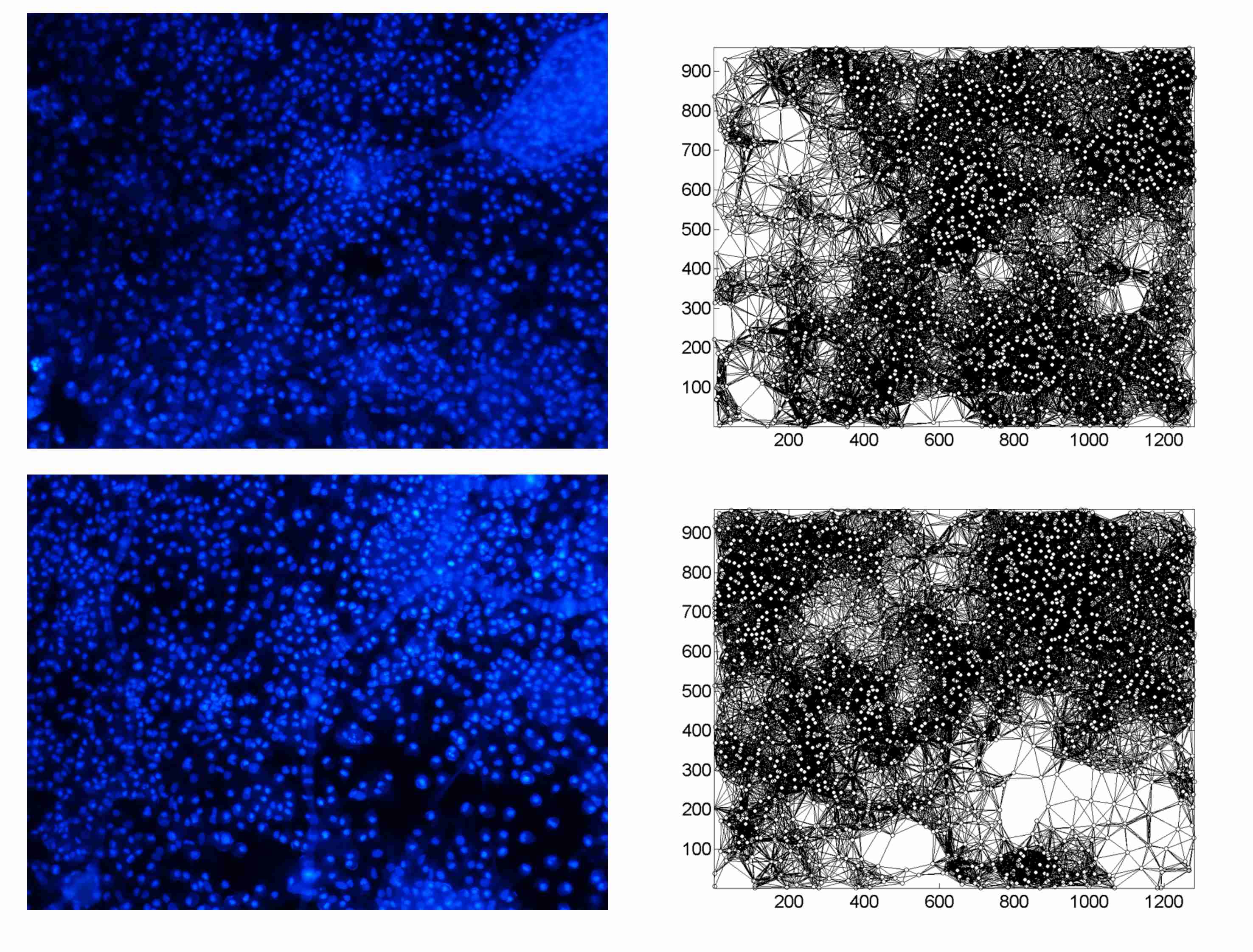


**Supporting Information Figure 5.4** Fluorescence images of neuronal cell on substrate $S_{1}$.


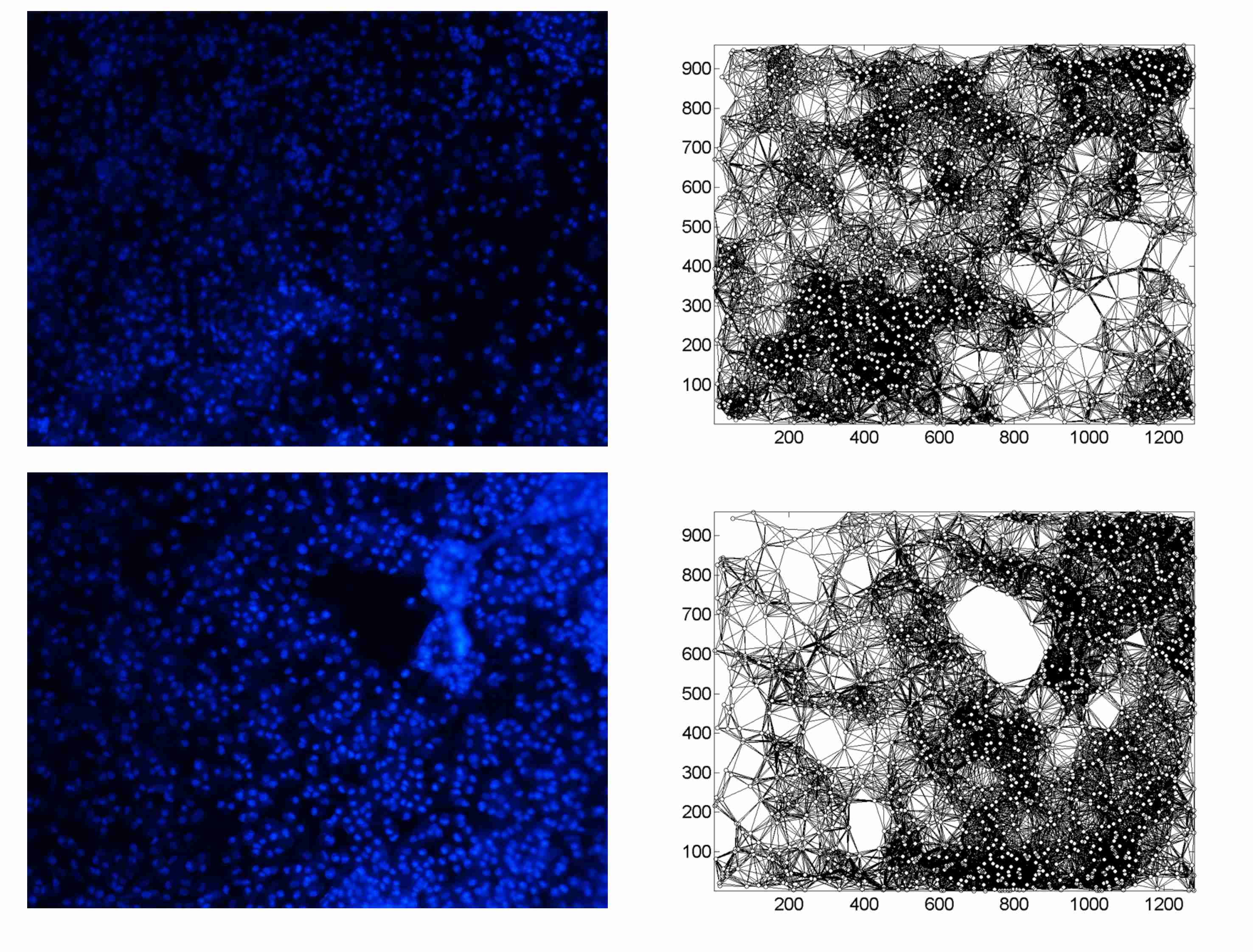


**Supporting Information Figure 5.5** Fluorescence images of neuronal cell on substrate $S_{1}$.


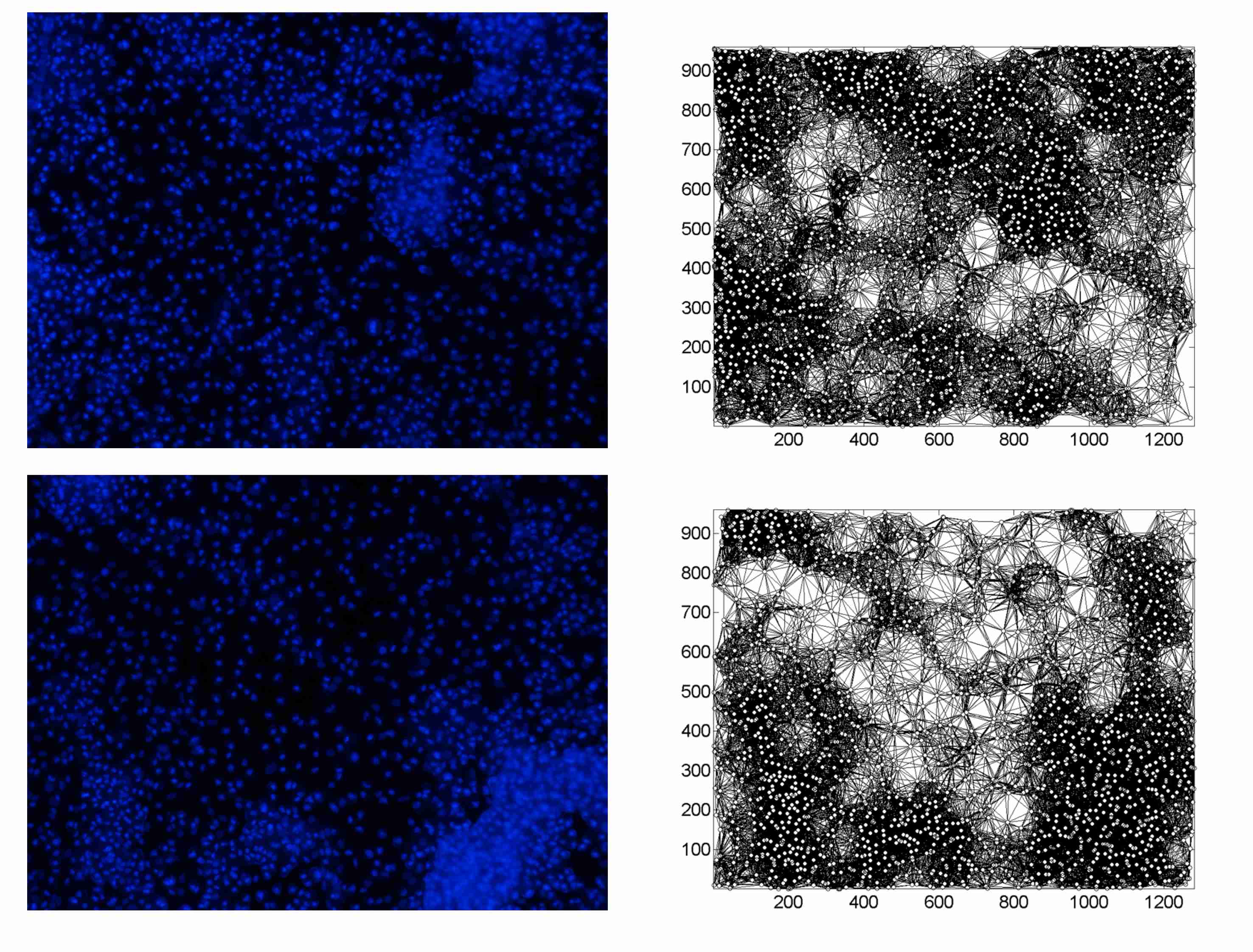


**Supporting Information Figure 5.6** Fluorescence images of neuronal cell on substrate $S_{1}$.


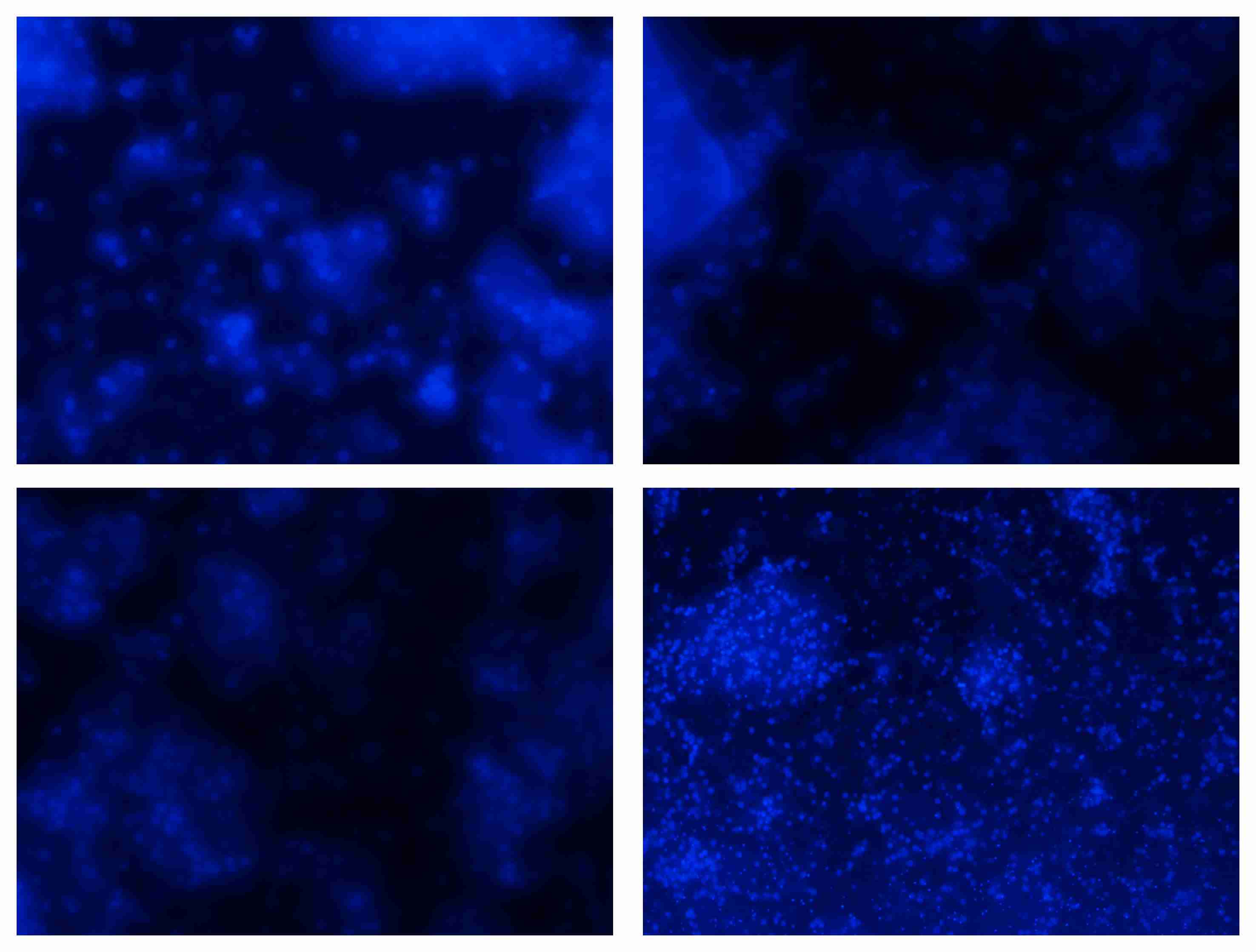


**Supporting Information Figure 5.7** Fluorescence images of neuronal cell on substrate $S_{2}$.

**
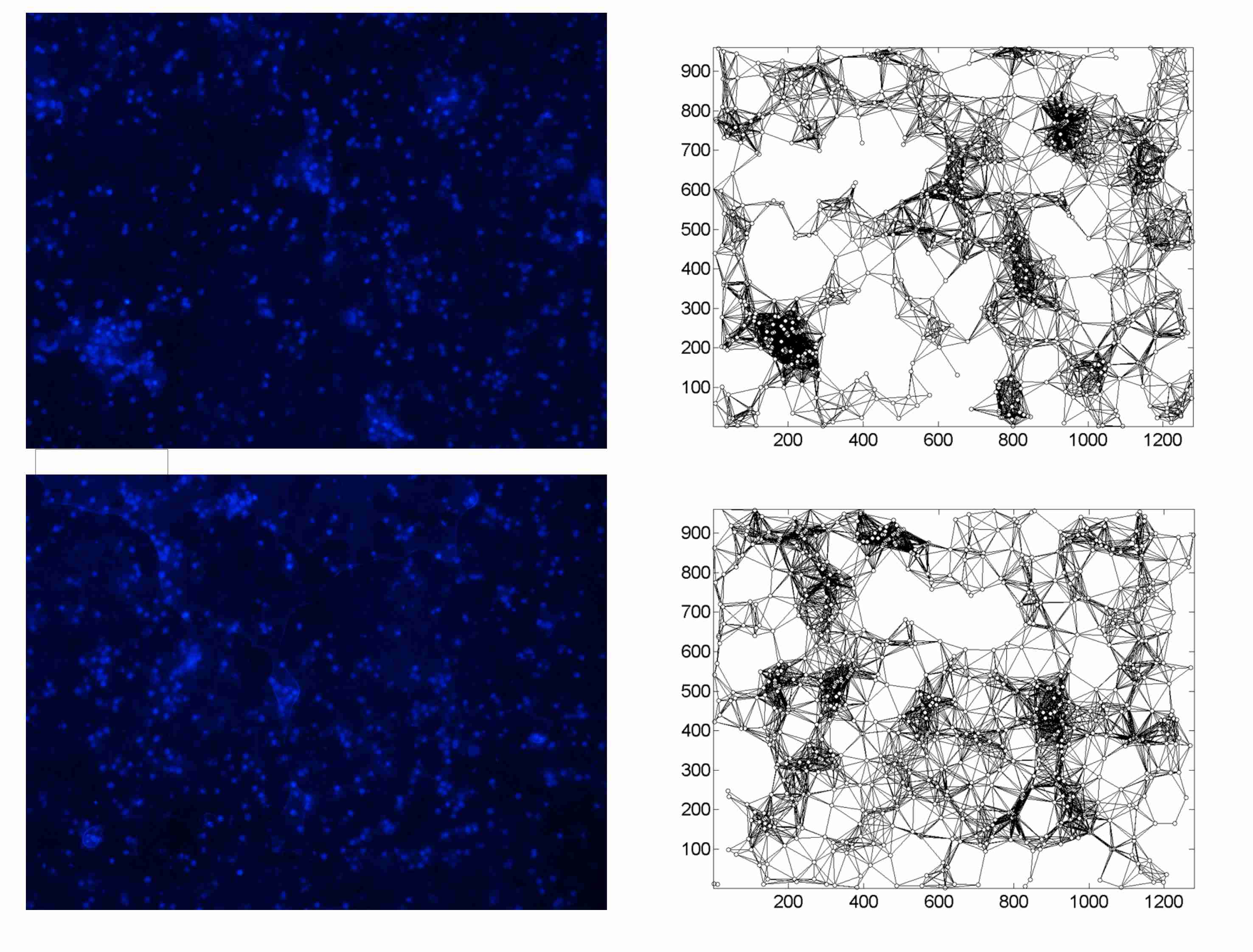
**

**Supporting Information Figure 5.8** Fluorescence images of neuronal cell on substrate $S_{2}$.

**
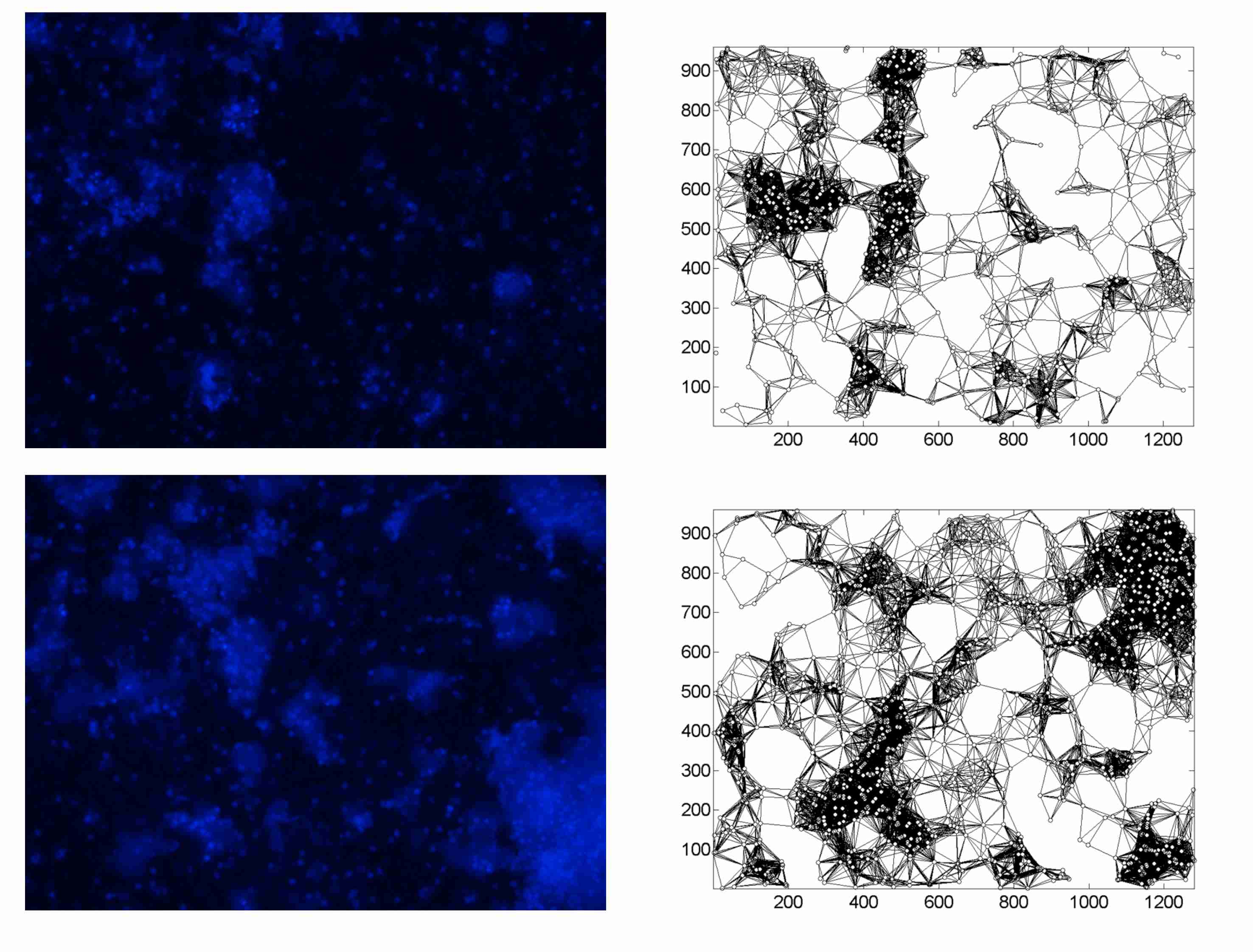
**

**Supporting Information Figure 5.9** Fluorescence images of neuronal cell on substrate $S_{2}$.

**
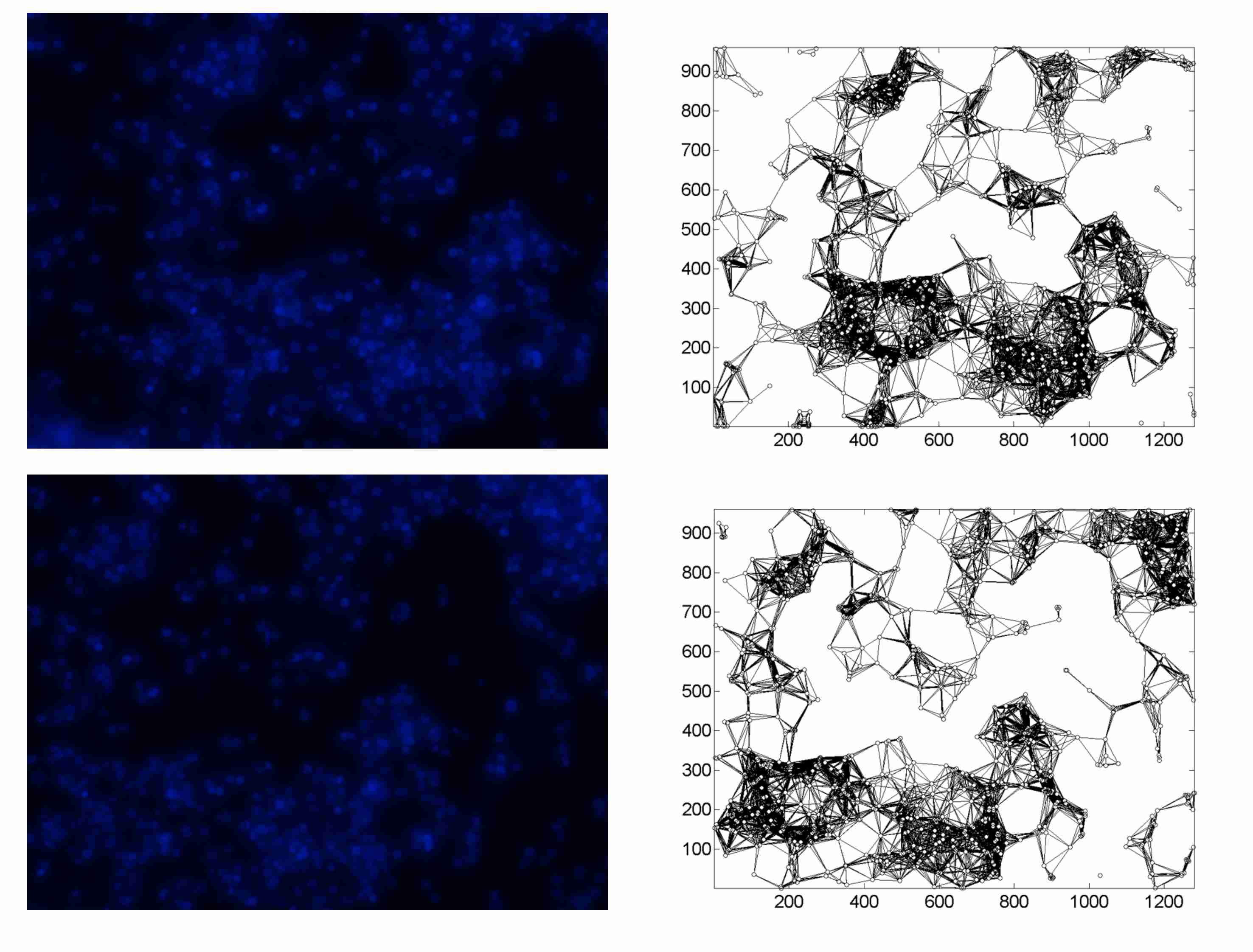
**

**Supporting Information Figure 5.10** Fluorescence images of neuronal cell on substrate $S_{3}$.

**
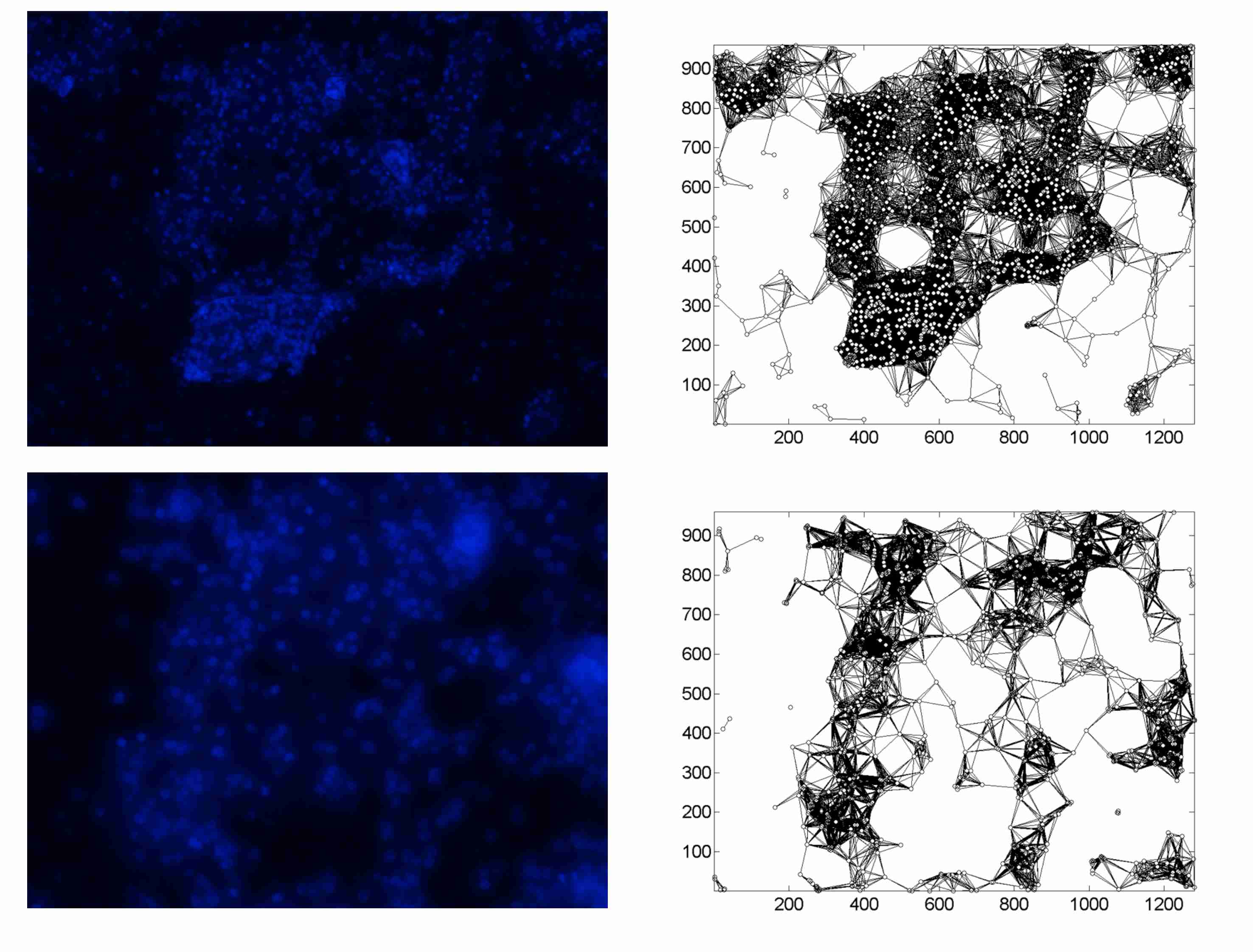
**

**Supporting Information Figure 5.11** Fluorescence images of neuronal cell on substrate $S_{3}$.

**
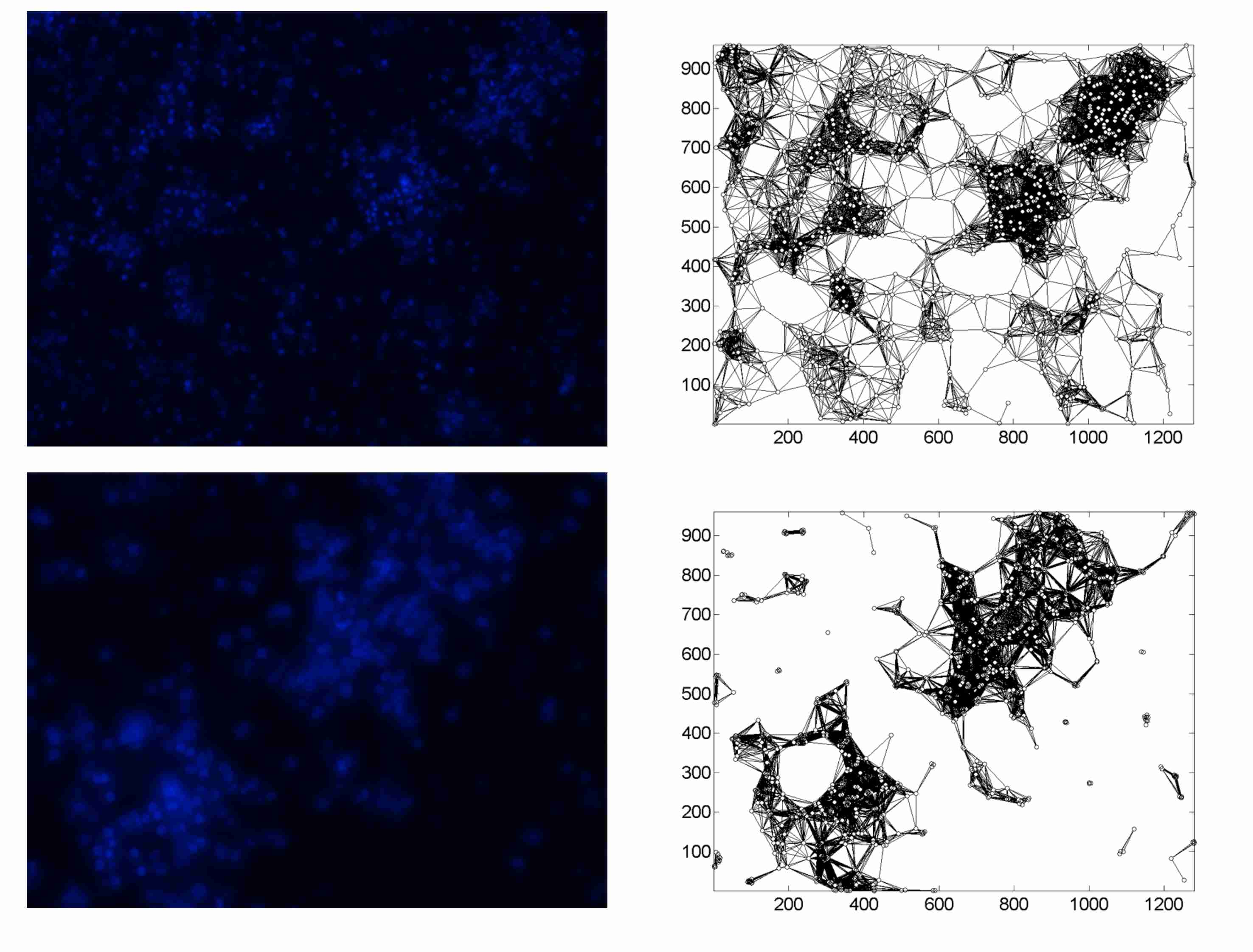
**

**Supporting Information Figure 5.12** Fluorescence images of neuronal cell on substrate $S_{3}$.

**
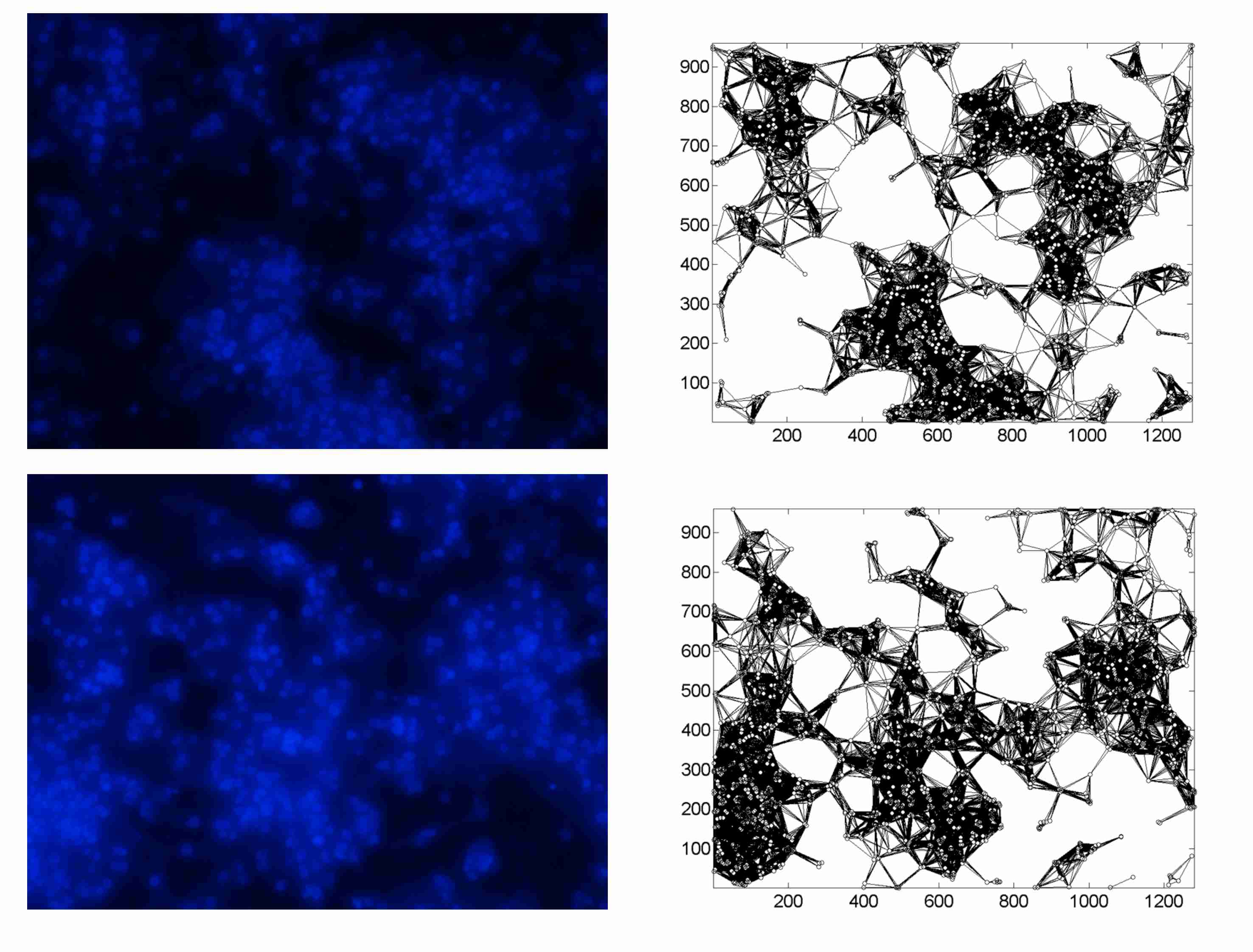
**

**Supporting Information Figure 5.13** Fluorescence images of neuronal cell on substrate $S_{4}$.

**
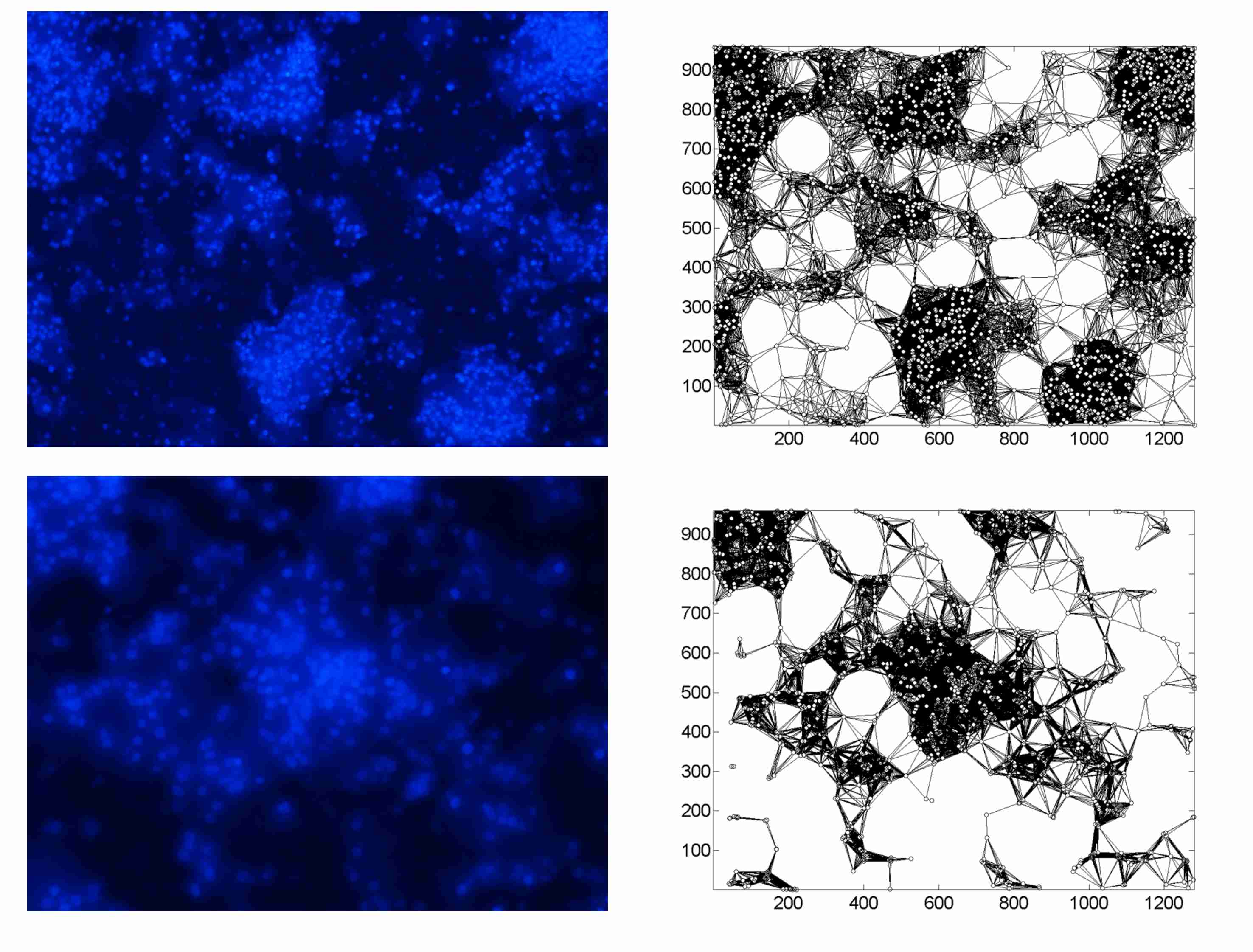
**

**Supporting Information Figure 5.14** Fluorescence images of neuronal cell on substrate $S_{4}$.

**
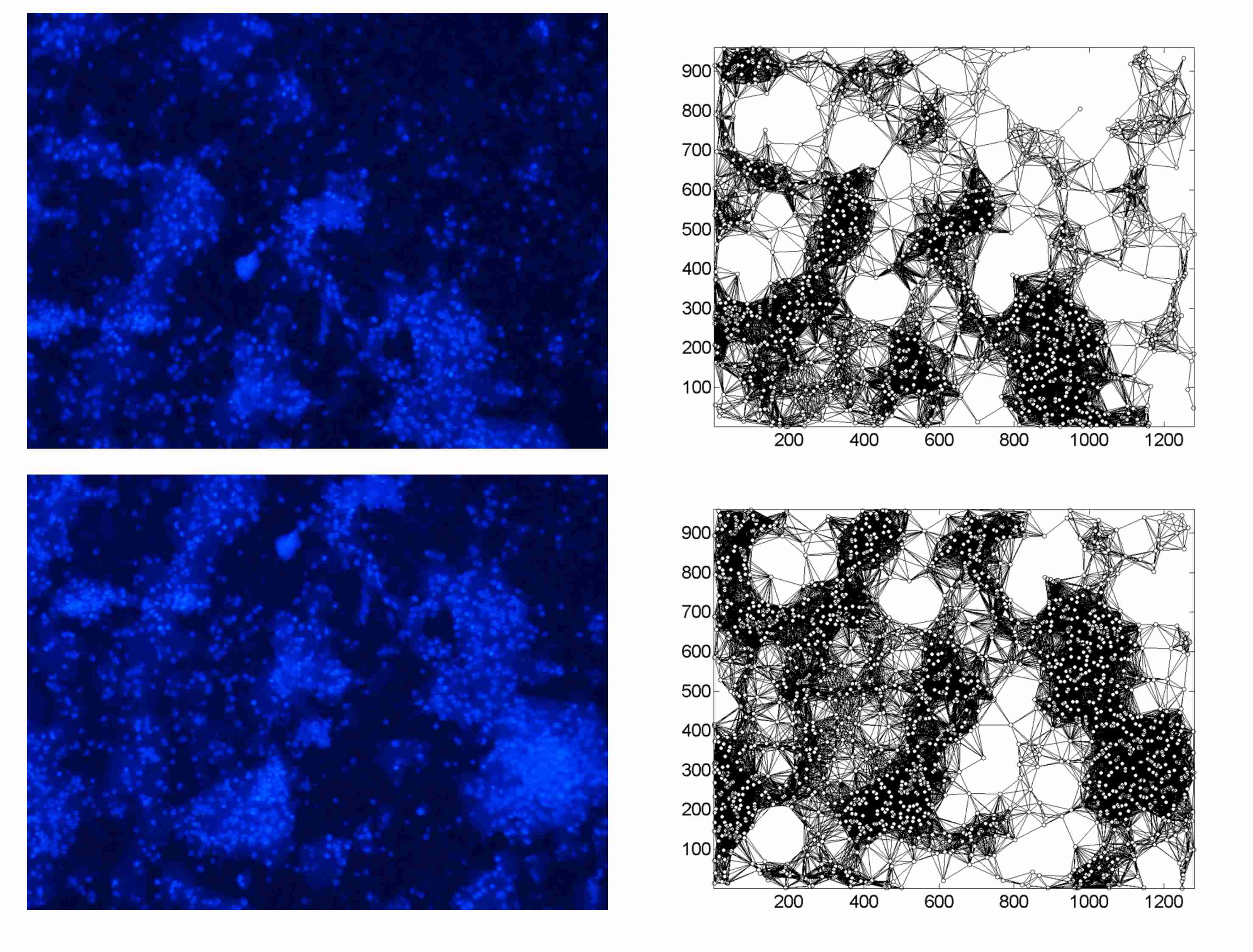
**

**Supporting Information Figure 5.15** Fluorescence images of neuronal cell on substrate $S_{4}$.

**
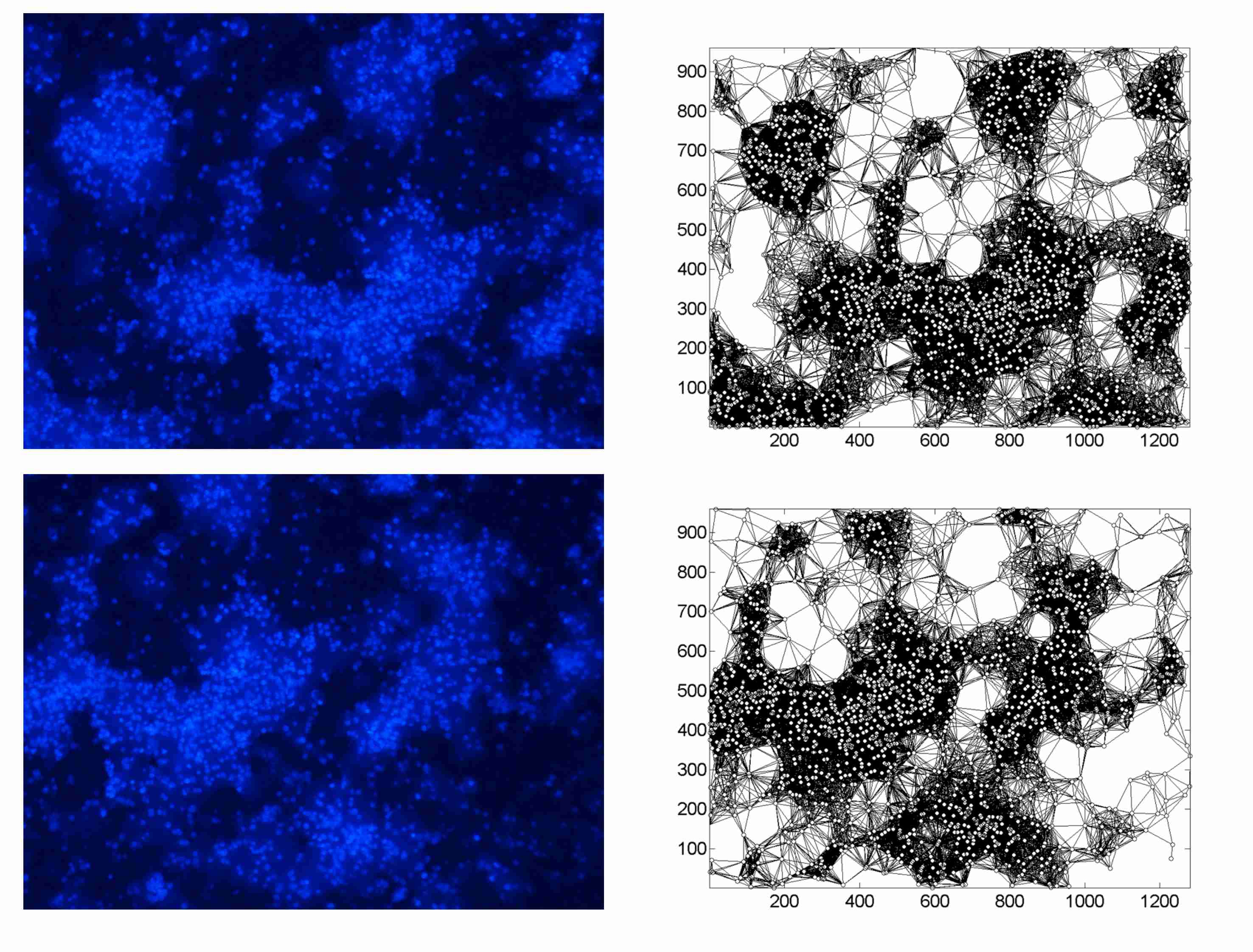
**

**Supporting Information Figure 5.16** Fluorescence images of neuronal cell on substrate $S_{5}$.

**
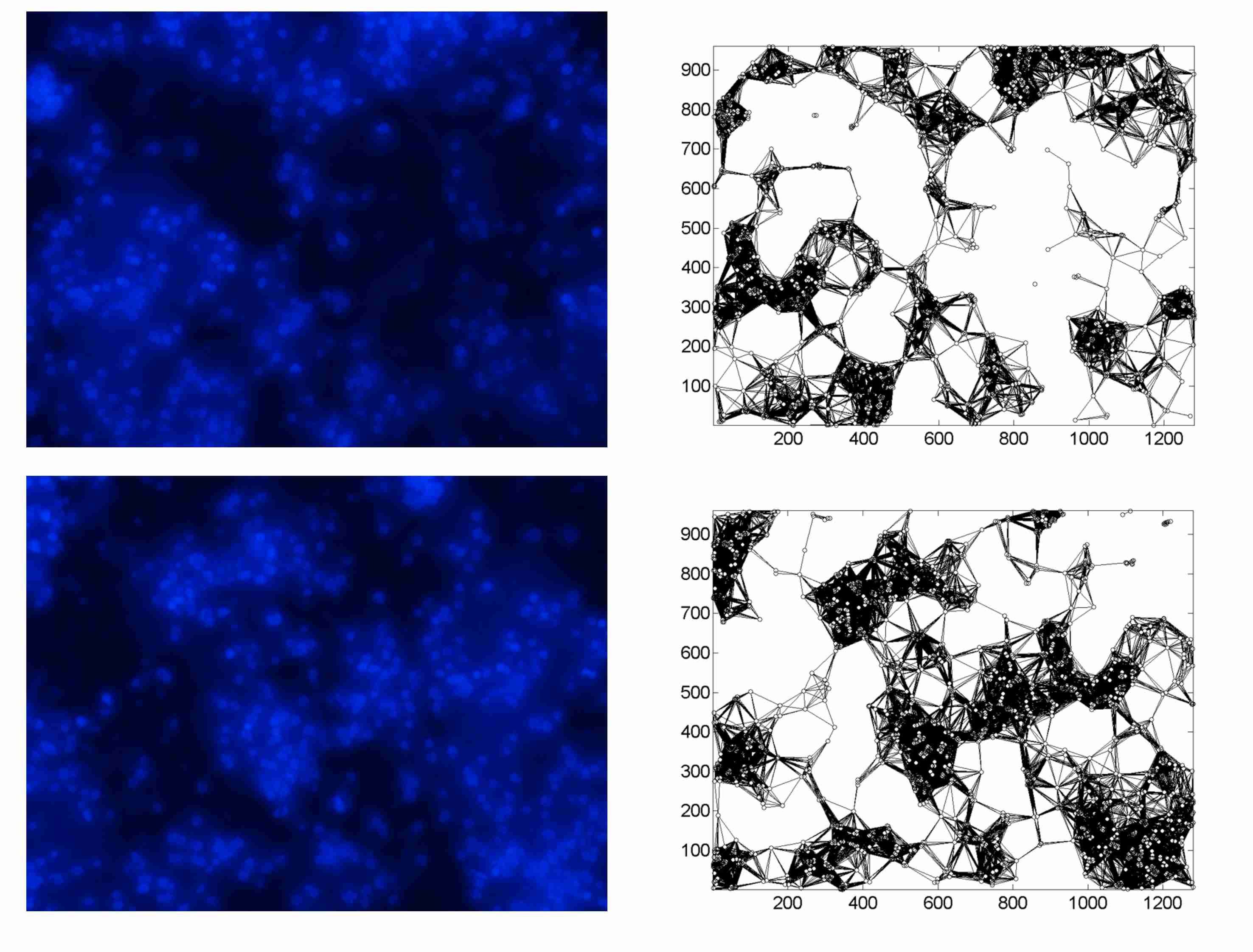
**

**Supporting Information Figure 5.17** Fluorescence images of neuronal cell on substrate $S_{5}$.

**
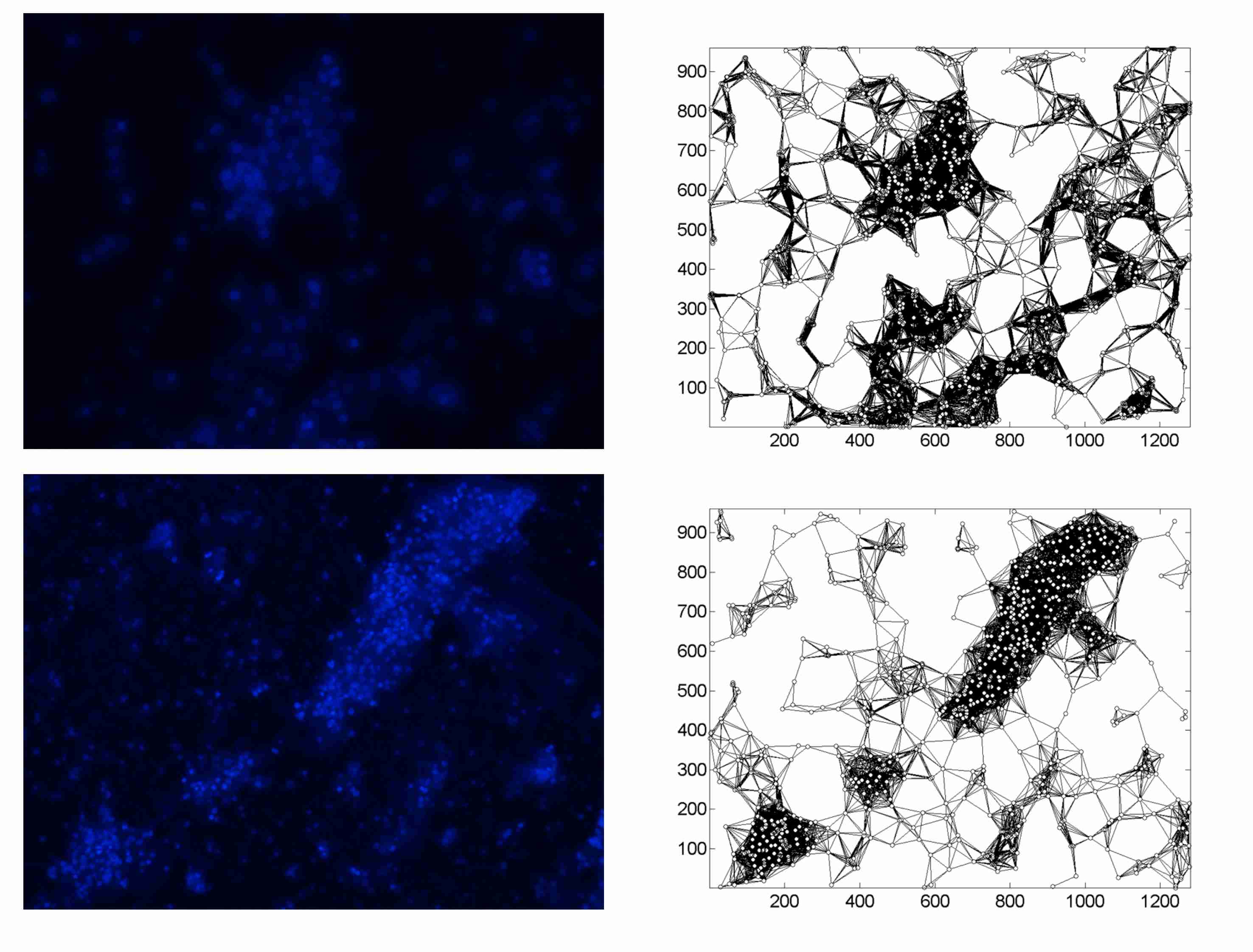
**

**Supporting Information Figure 5.18** Fluorescence images of neuronal cell on substrate $S_{5}$.

**Supporting Information 6***.* *Evaluating the level of power-lawness of the distribution of the degree of the graphs* $\kappa$.

Here, we show results of a power-law test that demonstrate that the distribution of the degree of the graphs $\kappa$ is not power-law, but has an exponential decay. We used the test for power-lawness described in^7^.

The power-lawness (PL) is a local measure that offers a confidence level to claim whether power-law node distribution governs a particular network or not^7^. To ensure power law as a plausible hypothesis, p-value is calculated. If the calculated p-value $\geq0.1$, the power law hypothesis is accepted for the network, otherwise rejected.

Calculated values of p-values are $0$ for the networks for each substrate preparation, as shown in the **Supporting Information Table 6.1**.

| *Substrate* | *p-value* |
| --- | --- |
| $\mathbf{S}_{\mathbf{1}}$ | $0$ |
| $\mathbf{S}_{\mathbf{2}}$ | $0$ |
| $\mathbf{S}_{\mathbf{3}}$ | $0$ |
| $\mathbf{S}_{\mathbf{4}}$ | $0$ |
| $\mathbf{S}_{\mathbf{5}}$ | $0$ |

**Supporting Information Table 6.1**

Moreover, log-log plots of the distributions reported in the **Supporting Information Figure 6.1** show that the decay is exponential and not power-law.


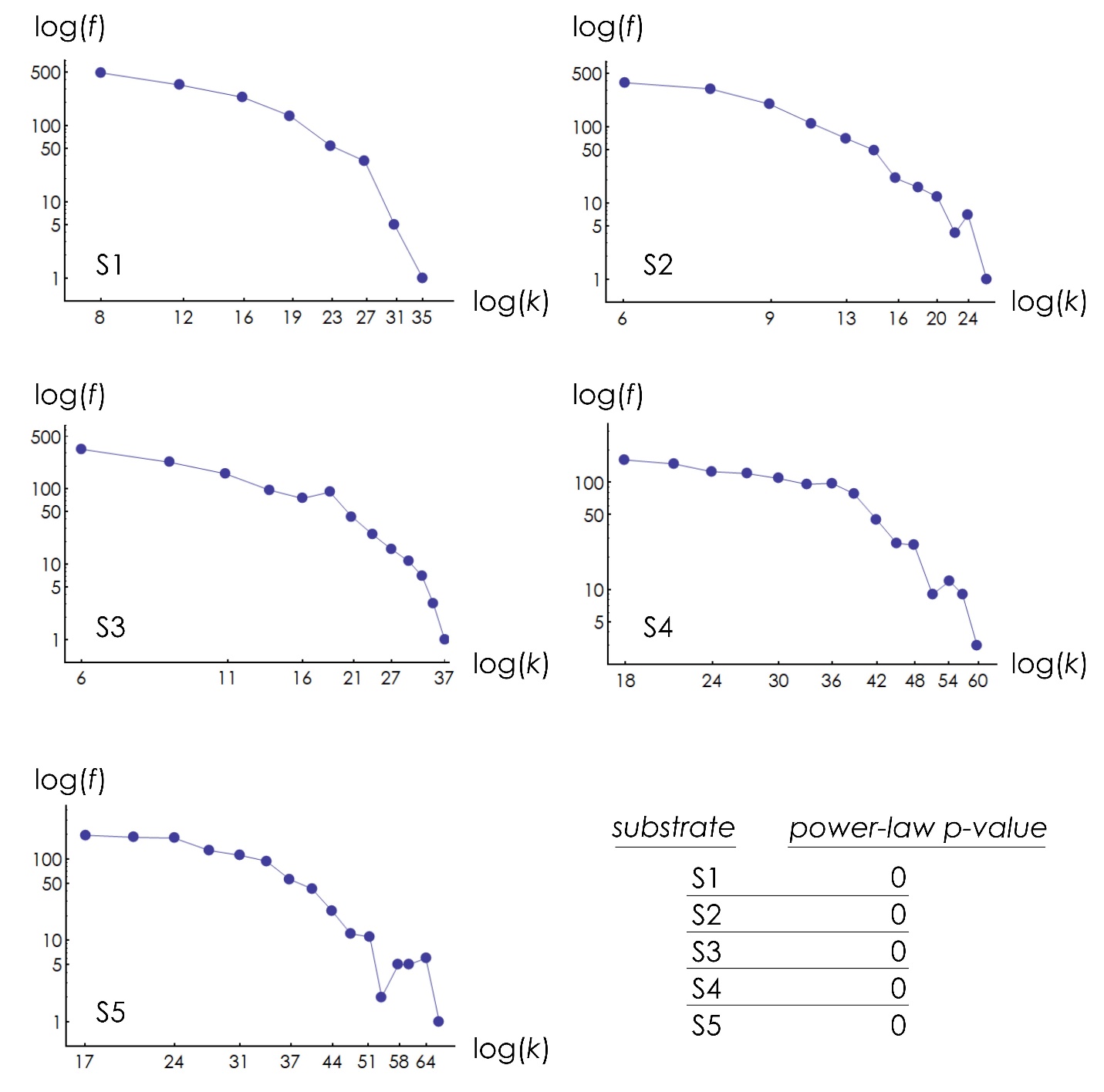


**Supporting Information Figure 6.1**

**Supporting Information 7***. Imaging neurites in cultured neural networks.*

We used Phalloidin that selectively binds to F-Actin and fluorescence microscopy (Methods) for imaging neurites in cultured neural networks. Actin is a family of globular multi-functional proteins that form microfilaments in the cytoskeleton. It is comprised in axons in neural cells and its content indicates progress of neurite growth and formation in neuronal morphogenesis and neurite branching^8,9^. Upon staining, we observe that, for high substrate numbers $S_{n}>S_{2}$, anatomical connectivity among individual neurons exhibits small world architectures, with few clusters featuring sparse connections inter-cluster and over-abundance of connections intra-cluster (**Supporting Information Figures 7.1-7.5**).

**
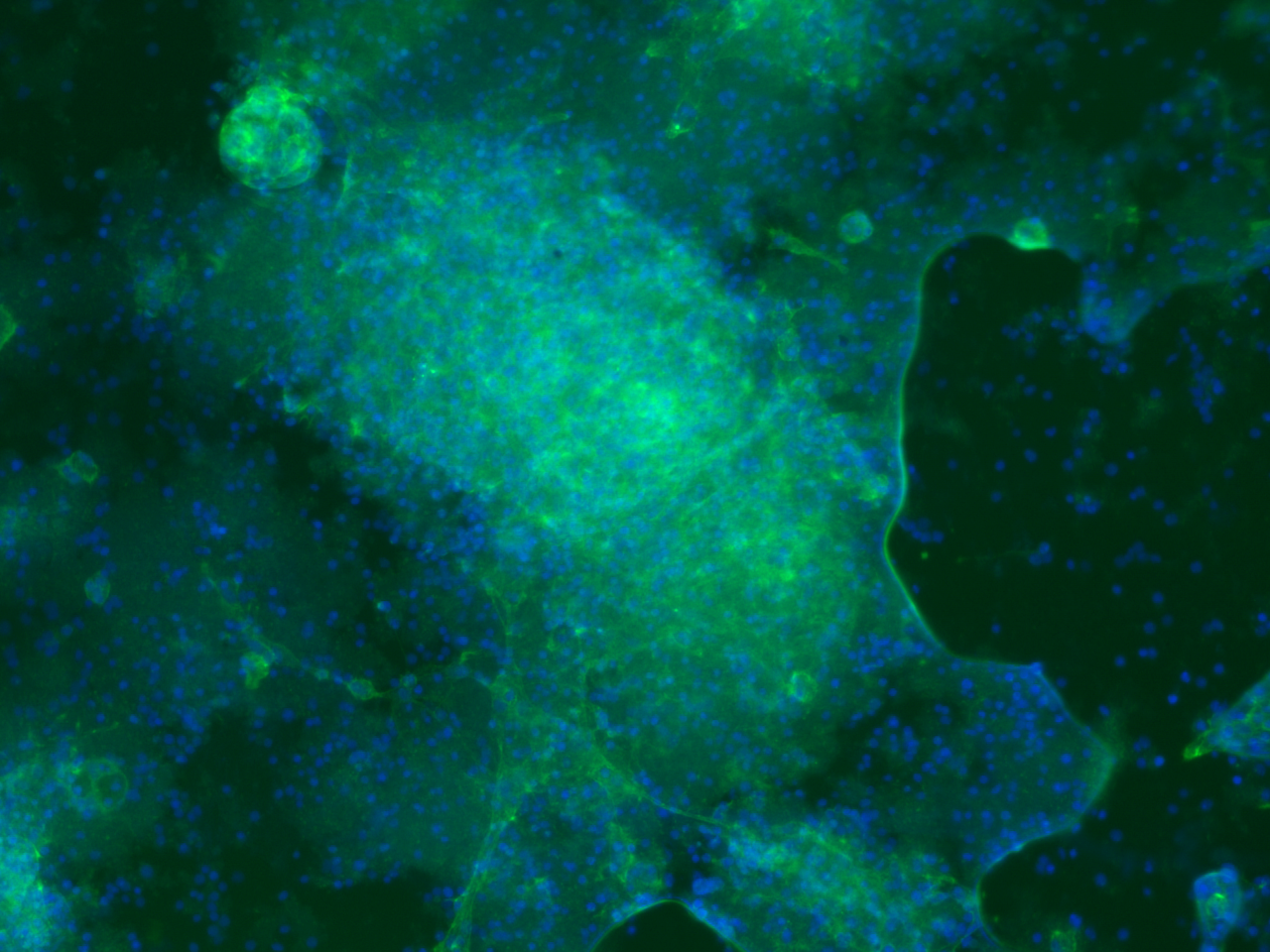
**

**Supporting Information Figure 7.1** Phalloidin + DAPI staining of cells ($S_{1}$).


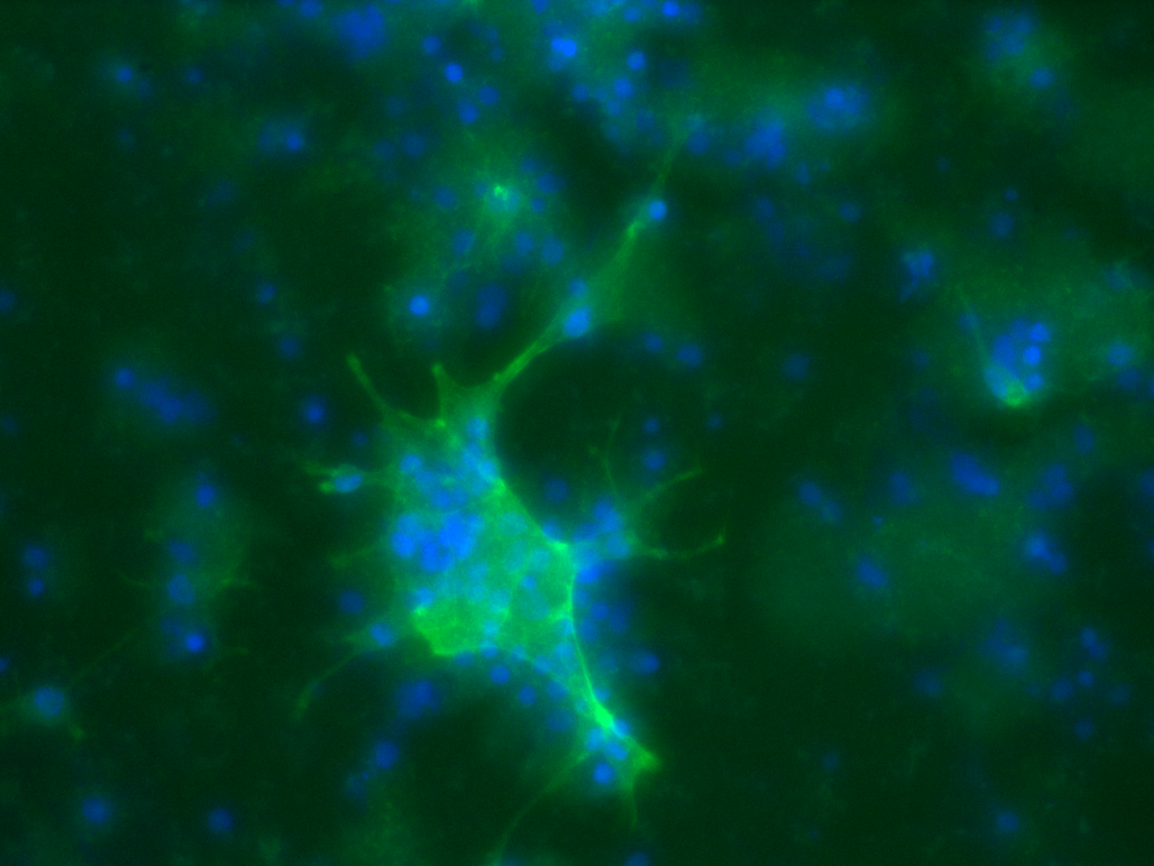


**Supporting Information Figure 7.2** Phalloidin + DAPI staining of cells ($S_{2}$).


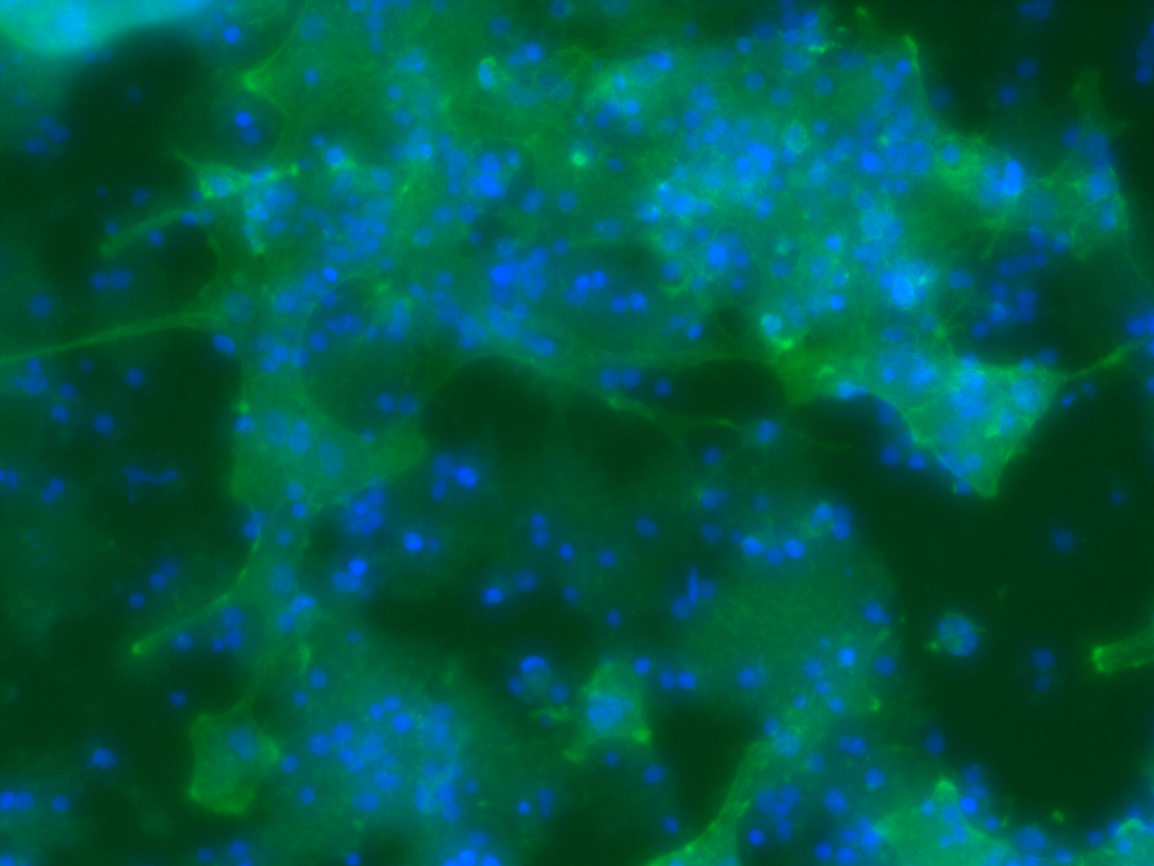


**Supporting Information Figure 7.3** Phalloidin + DAPI staining of cells ($S_{3}$).


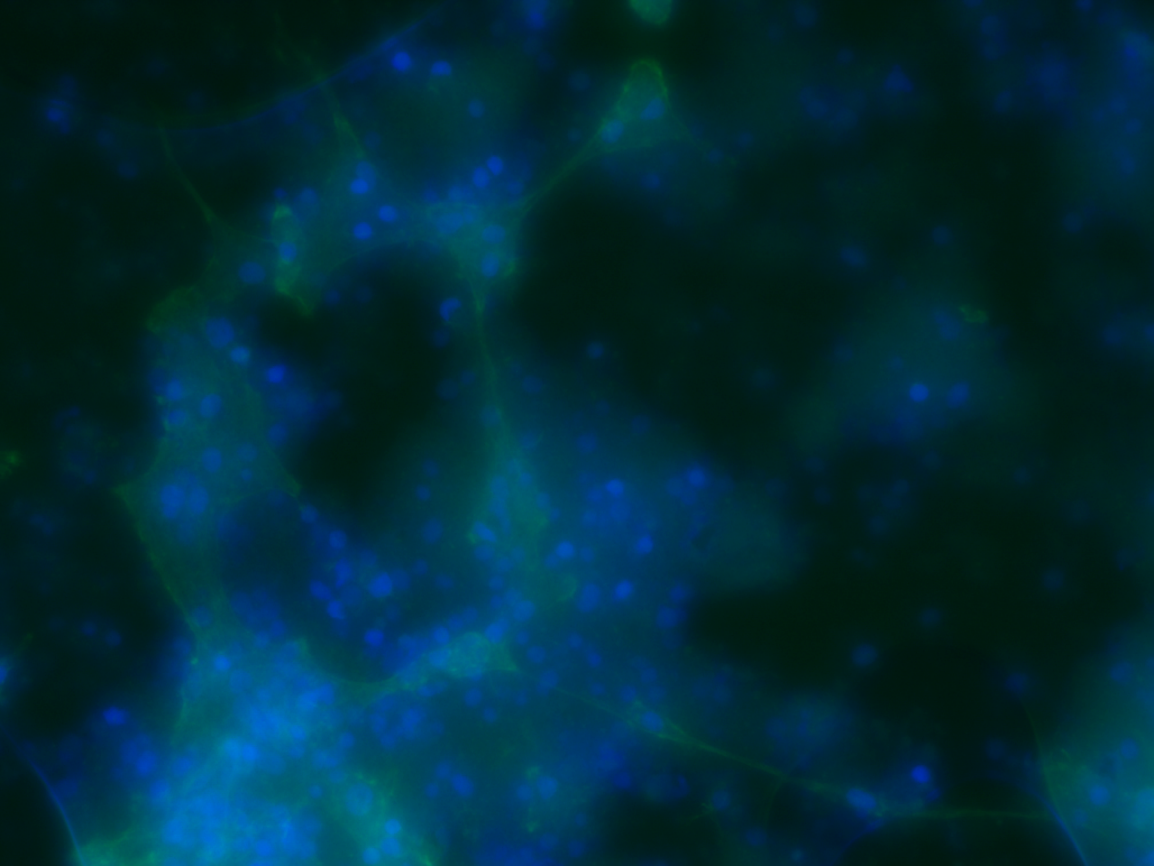


**Supporting Information Figure 7.4** Phalloidin + DAPI staining of cells ($S_{4}$).


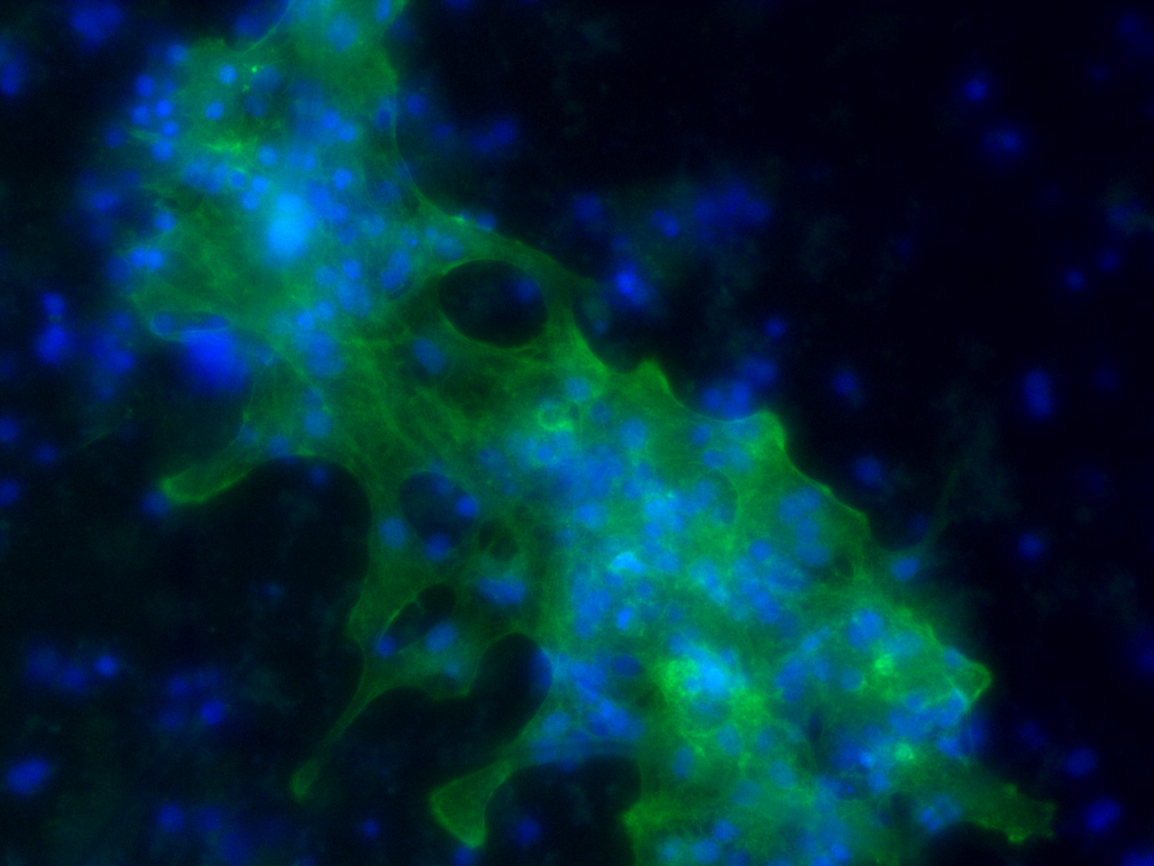


**Supporting Information Figure 7.5** Phalloidin + DAPI staining of cells ($S_{5}$).

**Supporting Information 8***.* *Imaging neurite branching in cultured neuronal networks.*

We present images of synapses and neurite branching in neuronal networks cultured on nanowires substrates from $S_{1}$ to $S_{5}$. In the same image, measured number of synapses to the number of neurons in a region of interest, reveals that neurons are preferentially connected on substrates $S_{3}$ and $S_{4}$, with high values of fractal dimension.

**
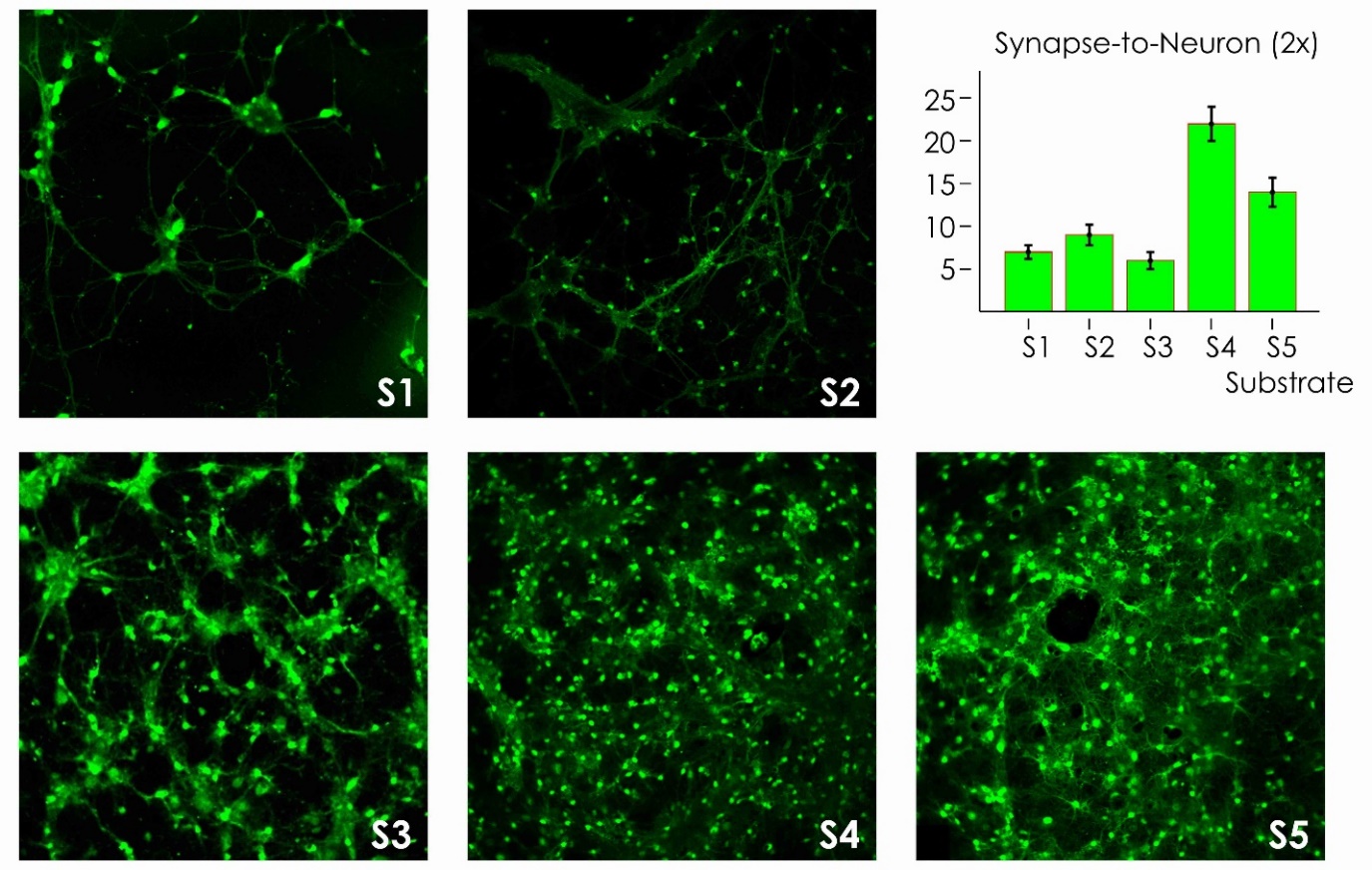
**

**Supporting Information Figure 8.1**

**Supporting Information 9*.*** *Cluster analysis of neural networks on nanowire surfaces.*

Neuron centers were determined for each configuration using image analysis algorithms. To an extent depending on sample number and nanowire density, we observe that neurons are sparsely distributed in space and are preferentially grouped into clusters (**Supporting Information Figure 9.1a**). Emerging patterns in the distributions can be analyzed using convenient clustering algorithms. For each cell distribution, we partitioned elements into groups using a density based clustering algorithm^10^. The algorithm classifies elements into categories on the basis of their similarity. Cluster centers are determined as those points (cells) in the set with higher density than their neighbors and by a relatively large distance from points (cells) with higher densities (**Supporting Information Figure 9.1b**). These points are the cluster centers (**Supporting Information Figure 9.1c**). Upon determination of cluster centers, remaining points are assigned to specific clusters on the basis of their distances to the clusters. In the **Supporting Information Figure 9.1d**, points are colored according to the cluster to which they are assigned. Black points belong to the cluster halos.


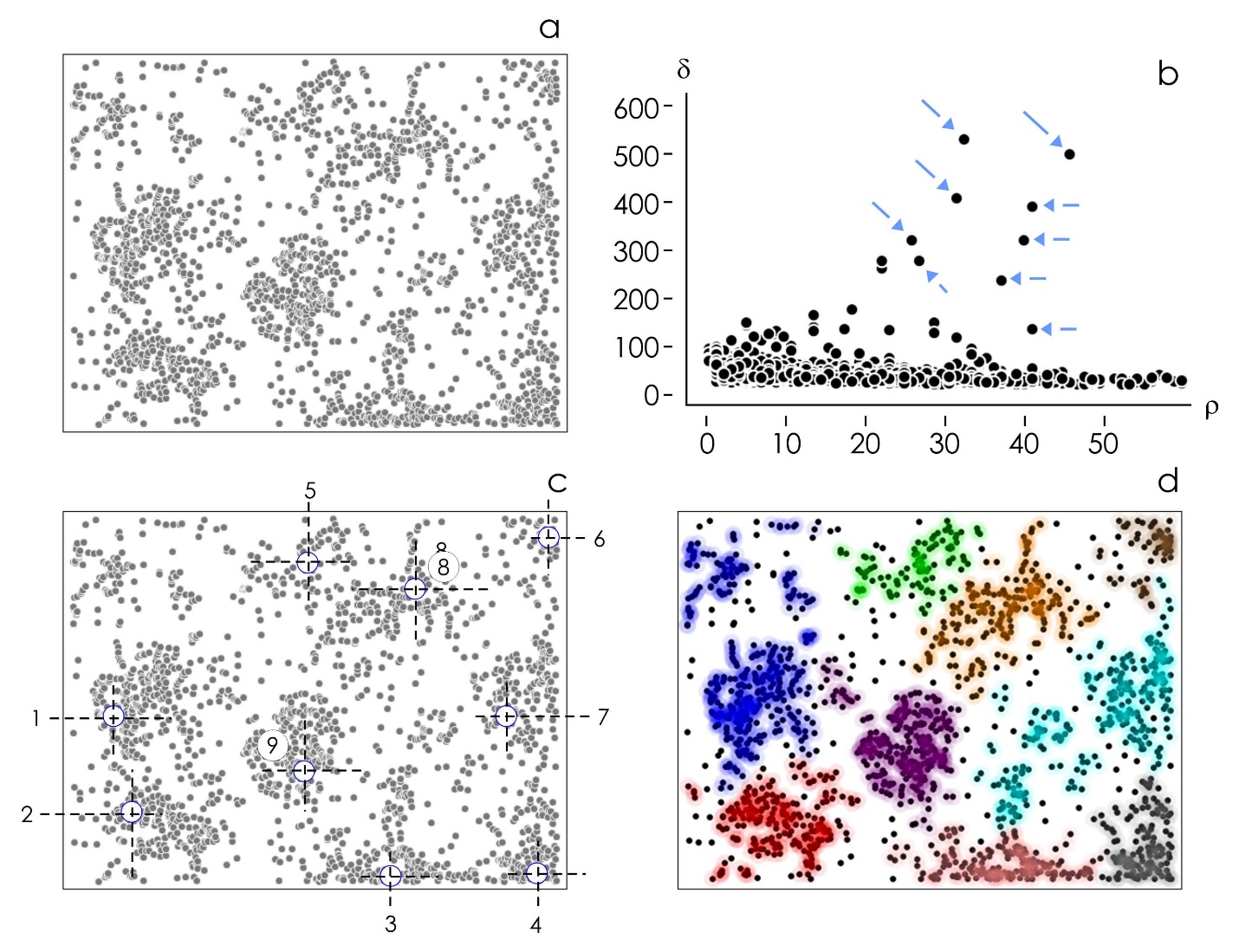


**Supporting Information Figure 9.1**

**Supporting Information 10*.*** *Non linear fit and confidence levels of neuronal cell densities.*

We report the maximum cluster size measured on zinc oxide nanowires substrates for different substrate preparations. In the same **Supporting Information Figure 10.1**, we report raw data, model fit, and confidence levels resulting from the non linear fit of data with the model $n\left( x \right)=C+A \left( 1-B e^{-(x-o)} \right)$.


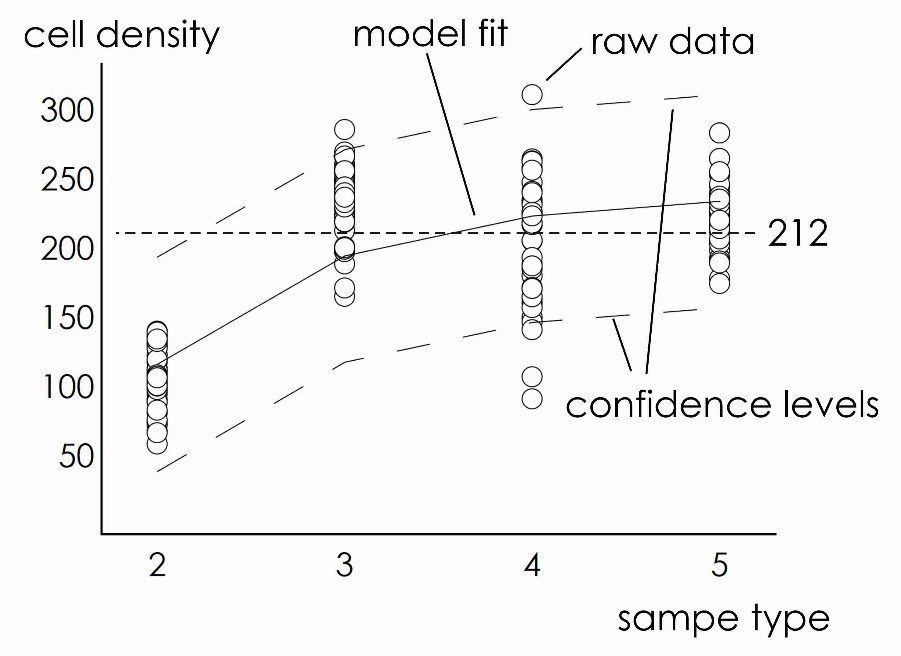


**Supporting Information Figure 10.1**

**Supporting Information 11*.*** *Free energy landscape of neural clusters and maximum allowed cluster size.*

We, we derive the potential energy of systems of cell on a substrate as a function of cluster size and determine the effect of cluster size and cell motility on the stability of the cluster. We find that maximum allowed cluster size is a result of the competition between cell-cell binding forces and forces exerted by the cells on the substrate during migration. We generated randomly distributed sets of points on a surface. Each point represents a neuronal cell on a plane. In generating neural clusters, we maintained constant cell density as $\sigma=2000$ $cells/mm^{2}$ while we varied the size of the cluster in the $n=1-1000 \mathrm{cells}$ interval. We then introduced a probing cell $j$ in the cluster and calculated the potential energy $e$ produced by the entire system on that cell:

| $e=\sum_{i=1}^{n} \frac{k_{s} {(\delta_{ij}-r_{e})}^{2}}{2}, \delta_{ij}<\delta^{max}$ | (SI 11.1) |
| --- | --- |

where ${k_{s}\delta_{ij}^{2}}/2$ is an harmonic potential associated to the cell pair $ij$, $\delta_{ij}$ is the Euclidian distance between cell $i$ and cell $j$, $r_{e}$ is a position of equilibrium, $k_{s}$ is the effective spring constant of the structural linkages between cells, $\delta^{max}$ is the maximum allowed separation distance between cells before link *disruption*. The potential describes the chemical energy of interaction between cells due to specific (cell adhesion molecules, CAM, mediated adhesion) and not specific (electrostatics, electrodynamics, van der Waals) adhesion forces ^11-13^. $e$ would depend on the position of $j$ on the domain, thus, moving the probing cell from the center to the periphery of the cluster, we may derive the free energy landscape of the system as a function of the generalized coordinate $\rho$. $\rho$ is the distance from cluster center in fractions of cluster diameter units. We observe that the energy landscape of the cluster depends on cluster size (**Supporting Information Figure 11.1**). Moreover, depending on the number of elements within the cluster, the difference of potential energy $\Delta e$ between center and border of the cluster varies to a great extent. We found that calculated values of energy barrier $\Delta e$ vary between $\Delta e\sim20 \mathrm{pJ}$ for $n=30$ to $\Delta e\sim0 \mathrm{pJ}$ for $n>600$ cells in the cluster. Height of the barrier $\Delta e$ is proportional to the adhesion strength of individual cells to the cluster and ${F_{e}=\partial e}/{\partial\rho}$ is the resisting force that in any position a cluster exerts on cell to prevent its evasion. Thus $F_{e}$ can be determined from the energy landscape of individual clusters (**Supporting Information Figure 11.2**). For each cluster size, we can find the maximum value of $F_{e}$, $\max(F_{e})=F_{o}$, in the cluster. $F_{o}$ is the value of propelling force that a cell should exert to break anchorages and evade from the cluster. **Supporting Information Figure 11.3** reports calculated values of $F_{o}$ as a function of cluster size. Since cluster size is experimentally observable (i.e. average cultured neural cluster size determined through fluorescence microscopy and clustering analysis algorithms), diagram in the **Supporting Information Figure 11.3** is akin to a *design map* that can support scientists to estimate values of cell motility forces on a substrate. For the present configuration, measured value of cluster size is $\mathrm{cs}=200 \mathrm{cells}$ that, combined with the **Supporting Information Figure 11.3**, yields an estimated value of *force* during adhesion dependent migration of neurons on nanowire surfaces of $F_{m}\sim600 \mathrm{pN}$. This value falls within the same dimensional range of measured values of propelling forces ($1-10000 \mathrm{pN}$) and stresses ($\sim100 \mathrm{Pa}$) of adherent cells on solid surfaces^14-16^.


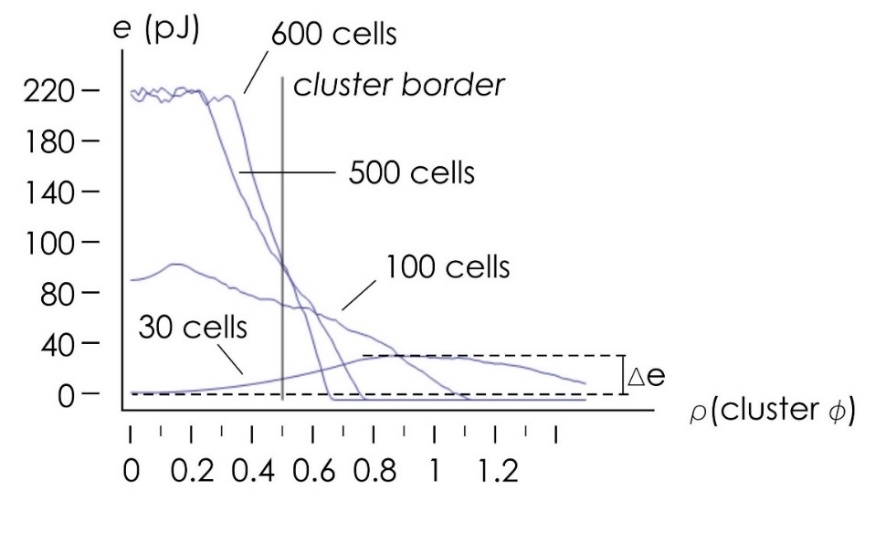


**Supporting Information Figure 11.1**


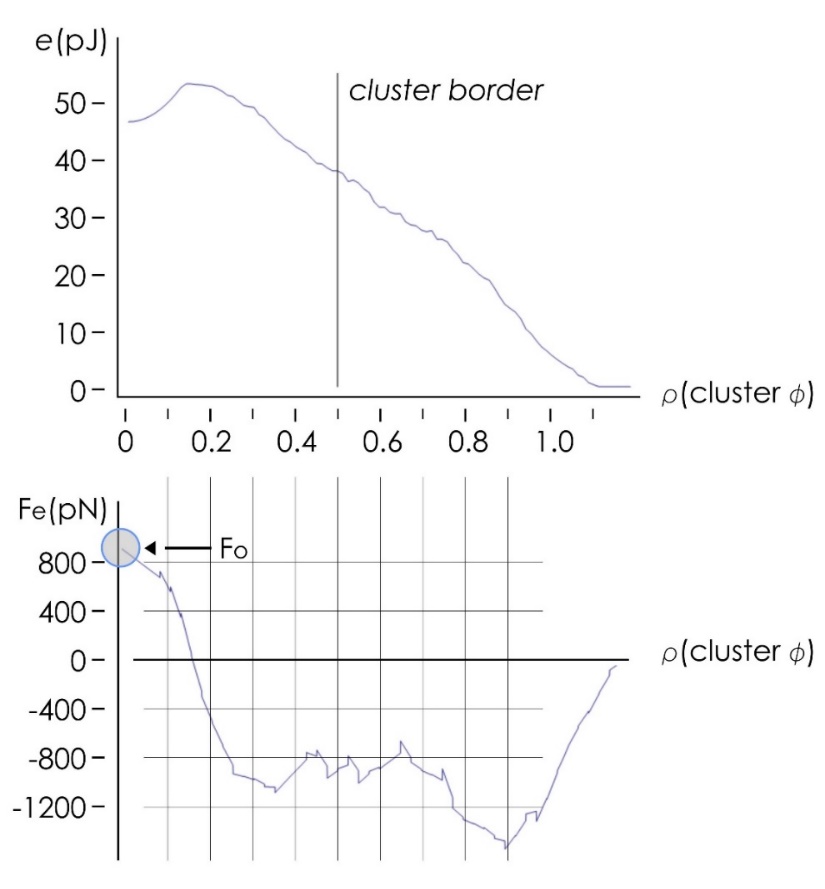


**Supporting Information Figure 11.2**


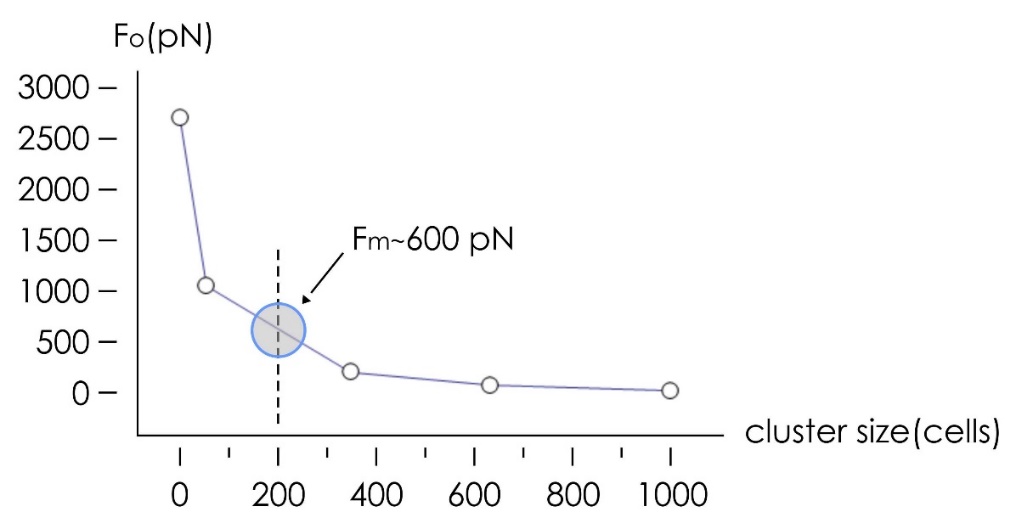


**Supporting Information Figure 11.3**

**Supporting Information 12*.*** *The physical mechanisms of cluster formation*.

The behavior of cells on a substrate is determined by a complex interplay between the cell-cell and the cell-substrate interaction forces^11-13,17-19^. Since the potential energy of a set of cells is minimized for compact arrangements of those cells^20^, when left free of evolve, neuronal cells would likely accumulate into clusters. The characteristics of the substrate would either facilitate or prevent cell clustering. In previous works it has been observed that substrates with intermediate values of roughness and high values of fractal dimension enhance cell clustering^20,21^.

*Roughness* ($Ra$) is necessary to take cells out of equilibrium – it develops those forces that perturb the system, break equilibrium and make the systems collapse into energetically favorable small-world configurations.

*Fractal dimension* ($D_{f}$) quantifies the degree of complexity of a surface. Surfaces with high values of fractal dimension have details over multiple scales - every subset of the original topological space contains an infinite number of points with similar characteristics of the original set. $D_{f}$ provides means to measure how densely a property is expressed along a surface.

Since roughness is determined through a mathematical operation of *average*, it encodes more the *extensive* characteristics of a material, and less the *intensive* characteristics revealed by that material at all scales. Surfaces with high values of roughness may be *flat* on a local scale without appreciable effects on a cell. However, surfaces that have both high $Ra$ and high $D_{f}$, have cracks, rifts or holes distributed along the entire profile of the surface – and if they enable cell motility and cell assembly at a certain scale, it is probable that would continue to do that indefinitely until complete formation of the networks. *Fractal dimension is a measure of how much roughness is ubiquitous along a profile*.

To examine how fractal dimension influences cell clustering, we use a revised version of the mono-dimensional model already introduced in references^22,23^

| $\frac{\partial u}{\partial t}=\frac{\partial^{2}u}{\partial x^{2}}-\frac{\partial u K\left( u \right)}{\partial x}+\Upsilon\eta\left( x \right) \alpha_{2}\left( t \right) \Lambda\left( u \right)$ | (SI 12.1) |
| --- | --- |

In Equation (SI 11.1), $u=u(x,t)$ is the cell density function of space ($x$) and time ($t$), $u K\left( u \right)$ encodes the *cell-cell forces* proportional to the parameter$\xi$, $\Upsilon\eta\left( x \right) \alpha_{2}\left( t \right) \Lambda$ describes the *interaction between the substrate and the cells*, proportional to $\Upsilon$ – the larger the roughness of a surface, the larger $\Upsilon$. In the equation, $\eta$ is a random function of $x$ – it incorporates the contribution of fractal dimension. For small values of fractal dimension, $\eta$ is nearly zero. For high values of fractal dimension, $\eta$ is an array of ones, extending the effects of $\Upsilon$ to all length scales.

Using a finite differences numerical scheme^23^, we solved Equation (SI 12.1) to obtain the steady state value of cell density for fixed values of $\Upsilon/\xi=2$ (roughness induced forces to the cell-cell adhesion force) and varying $\eta$. Results, reported in the **Supporting Information Figure 12.1**, indicate that for small values of $\eta$ and fractal dimension ($\eta<0.3$) cells remain uniformly distributed on a substrate. For high values of $\eta$ and fractal dimension ($\eta>0.3$) cells assemble into clusters with multiple peaks in the cell density distribution.


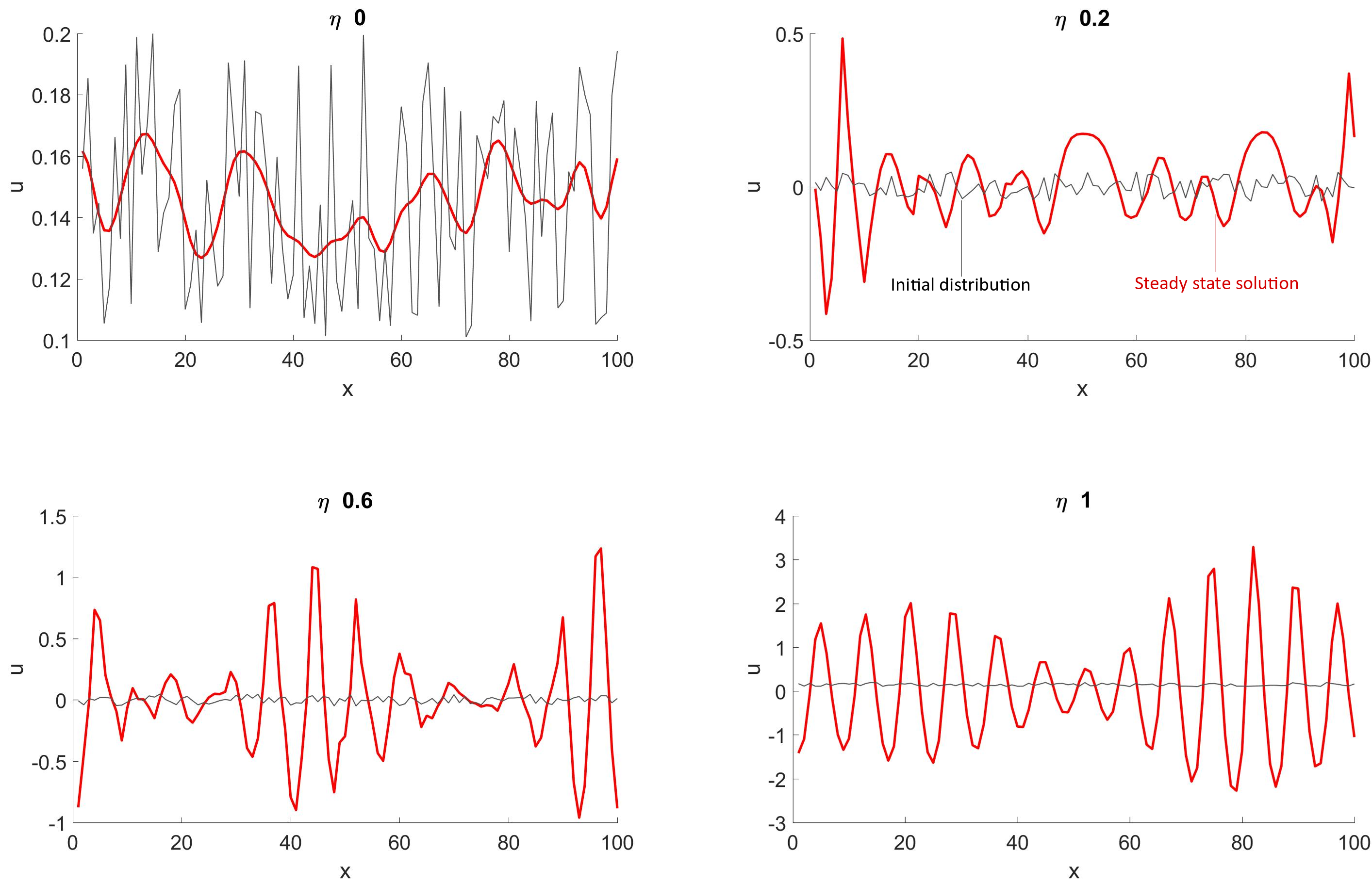


**Supporting Information Figure 12.1**

**References**

1 Lindroos, S. & Leskela, M. Growth of zinc peroxide (ZnO2) and zinc oxide (ZnO) thin films by the successive ionic layer adsorption and reaction – SILAR – technique. *International Journal of Inorganic Materials* **2**, 197–201 (2000).

2 Umar, A., Karunagaran, B., Suh, E.-K. & Hahn, Y. B. Structural and optical properties of single-crystalline ZnO nanorods grown on silicon by thermal evaporation. *Nanotechnology* **17** (2006).

3 Villani, M. *et al.* Turning carbon fiber into a stress-sensitive composite material. *Journal of Materials Chemistry A* **4**, 10486-10492 (2016).

4 Xu, S. & Wang, Z. L. One-dimensional ZnO nanostructures: Solution growth and functional properties. *Nano Research* **4**, 1013–1098 (2011).

5 Zuiderveld, K. in *Graphic Gems IV* 474–485 (Academic Press Professional, 1994).

6 Otsu, N. A Threshold Selection Method from Gray-Level Histograms. *IEEE Transactions on Systems, Man, and Cybernetics* **9**, 62-66 (1979).

7 Narula, V., Zippo, A. G., Muscoloni, A., Biella, G. E. M. & Cannistraci, C. V. Can local-community-paradigm and epitopological learning enhance our understanding of how local brain connectivity is able to process, learn and memorize chronic pain? *Applied Network Science* **2**, 1-28 (2017).

8 Chen, C.-H., He, C.-W., Liao, C.-P. & Pan, C.-L. A Wnt-planar polarity pathway instructs neurite branching by restricting F-actin assembly through endosomal signaling. *PLoS Genetics* **13**, e1006720 (2017).

9 Zhao, B. *et al.* Microtubules Modulate F-actin Dynamics during Neuronal Polarization. *Scientific Reports* **7** (2017).

10 Rodriguez, A. & Laio, A. Clustering by fast search and find of density peaks. *Science* **344**, 1492–1496 (2014).

11 Bell, G. I. Models for the specific adhesion of cells to cells. *Science* **618**, 618-627 (1978).

12 Evans, E. A. & Calderwood, D. A. Forces and Bond Dynamics in Cell Adhesion. *Science* **316**, 1148-1153 (2007).

13 Sackmann, E. & Smith, A.-S. Physics of cell adhesion: some lessons from cell mimetic systems. *Soft Matter* **10**, 1644–1659 (2014).

14 Ananthakrishnan, R. & Ehrlicher, A. The Forces Behind Cell Movement. *International Journal of Biological Sciences* **3**, 303-317 (2007).

15 Schwarz, U. S. Physics of adherent cells. *Reviews of Modern Physics* **85**, 1327-1381 (2013).

16 Trepat, X. *et al.* Physical forces during collective cell migration. *Nature Physics* **5**, 426-430 (2009).

17 Decuzzi, P. & Ferrari, M. Modulating cellular adhesion through nanotopography. *Biomaterials* **31**, 173–179 (2010).

18 Gentile, F. *et al.* Selective modulation of cell response on engineered fractal silicon substrates. Scientific Reports 2013, 3, 1461. *Scientific Reports* **3**, 1461 (2013).

19 Stevens, M. & George, J. Exploring and engineering the cell surface interface. *Science* **310**, 1135-1138 (2005).

20 Onesto, V. *et al.* Nano-topography Enhances Communication in Neural Cells Networks. *Scientific Reports* **7**, 1-13 (2017).

21 Marinaro, G. *et al.* Networks of Neuroblastoma Cells on Porous Silicon Substrates Reveal a Small World Topology. *Integrative Biology* **7**, 184-197 (2015).

22 Armstrong, N. J., Painter, K. J. & Sherratt, J. A. A continuum approach to modelling cell–cell adhesion. *Journal of Theoretical Biology* **243**, 98–113 (2006).

23 Onesto, V., Narducci, R., Amato, F., Cancedda, L. & Gentile, F. The effect of connectivity on information in neural networks. *Integrative Biology* **10**, 121-127 (2018).
